# Toolbox of Advanced Atomic Layer Deposition Processes for Tailoring Large-Area MoS_2_ Thin Films at 150 °C

**DOI:** 10.1021/acsami.3c02466

**Published:** 2023-07-17

**Authors:** Miika Mattinen, Jeff J. P. M. Schulpen, Rebecca A. Dawley, Farzan Gity, Marcel A. Verheijen, Wilhelmus M. M. Kessels, Ageeth A. Bol

**Affiliations:** † Department of Applied Physics and Science Education, Eindhoven University of Technology, PO Box 513, Eindhoven 5600 MB, The Netherlands; § Department of Chemistry, University of Michigan, 930 North University Avenue, Ann Arbor, Michigan 48109-1055, United States; ⊥ Tyndall National Institute, University College Cork, Lee Maltings, Dyke Parade, Cork T12 R5CP, Ireland; ∥ Eurofins Materials Science Netherlands, High Tech Campus 11, Eindhoven 5656 AE, The Netherlands

**Keywords:** 2D materials, atomic layer deposition, electrocatalysis, electronics, low-temperature processing, MoS_2_

## Abstract

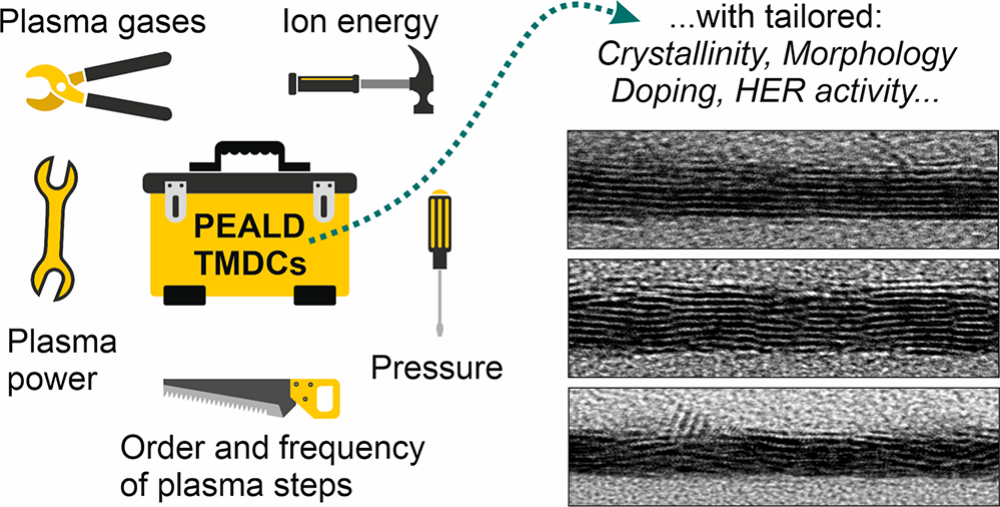

Two-dimensional MoS_2_ is a promising material for applications, including electronics and electrocatalysis. However, scalable methods capable of depositing MoS_2_ at low temperatures are scarce. Herein, we present a toolbox of advanced plasma-enhanced atomic layer deposition (ALD) processes, producing wafer-scale polycrystalline MoS_2_ films of accurately controlled thickness. Our ALD processes are based on two individually controlled plasma exposures, one optimized for deposition and the other for modification. In this way, film properties can be tailored toward different applications at a very low deposition temperature of 150 °C. For the modification step, either H_2_ or Ar plasma can be used to combat excess sulfur incorporation and crystallize the films. Using H_2_ plasma, a higher degree of crystallinity compared with other reported low-temperature processes is achieved. Applying H_2_ plasma steps periodically instead of every ALD cycle allows for control of the morphology and enables deposition of smooth, polycrystalline MoS_2_ films. Using an Ar plasma instead, more disordered MoS_2_ films are deposited, which show promise for the electrochemical hydrogen evolution reaction. For electronics, our processes enable control of the carrier density from 6 × 10^16^ to 2 × 10^21^ cm^–3^ with Hall mobilities up to 0.3 cm^2^ V^–1^ s^–1^. The process toolbox forms a basis for rational design of low-temperature transition metal dichalcogenide deposition processes compatible with a range of substrates and applications.

## 1 Introduction

Two-dimensional (2D) materials rank high among the most scientifically and technologically interesting materials of the early 21st century. Among them, the large family of transition metal dichalcogenides (TMDCs) that includes semiconductors, (semi)metals, and even superconductors^1,2^ has drawn intense attention. Besides stability as single, subnanometer thick monolayers, their extraordinary electronic, optical, mechanical, and chemical properties give TMDCs great potential in a range of applications including (opto)electronics, energy storage, and catalysis.^3−5^ Some 30 different 2D TMDC materials are known that share the composition MX_2_, where M is a transition metal or Sn and X is S, Se, or Te. The majority of studies have focused on semiconducting TMDCs, such as MoS_2_ and WS_2_, which are among the most promising alternatives to silicon for future nano(opto)electronics.^6−9^ The flexible nature of 2D materials also makes them promising for flexible devices including displays and various sensors.^10,11^ TMDCs have also been intensively studied for the electrocatalytic hydrogen evolution reaction, a key process in the production of clean hydrogen, where it promises an affordable and sustainable alternative to currently used, scarce Pt.^12−16^ Hydrogen is expected to play a major role in the carbon-neutral future, offering a clean, highly efficient fuel, means for grid stabilization, as well as a green alternative for carbon in steel production, for example.^17^

One of the major factors hindering applications of TMDCs in nanoelectronics, electrocatalysis, and other fields alike is the lack of scalable fabrication methods.^5,18^ In general, continuous, high-quality films with a controlled and uniform thickness over large, sometimes 3D structured substrates are called for. Many substrates and process flows also place limitations on the applicable thermal budget. Initially, 2D monolayers were produced by mechanical exfoliation of bulk crystals.^19^ Although this method remains in use in research for its simplicity and ability to produce high-quality flakes, it cannot be scaled up for production. Chemical vapor deposition (CVD) has emerged as a widespread method for the preparation of high-quality TMDC flakes and films. Its most common variant utilizing chalcogenide and metal oxide powder precursors^2,20−22^ struggles with thickness control and uniformity and requires high temperatures from 600 to 1000 °C. Use of metalorganic and halide metal precursors and gaseous chalcogenide sources improves scalability and enables a decrease of the deposition temperature to 400–550 °C,^23−25^ in favorable cases even 200–300 °C.^26,27^ These processes are compatible with back-end-of-line semiconductor processing (*T*_max_ ≈ 400–500 °C),^28^ but cannot be applied on typical plastics and other sensitive materials used in the emerging field of flexible electronics (*T*_max_ ≈ 100–200 °C). Low processing temperatures also simplify deposition equipment design and require less energy input, which is attractive from both economic and environmental points of view.

Atomic layer deposition (ALD) is an advanced, surface-controlled variant of CVD that boasts excellent uniformity on large areas and complex shapes, accurate thickness control, and high reproducibility and scalability.^29,30^ Recently, ALD has been increasingly studied for deposition of TMDCs for a variety of applications.^31,32^ ALD processes operate at relatively low temperatures, generally below 500 °C. This is particularly true for its plasma enhanced variant (PEALD) that takes advantage of highly reactive radicals and low-energy ions.^33−35^ PEALD processes depositing semiconductors MoS_2_ and WS_2_ at 300–450 °C have been developed in our group,^36,37^ while metallic TiS_2_ films could be deposited even at 150 °C.^38^

Recently, we showed that tailoring the plasma deposition chemistry enables a decrease of deposition temperature of both semiconducting and metallic TMDCs to record low 100 °C.^39^ The deposition of stoichiometric and crystalline MoS_2_, TiS_2_, and WS_2_ films was achieved by addition of sufficient hydrogen to the H_2_S plasma feed gas, which prevents excess S incorporation from otherwise happening at these low temperatures. However, tailoring the chemistry of a single plasma step in such a manner offers limited control over film properties, as the plasma species play multiple simultaneous roles including removal of ligands of the metal precursor, deposition of sulfur, and supplying energy in the form of low-energy ions and other reactive species for creation and healing of defects.

In this study, we show that improved control over film growth and resulting properties can be achieved by using two separate and individually optimized plasma steps. While the first plasma step removes ligands of the metal precursor and supplies sulfur, the second step can be further tailored to control stoichiometry, crystallinity, and electrical properties, among others. We have developed three processes utilizing H_2_ and Ar plasma steps that deposit uniform, crystalline MoS_2_ films of accurately controlled thickness at a very low temperature of 150 °C. We discuss the growth mechanisms as well as effect of plasma conditions for each process. Compared to earlier (PE)ALD processes, the new processes exhibit complementary and in many cases improved properties as shown by systematic use of a broad range of microscopy, structural, compositional, and electrical characterization techniques. For electronics, carrier densities can be decreased by 3–5 orders of magnitude, and Hall mobilities increased by an order of magnitude compared to our earlier low-temperature process. For electrocatalytic HER, the catalytic activity can be tailored via changes in the film morphology and crystallinity using the process toolbox developed in this work.

## 2 Results and Discussion

### 2.1 Concept of Developed PEALD Processes

**Figure 1:**
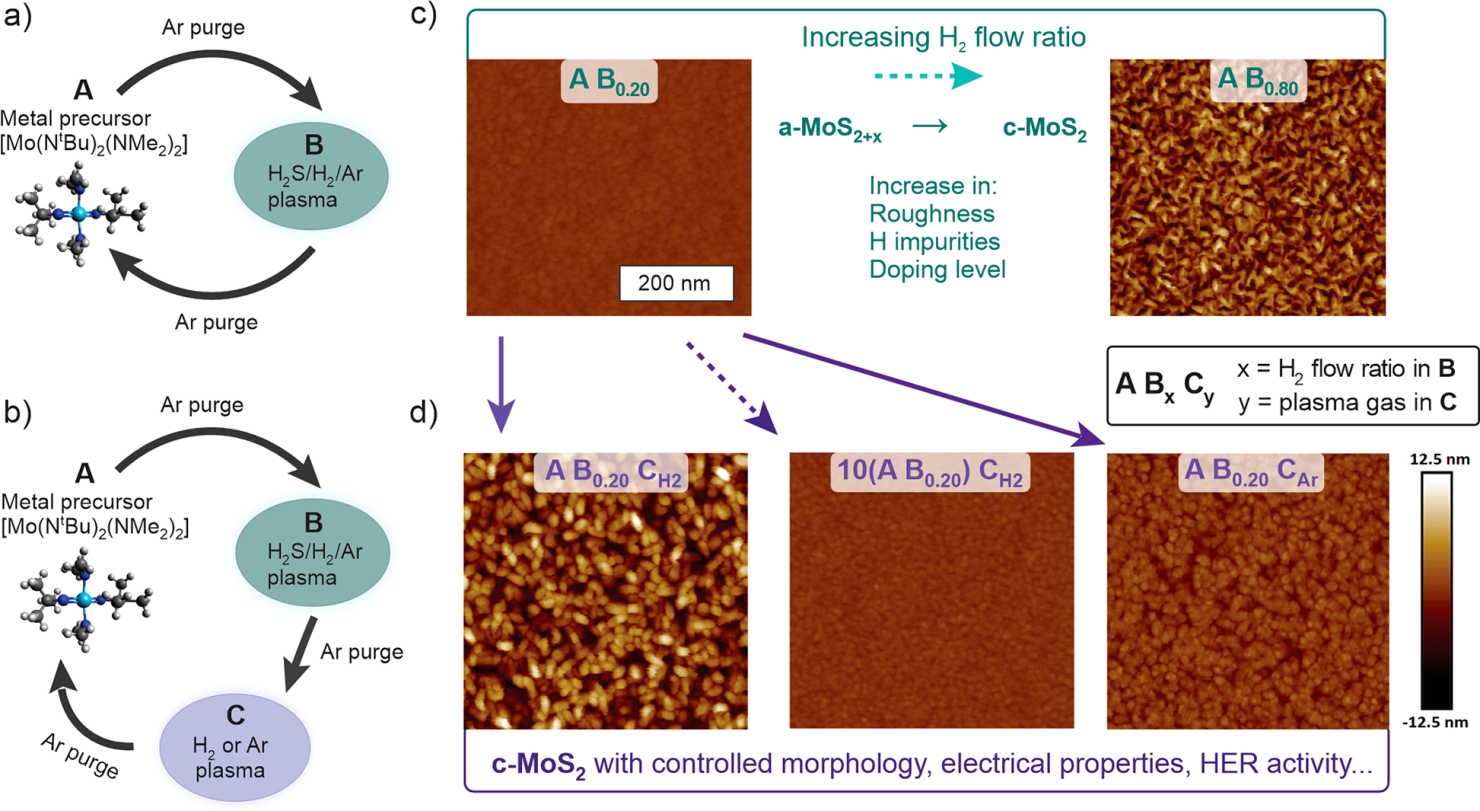
Schematic of (a) AB and (b) ABC type PEALD processes and illustration of how the (c) AB and (d) ABC processes enable tailoring of film properties including crystallinity (c = crystalline, a = amorphous) and morphology (atomic force microscopy, AFM, images).

In this contribution, we explore the ample opportunities that PEALD offers for tailoring TMDC film growth and properties via rational process design and control of process conditions. A typical PEALD process illustrated in Figure 1a begins by exposing a substrate to a metal precursor (denoted A step), in our case a metalorganic Mo precursor Mo(N^t^Bu)_2_(NMe_2_)_2_, which reacts on the substrate surface in self-limiting manner. Then, excess unreacted precursor and byproducts are purged away with the help of Ar gas. The following B step consists of exposure to a plasma, here a mixed H_2_S/H_2_/Ar plasma, which removes the ligands of the adsorbed Mo precursor and supplies S, again in a self-limiting manner. A second purge completes an “AB” ALD cycle, which can then be repeated until the target thickness is reached. In such an AB cycle, the plasma conditions of the B step can be varied to tailor film growth and properties. An example of this from our earlier work is shown Figure 1c, illustrating how increasing H_2_ flow ratio (eq 1) from 0.20 to 0.80 at 150 °C changes the resulting film from amorphous MoS_3.5_ to crystalline MoS_2_.^39^ However, the increase in the H_2_ flow ratio also leads to incorporation of H dopants into the films, resulting in high carrier densities, a drawback for many semiconductor applications. In addition, high H_2_ flow ratios, which we denote as a subscript of B (e.g., B_0.80_) lead to rough morphology (Figure 1c). Using intermediate H_2_ flow ratios (e.g., B_0.65_) results in lower roughness and H impurities, but also poorer crystallinity and electrical properties.^39^ These examples illustrate limitations of the AB approach.
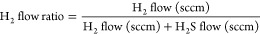
1In the “ABC” approach pursued in this work (Figure 1b), we use two separate plasma steps, which allow for more effective decoupling of different atomistic processes and therefore enhanced control over growth and properties of MoS_*x*_ films. The first plasma step (B step) is optimized for *deposition*, namely, removing ligands of the adsorbed metal precursor and sulfur deposition. Here, we typically use a low H_2_ flow ratio in the B step (B_0.20_) to limit H incorporation into the films. The second plasma step (C step) is then used for *modification*, that is, removal of excess S (if present), modifying crystallinity, and tuning film properties. The plasma feed gas in this step is denoted as a subscript of C (e.g., C_Ar_). Thus, we refer to our process with an Ar plasma modification step, for example, as the A B_0.20_ C_Ar_ process.

We have used three different approaches to control the C step: (1) using different plasma gases (H_2_, Ar, H_2_/Ar, and H_2_S/Ar), (2) modifying plasma conditions (such as exposure time, pressure, and plasma power), and (3) changing the frequency or periodicity of the C step, i.e., only applying it every *n* ALD cycles (denoted *n*(A B_*x*_) C_*y*_). Furthermore, each C step can be combined with different plasma compositions and parameters in the B step, different deposition temperatures, and so forth, giving rise to near infinite possibilities as well as conditions to investigate.

Figure 1d illustrates three optimized processes that can be used to deposit c-MoS_2_ films of varying stoichiometries, crystallinities, morphologies, and electrical properties. In these processes that form the core of our “toolbox”, an A B_0.20_ cycle depositing MoS_3.5_ films was combined with an H_2_ plasma step either every cycle (A B_0.20_ C_H2_) or every 10 cycles (10(A B_0.20_) C_H2_)) or an Ar plasma step in each cycle (A B_0.20_ C_Ar_) to remove the excess S and crystallize the films. A schematic of the ALD processes is shown in Figure S1 (Supporting Information). Throughout the manuscript, we discuss processes depositing MoS_2_ at a fixed temperature of 150 °C. The concepts are regardless applicable to other low temperatures, as we show for the A B_0.20_ C_H2_ process, as well as other TMDCs.

These ABC processes allow for submonolayer level thickness control by simply changing the number of ALD cycles (Figure S2 in the Supporting Information) and result in excellent wafer scale uniformity (∼2% thickness standard deviation over a 4 in. wafer, Figure S3).

In Section 2.2, we discuss the optimization and mechanisms of these processes. Other explored ABC processes, including those of A B_0.80_ C_*y*_ type where the C step is used to modify an already crystalline MoS_2_ film, are described in the Supporting Information (Section S5). In Section 2.3, we compare the characteristics of films deposited by using the different ABC processes. Finally, in Section 2.4, we explore the application of our MoS_2_ films as HER catalysts.

### 2.2 Characteristics and Optimization of Three ABC Processes

#### 2.2.1 Using H_2_ Plasma: A B_0.20_ C_H2_ process

**Figure 2:**
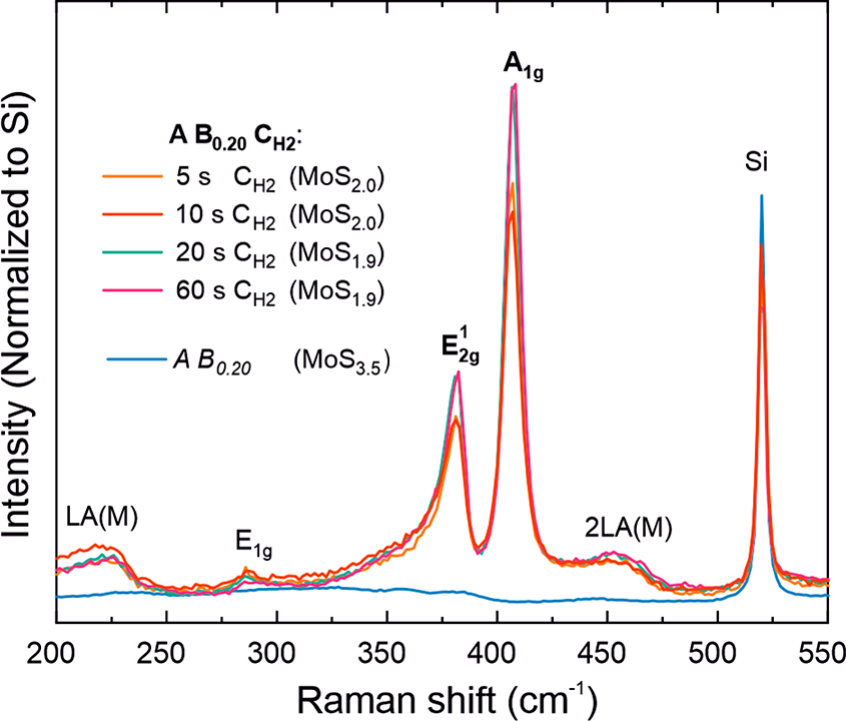
Raman spectra and XPS-derived stoichiometries showing the efficient and self-limiting character of the H_2_ plasma C step in the deposition of c-MoS_2_ films. The main Raman modes of MoS_2_ are shown in bold and defect-related and Si modes in normal type (for clarity, LO, TO, and ZO modes are not indicated). The films were deposited by using 140 ALD cycles at 150 °C.

The idea of using H_2_ plasma in a C step builds on our earlier finding of hydrogen preventing excess sulfur incorporation, which otherwise occurs during low-temperature PEALD of TMDCs.^39^ Using H_2_S plasma with a low H_2_ flow ratio such as 0.20 yields a-MoS_3.5_ films at 150 °C. In contrast, the A B_0.20_ C_H2_ process results in crystalline c-MoS_1.9_ films at 150 °C as shown by the characteristic E^1^_2g_ and A_1g_ Raman modes of MoS_2_ and stoichiometries determined by XPS (Figure 2). This shows that separate H_2_ plasma exposure efficiently removes the excess sulfur present in a-MoS_3.5_ and allows for the resulting MoS_2_ to crystallize. Along with the removal of excess S, the resistivity of the films decreased from >4000 to 3–13 Ω cm and the growth per cycle (GPC) increased from 0.65 to 1.0–1.2 Å. The process showed self-limiting characteristics as expected for ALD in terms of the GPC, S/Mo ratio, resistivity, and Raman intensity (Figure 2 and Section S2.1 in the Supporting Information). A 20 s H_2_ plasma exposure was deemed saturating and was chosen for further experiments. Besides the temperature of 150 °C discussed here, we found that the process deposited crystalline MoS_2_ films at temperatures ranging from 100 to 250 °C (Section S2.2 in the Supporting Information).

Using H_2_ gas instead of H_2_ plasma had no effect on the stoichiometry or crystallinity of a-MoS_3.5_, showing that plasma-generated species such as radicals and ions are essential to the process (Table S1). Using H_2_ plasma, the crystallinity was observed to decrease when pressure was increased (Figure S5); thus, the lowest pressure allowing for reliable striking of H_2_ plasma (20 mTorr) was used in further experiments. The pressure dependence suggests that while H radicals likely play a major role in changing stoichiometry from MoS_3.5_ to MoS_1.9_, low-energy ions, such as H_3_^+^,^40^ may have a beneficial effect on crystallinity.^35,41^

**Figure 3:**
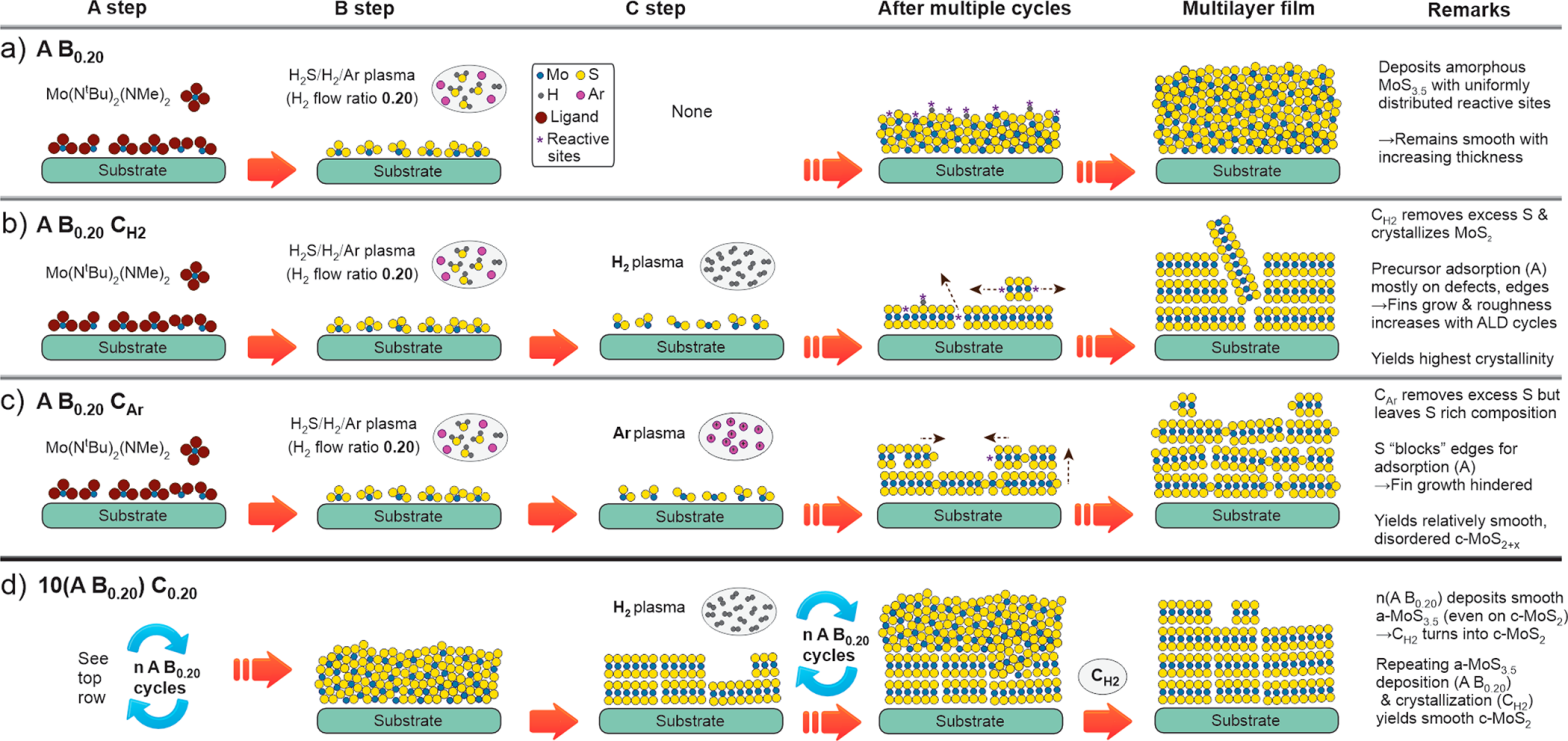
Schematic illustration of the (crystal) growth mechanisms and their implications to morphology and microstructure for (a) the reference A B_0.20_ process and (b–d) the three ABC processes developed in this work.

While the amorphous, sulfur-rich films deposited by the A B_0.20_ process are smooth (Figure 1c), the crystalline MoS_2_ films obtained with the A B_0.20_ C_H2_ process exhibit a rough surface (Figure 1d). Surface roughness causes charge carrier scattering, which limits electrical performance, and may lead to challenges in integration to nanoscale devices. The large roughness results from out-of-plane oriented grains or “fins” (Section 2.3.1). The edges of TMDC crystallites, including fins, are known to be more reactive compared to their basal planes, as confirmed by both DFT calculations^37^ and experiments.^37,39^ Fins may form through multiple pathways, for example when two laterally growing MoS_2_ crystallites meet each other.^42^ Highly reactive species generated in H_2_ plasma can play a role in both creation and etching of fins.^42^ Unless effectively removed or passivated, the highly reactive fins grow rapidly in the out-of-plane direction, resulting in rough film morphology (Figure 3b).

#### 2.2.2 Using H_2_ Plasma Every *n* Cycles: *n*(A B_0.20_) C_H2_ Process

**Figure 4:**
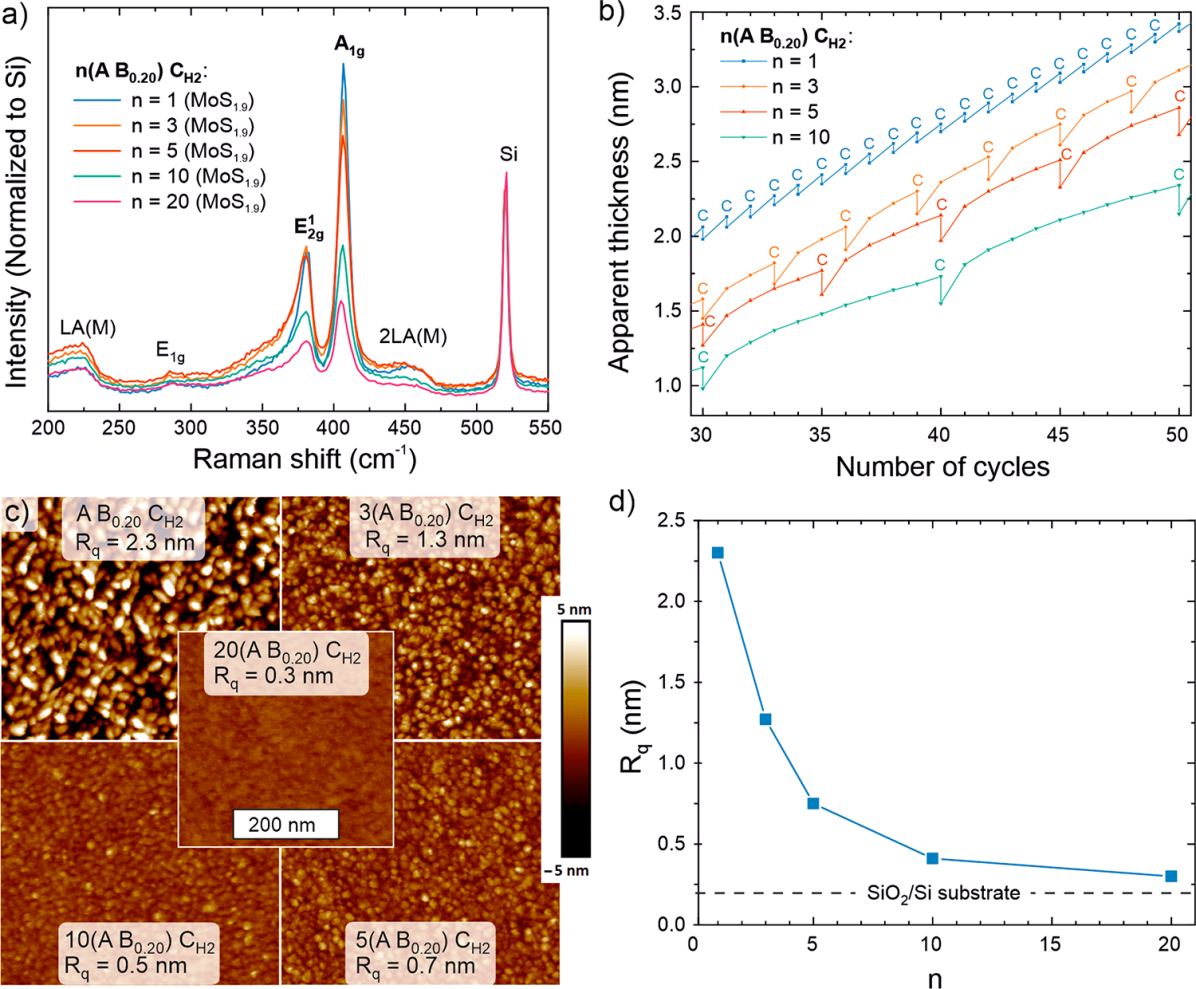
Characterization of the *n*(A B_0.20_) C_H2_ process**:** effect of *n* on (a) crystallinity and S/Mo ratio, (b) film growth (*in situ* SE thicknesses measured after every B and C step; C steps are marked by the letter C), and (c, d) morphology and roughness (AFM). The analyzed films were 6–8 nm thick.

We hypothesized that application of a H_2_ plasma C step once every *n* cycles instead of every cycle, which is denoted as *n*(A B_0.20_) C_H2_, may lead to smoother film morphology. The A B_0.20_ cycles deposit a-MoS_2+*x*_, which lacks the anisotropy of 2D MoS_2_, and may therefore hinder fin growth (Figure 3a). For the *n*(A B_0.20_) C_H2_ process to result in c-MoS_2_ films, periodic H_2_ plasma exposures must be able to remove excess S and crystallize the deposited material as MoS_2_ over the thickness of several Å deposited during *n* A B_0.20_ cycles. Raman spectroscopy and XPS suggest that this is indeed the case, as application of H_2_ plasma every 3, 5, 10, or even 20 A B_0.20_ cycles resulted in crystalline films with MoS_1.9_ composition (Figure 4a).

Further insights into growth mechanisms were sought from *in situ* SE measurements performed twice every cycle, i.e., after each B and C step. In this way, the thickness changes resulting from each plasma exposure could be followed with sub-Å resolution.^43^ The extracted thicknesses are best seen as apparent thicknesses due to the use of fixed c-MoS_1.9_ optical constants that were determined from the end point of each deposition. Nevertheless, the apparent thickness clearly decreased after each H_2_ plasma C step (Figure 4b and Figure S11 in the Supporting Information). This is expected, considering that more than 40% of the S atoms deposited by the *n* A B_0.20_ cycles are removed when the H_2_ plasma step converts a-MoS_3.5_ to c-MoS_1.9_.

The apparent GPC in the first A B_0.20_ cycle following a H_2_ plasma C step was between 1.5 and 2.5 Å, which is much higher than the steady-state GPC of the A B_0.20_ process (0.7 Å). For *n* > 1, the GPC quickly decreased in the following A B_0.20_ cycles, such that the aforementioned steady-state GPC was reached after 5–10 AB cycles. This is in line with a transition from a c-MoS_2_ surface to an a-MoS_2+*x*_ surface when multiple A B_0.20_ cycles are applied (Figure 3d). a-MoS_2+*x*_ is hypothesized to contain a lower density of reactive sites where the Mo(N^t^Bu)_2_(NMe_2_)_2_ precursor can adsorb, which explains the decrease in GPC despite the increase in S/Mo stoichiometry compared to c-MoS_2_.^39^ XPS measurements confirmed that A B_0.20_ cycles indeed deposit MoS_2+*x*_ on a c-MoS_2_ surface (Section S2.3 in the Supporting Information).

AFM showed that the surface roughness decreased with increasing *n*. The roughness of the films deposited using the A B_0.20_ C_H2_ process was high, with features as high as 10 nm observed for a nominally 7 nm thick film (*R*_q_ = 2.3 nm, Figure 4c). TEM confirmed these features to be fins (Section 2.3.1). When *n* was increased, the height of these features decreased together with a decreasing roughness. The roughness of 6–7 nm films deposited by using *n* = 10 and 20 approached that of the substrate (Figure 4d). The decrease in roughness suggests hindered fin growth or formation, in line with the proposed growth mechanisms (Figure 3d).

Analysis of the Raman data shown in Figure 4a and X-ray diffraction (XRD) data in Figure S8 suggested crystallinity to decrease with increasing *n* (see Section 2.3.1 for crystallinity analysis). Considering growth characteristics, morphology, and crystallinity, *n* = 10 was chosen as the preferred H_2_ plasma frequency for obtaining smooth, crystalline MoS_2_ films.

#### 2.2.3 Using Ar Plasma: A B_0.20_ C_Ar_ Process

**Figure 5:**
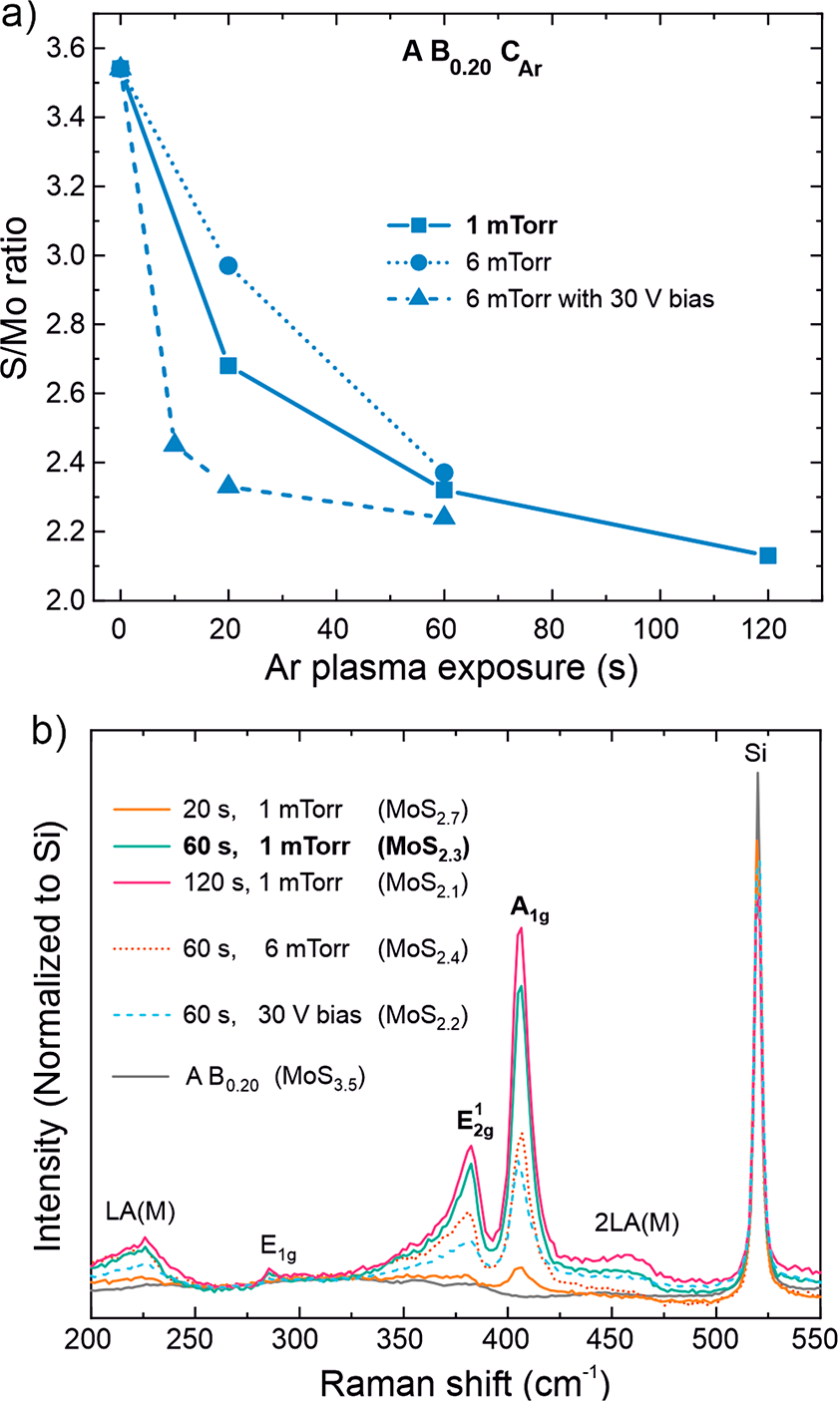
Characterization of the A B_0.20_ C_Ar_ process: effect of Ar plasma conditions and exposure on (a) S/Mo ratio (XPS) and (b) crystallinity (Raman spectroscopy). The films were deposited by using 140 ALD cycles at 150 °C.

Ar^+^ ion bombardment from Ar plasma offers another route to the removal of excess sulfur from a-MoS_2+*x*_ and subsequent crystallization. In order to obtain Ar^+^ ions of sufficient flux and energy from our remote ICP source, low pressures ranging from 1 to 6 mTorr were used. The most efficient removal of excess S and consequently S/Mo ratios closest to 2 as well as the highest degree of crystallinity were achieved at the lowest available pressure of 1 mTorr and long exposure times of at least 60 s (Figure 5a–use of substrate biasing will be discussed below). In particular, MoS_2.3_ and MoS_2.1_ films deposited by using 60 and 120 s Ar plasma exposures at 1 mTorr exhibited clear Raman signatures of c-MoS_2_ (Figure 5b). For further experiments, a 60 s Ar plasma exposure at 1 mTorr was chosen, which resulted in a GPC of 0.8 Å, a relatively smooth morphology, and high resistivity of 220 Ω cm. Further data and discussion on characterization and optimization are provided in Section S2.4 in the Supporting Information.

For an Ar plasma at 1 mTorr, we can estimate based on earlier measurements in a similar reactor^42,44−46^ the mean kinetic energy of Ar^+^ ions at 25–30 eV and the ion flux incident on the substrate at 2–4 × 10^14^ cm^–2^ s^–1^. Combining this information with Rutherford backscattering spectrometry (RBS) data on the areal density of atoms deposited allows us to estimate that on average, ∼100 Ar^+^ ions with a mean kinetic energy of 25–30 eV are needed to remove a single S atom at the optimized 60 s Ar plasma exposure (Section S2.6 in the Supporting Information).

We explored the use of RF substrate biasing^44,47,48^ to increase the energy of Ar^+^ ions and decrease the amount of required ions and processing time. Already a 10 s exposure at 30 V substrate bias (estimated mean ion energy of 45–55 eV) resulted in efficient S removal (Figure 5a) and signs of crystallinity (Section S2.5 in the Supporting Information). However, the crystallinity as inferred from the intensity and width of the MoS_2_ Raman modes remained low within the range of plasma exposures (10 to 60 s) and biasing voltages explored (30 to 60 V). Figure 5b shows the best identified biasing condition of 60 s exposure at a 30 V bias.

The detrimental effect of substrate biasing is likely due to ion energies exceeding a material dependent damage threshold.^44,48^ Lin et al.^49^ found the damage threshold of Ar^+^ ions for a MoS_2_ monolayer to be between 25 and 35 eV. Lu et al.^50^ used 50 eV Ar^+^ ions to selectively remove the top S layer of bulk MoS_2_. Molecular dynamics simulations^51,52^ have suggested Ar^+^ ion sputtering thresholds of approximately 20 and 50 eV for S and Mo atoms in a MoS_2_ monolayer. Our experiments suggest that there is also a lower energy threshold below which Ar^+^ ions are ineffective in removing excess S. Plasma sources producing a high ion flux but low ion energies^33,53^ and tailored waveform biasing^45^ that can achieve accurate ion energy control are likely to be useful tools for the A B_0.20_ C_Ar_ and related processes.

### 2.3 Comparison of Material Properties

#### 2.3.1 Crystallinity, Microstructure, and Morphology

With the A B_0.20_ C_H2_, 10(A B_0.20_) C_H2_, and A B_0.20_ C_Ar_ processes optimized, we proceeded to compare these to each other along with a more detailed characterization of the crystallinity, morphology, impurities, and electrical properties. In addition to these three ABC processes developed in this work, we have included data for our previously developed A B_0.80_ process,^39^ which also deposits crystalline films at the same temperature of 150 °C. A table comparing the properties of the three ABC processes to each other as well as to different AB processes (A B_0.20_, A B_0.65_, and A B_0.80_) is provided in the Supporting Information (Section S3.4). This comparison highlights the improved control of the ABC approach, which significantly expands the range of film properties achieved compared with the preceding AB processes. Section S3.5 compares our processes and film characteristics to those in the literature. Finally, besides the selected three, other evaluated ABC processes including different plasma steps (H_2_S/Ar, H_2_, and Ar) added to the A B_0.80_ process are described in the Supporting Information (Section S5).

**Figure 6:**
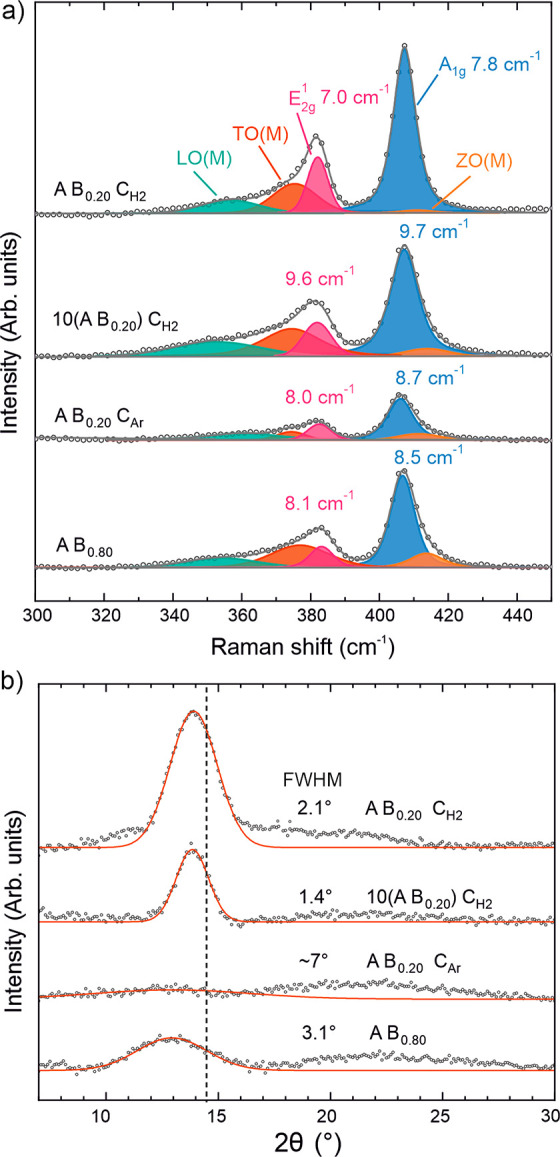
Crystallinity of ∼5 nm films deposited at 150 °C by using different ABC processes. (a) Raman spectra with peak deconvolution and fwhm’s for the E^1^_2g_ and A_1g_ peaks shown. (b) θ–2θ X-ray diffractograms after background removal showing also Gaussian peaks fitted to the (0002)MoS_2_ reflection and their fwhm’s. The dashed line shows the reference position (14.4° 2θ; JCDPS-ICDD powder diffraction file 00–037–1492). The broad peak centered at approximately 22° 2θ originates from the 450 nm thick SiO_2_ layer of the substrate. The data were offset vertically for clarity in both panels.

For a fair comparison of crystallinity, 5 nm thick films were deposited by using each of the processes. Deconvolution of Raman spectra in the region of the in-plane E^1^_2g_ and the out-of-plane A_1g_ MoS_2_ modes revealed clear differences between the processes (Figure 6a). The width of these modes is indicative of the crystalline quality: MoS_2_ monolayers exfoliated from high-quality bulk material yield sharp peaks (full-width at half-maximum, fwhm ≈3–4 cm^–1^), while the peaks broaden with increasing defectivity.^54^ For films of similar thickness, we have also observed a correlation of increased Raman intensity with improved crystallinity. Disorder-activated LO(M), TO(M), and ZO(M) modes provide another signature of defectivity, as the intensity ratio of these modes to E^1^_2g_ and/or A_1g_ increases with increasing defect density.^54^ All of these metrics suggest that the best crystalline quality was obtained using the A B_0.20_ C_H2_ process, which is also clearly improved over the A B_0.80_ process. The E^1^_2g_ fwhm of the A B_0.20_ C_H2_ process is comparable to or even lower compared to MoS_2_ deposited using other methods at 300–500 °C (Figure S29 in the Supporting Information). Applying H_2_ plasma only every 10 cycles (i.e., 10(A B_0.20_) C_H2_ process) resulted in clearly higher defectivity compared to that of the A B_0.20_ C_H2_ process. The A B_0.20_ C_Ar_ plasma process yielded fwhm’s between the two H_2_ plasma based processes but much lower peak intensities, which may be due to its smaller crystallite size discussed below.

Complementary information on crystallinity was obtained using X-ray diffraction (XRD) in symmetric θ-2θ geometry (Figure 6b) which probes crystalline planes parallel to the substrate surface. The (0002) reflection originating from MoS_2_ basal planes was observed for all of the processes at a similar position, slightly below that of a bulk reference. Only the A B_0.80_ process showed a clear shift to smaller angles, which suggested a larger spacing between the basal planes. This may be due to the large amount of hydrogen (∼20 at. %) incorporated.^39^

The intensity of the (0002) reflection, which can be taken as a measure of crystalline order (basal planes parallel to the substrate), was the highest for the A B_0.20_ C_H2_ process, followed by the 10(A B_0.20_) C_H2_ and A B_0.80_ processes. The peak intensity for the A B_0.20_ C_Ar_ process was very low. The width of the peak was the narrowest for the 10(A B_0.20_) C_H2_ process (fwhm = 1.4°); otherwise, it increased in the order of decreasing intensity. As peak broadening is caused by limited crystal size in the direction perpendicular to the substrate, which cannot exceed film thickness, the narrowest peak produced by the 10(A B_0.20_) C_H2_ process may be due to its smooth surface and underestimation of thickness by SE as discussed below with the TEM images.

**Figure 7:**
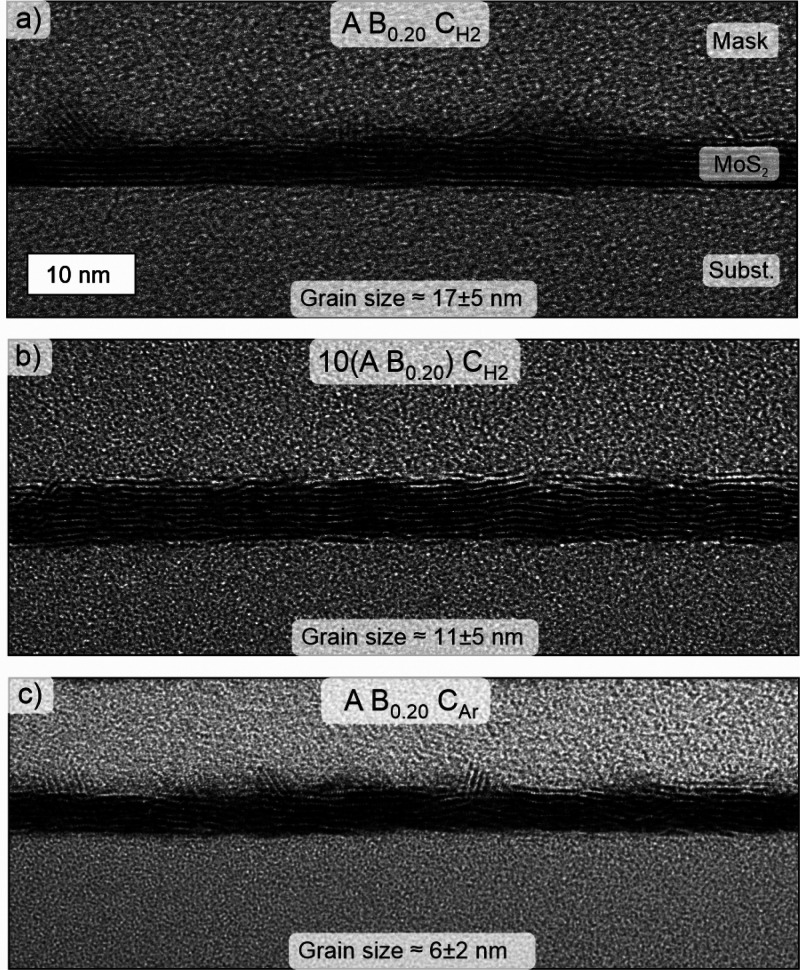
Microstructure of ∼5 nm MoS_2_ films deposited using (a) A B_0.20_ C_H2_, (b) 10(A B_0.20_) C_H2_, and (c) A B_0.20_ C_Ar_ processes as analyzed by cross-sectional TEM. Grain sizes estimated from multiple images are also indicated (average ± standard deviation).

Cross-sectional transmission electron microscopy (TEM) was used to complement Raman and XRD for crystallinity analysis (Figure 7, additional images in Figures S22–S24). The images show that for the A B_0.20_ C_H2_ process, the basal planes of the majority of the MoS_2_ crystallites were oriented approximately parallel to the substrate (Figure 7a). The shown film mainly consisted of 6–8 ML thick MoS_2_ crystallites resulting in a film thickness of 4.4 ± 0.5 nm (average of 12 locations), in good agreement with SE thickness (4.6 nm). In addition, some fins oriented at random angles were observed as is common for (PE)ALD TMDCs.^31,36,55,56^ The film grown by using the 10(A B_0.20_) C_H2_ process appeared more disordered, which may be due to its smaller crystallite size or a larger spread in the crystallite orientation (Figure 7b). Remarkably, the film appeared very smooth with no visible fins. The thickness measured from the TEM image, 5.9 ± 0.4 nm, was much larger than the 4.6 nm measured by SE. The highest disorder of the A B_0.20_ C_Ar_ films as suggested by Raman and XRD was also apparent in the TEM images. Nevertheless, the film appeared to be completely crystallized (Figure 7c). Some fins, albeit of a lower height compared to the A B_0.20_ C_H2_ process, are visible in Figure 7c. This shows that the 10(A B_0.20_) C_H2_ process prevents fins more efficiently than the A B_0.20_ C_Ar_ process. The TEM thickness of 4.6 ± 0.8 nm, agrees well with SE (4.8 nm). Thus, the SE models and extracted optical constants appear adequate for the A B_0.20_ C_H2_ and A B_0.20_ C_Ar_ processes (Figure S27), whereas for the 10(A B_0.20_) C_H2_ process, we were unable to develop an accurate model even with the TEM thickness information.

The lateral grain size was estimated by manually identifying crystallites from the TEM images, yielding 17 ± 5, 11 ± 5, and 6 ± 2 nm for the A B_0.20_ C_H2_, 10(A B_0.20_) C_H2_, and A B_0.20_ C_Ar_ processes (Figure 7). Although the absolute values carry some uncertainty due to the difficulty in unambiguously identifiying grain boundaries, the trend is in agreement with Raman, TEM, and XRD analysis. These grain sizes are comparable to MoS_2_ films deposited by ALD and CVD at 200–400 °C (Table S10). The crystallite orientation was quantified from the TEM images, confirming the lowest orientation spread with respect to the substrate surface for the A B_0.20_ C_H2_ process followed by the 10(A B_0.20_) C_H2_ and A B_0.20_ C_Ar_ processes (Figure S25).

**Figure 8:**
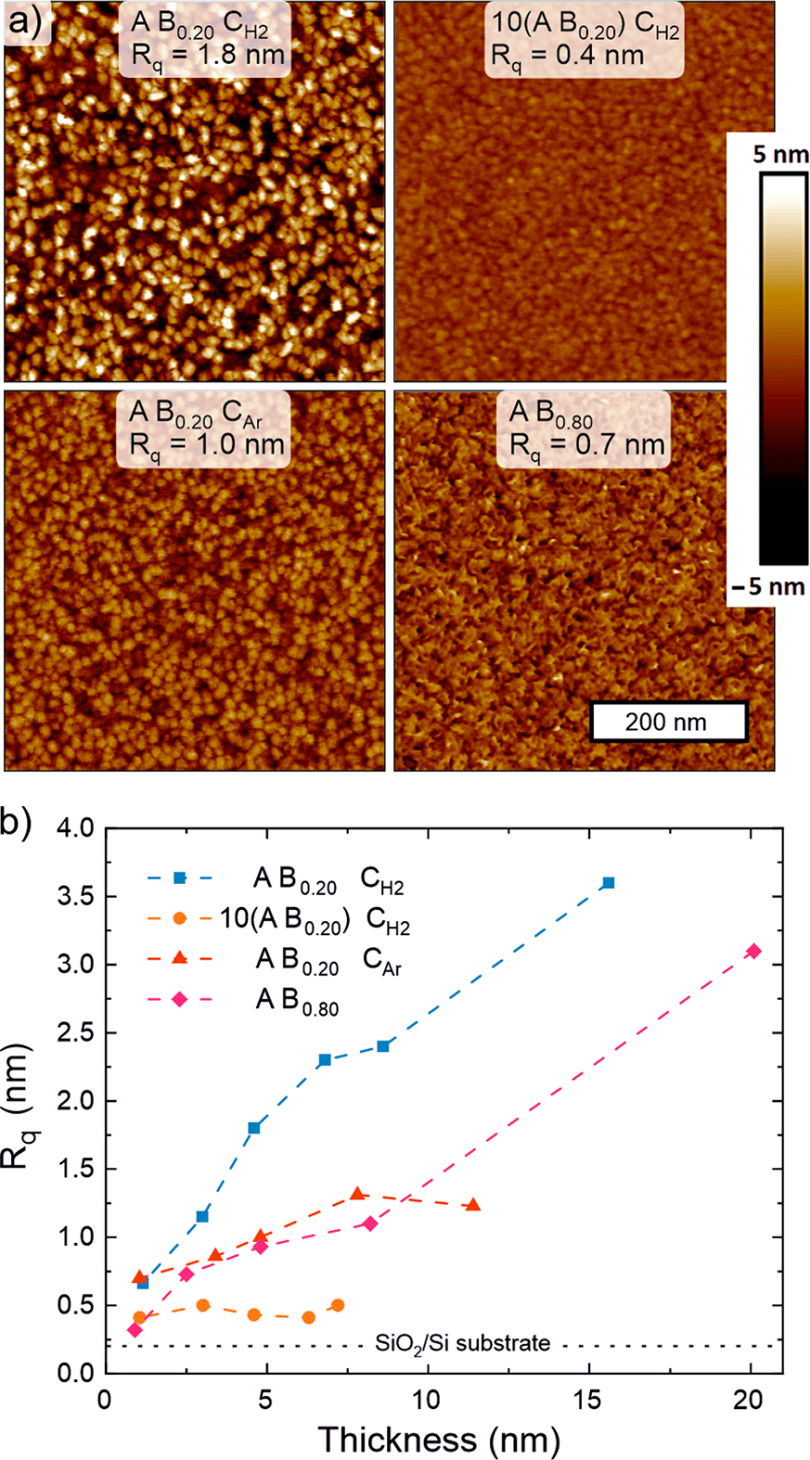
Morphology of MoS_2_ films deposited at 150 °C using different ABC processes (data for A B_0.80_ from ref (39) shown for reference). (a) AFM images of ∼5 nm thick films and (b) evolution of roughness as a function of thickness.

The morphology of the films deposited by using different processes was further studied by AFM. Figure 8a shows AFM images of ∼5 nm thick films. The already discussed drastic difference between the two H_2_ plasma based processes is clear: the A B_0.20_ C_H2_ process produces the roughest films out of all the studied processes, while the 10(A B_0.20_) C_H2_ process produces the smoothest films.

The evolution of morphology and roughness as a function of film thickness also greatly varies between the processes (Figure 8b–see Figure S26 for images). For the A B_0.20_ C_H2_ process, fins begin to form and grow already during the first cycles (i.e., below 1 nm thickness), resulting in a rapid and continuous increase of roughness with increasing thickness. In stark contrast, for the 10(A B_0.20_) C_H2_ process, surface roughness is independent of film thickness, showing that this process allows deposition of smooth (*R*_q_ ≈ 0.5 nm) films up to a thickness of at least 7 nm. The films deposited using the A B_0.20_ C_Ar_ process reach a moderate roughness (*R*_q_ ≈ 1.0 nm) with clearly visible surface features already at a thickness of 3 nm. However, increasing the thickness beyond 10 nm does not increase the roughness appreciably, despite an apparent lateral growth of the surface features. Thus, although comparable to the A B_0.80_ process at a thickness of ≤7 nm, at higher thicknesses the A B_0.20_ C_Ar_ process produces smoother films. Linking this result to the TEM images suggests that fins form for the Ar plasma based process, but they do not easily grow as they do for the A B_0.20_ C_H2_ and A B_0.80_ processes (Figure 3).

#### 2.3.2 Film Composition

The S/Mo stoichiometry and chemical environment of Mo and S were analyzed by using core-level X-ray photoelectron spectra. As discussed in detail with the optimization of each process, the H_2_ plasma based processes deposit practically stoichiometric MoS_2_, while the Ar plasma is less efficient in removing excess S and results in slightly overstoichiometric films (MoS_2.3_).

**Figure 9:**
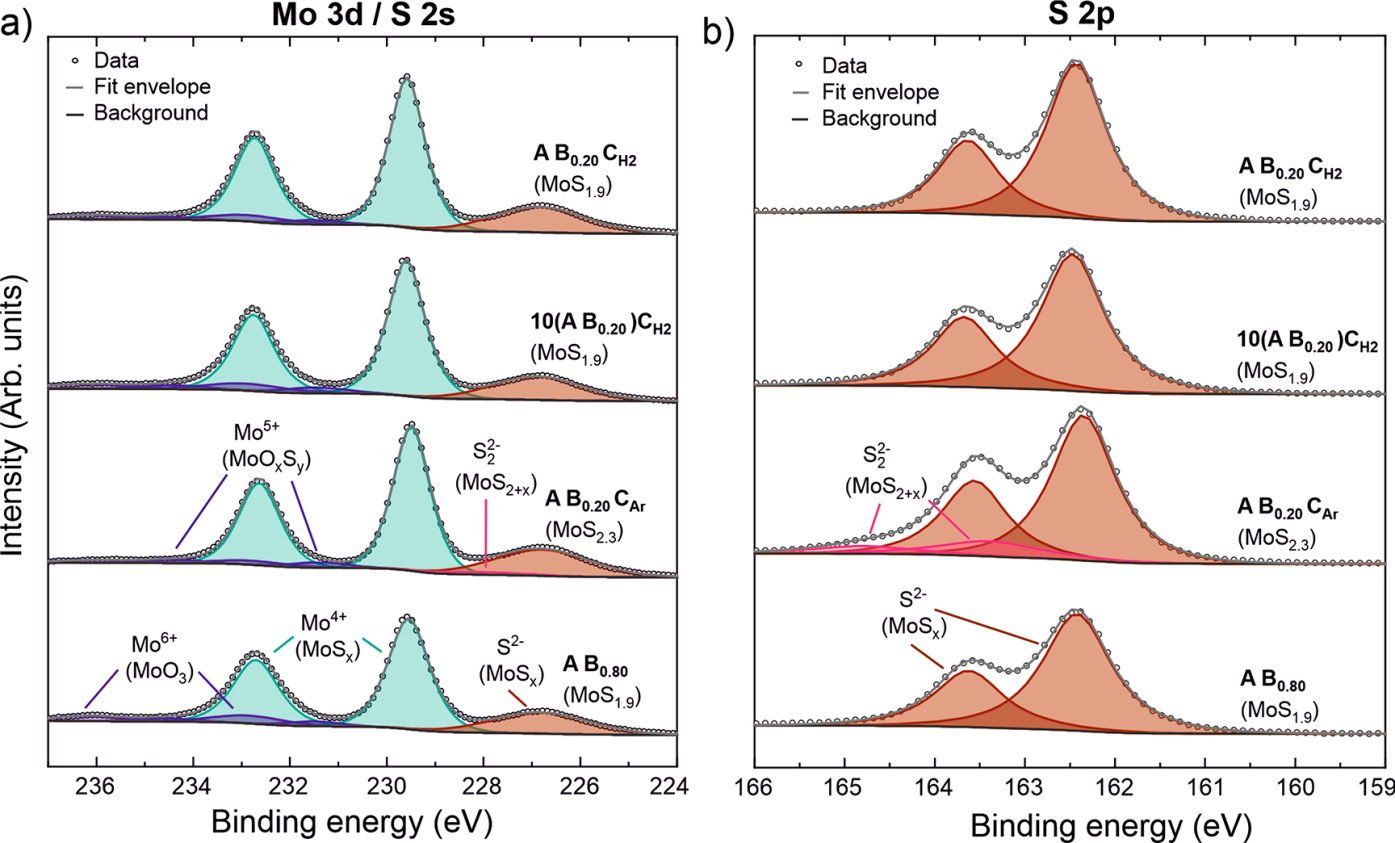
XPS spectra of (a) Mo 3d/S 2s and (b) S 2p core levels of MoS_2_ films deposited by using different ABC processes and 140 ALD cycles at 150 °C. S/Mo stoichiometry is indicated, and the A B_0.80_ process is shown for comparison. The spectra were vertically offset for clarity.

Using the A B_0.20_ C_H2_ process as an example, the Mo 3d region mainly consisted of a doublet attributed to Mo^4+^ in MoS_2_ with a Mo 3d_5/2_ binding energy (BE) of 229.6 eV (Figure 9a, see Table S7 in the Supporting Information for more details of the peak deconvolution). Minor doublets attributed to Mo^5+^ and Mo^6+^ species at Mo 3d_5/2_ BEs of 231.5 and 232.9 eV are likely a result of oxidation of the films in air.^57−59^ The observed BEs agree well with average literature values of 229.3 ± 0.5 eV for MoS_2_ and 232.7 ± 0.3 eV for MoO_3_.^60^ The Mo 3d region was very similar for all of the ALD processes.

S 2s region overlaps with Mo 3d, and can be described with one singlet attributed to S^2–^ in MoS_2_ (BE = 226.8 eV). For the A B_0.20_ C_Ar_ process, a minor additional contribution at approximately 228 eV was observed. The S 2p region was analyzed for further information about the S species present (Figure 9b). For the A B_0.20_ C_H2_ process, this could be fit well with a doublet attributed to S^2–^ in MoS_2_, the S 2p_3/2_ BE of 162.4 eV being in good agreement with literature (BE = 162.3 ± 0.6 eV).^60^ The measured S 2p spectrum was practically identical for the A B_0.20_ C_H2_, 10(A B_0.20_) C_H2_, and A B_0.80_ processes, which also share a similar stoichiometry. For the more sulfur rich MoS_2.3_ films deposited using the A B_0.20_ C_Ar_ process, a low intensity doublet at a higher BE (163.4 eV for S 2p_3/2_) was observed, which is attributed mainly to the S_2_^2–^ species present in sulfur-rich amorphous MoS_2+*x*_. Amorphous MoS_2+*x*_ contains sulfur in many different bonding environments with partially overlapping BEs as discussed in the literature,^61,62^ making unambiguous assignment to different S species challenging.

The film stoichiometry was confirmed by Rutherford backscattering spectrometry (RBS) measurements, which were in good agreement with the XPS (Table 1). RBS together with elastic recoil detection analysis (ERD) was also used to analyze film impurities. The A B_0.20_ and A B_0.80_ processes are shown for reference along with the analyzed A B_0.20_ C_H2_ and A B_0.20_ C_Ar_ process. Although the 10(A B_0.20_) C_H2_ process was not analyzed, we expect it to result in a composition resembling the A B_0.20_ C_H2_ process.

**Table 1:** Elemental Composition of ∼50 nm Thick MoS_*x*_ Films Deposited by Using Different Processes^a^

Process	S/Mo (RBS)	S/Mo (XPS)	H (at. %)	C (at. %)	N (at. %)	O (at. %)	Ar (at. %)
A B_0.20_	3.70 ± 0.13	3.54 ± 0.04	6.3 ± 0.5	<dl	0.5 ± 0.2	0.8 ± 0.2	<dl
A B_0.20_ C_H2_	1.95 ± 0.07	1.89 ± 0.02	9.0 ± 0.7	<dl	0.7 ± 0.2	1.4 ± 0.4	<dl
A B_0.20_ C_Ar_	2.30 ± 0.08	2.32 ± 0.02	2.3 ± 0.2	<dl	1.4 ± 0.4	1.2 ± 0.4	0.18 ± 0.05
A B_0.80_	1.83 ± 0.07	1.89 ± 0.02	22 ± 2	<dl	1.0 ± 0.3	6 ± 2	0.07 ± 0.06

a Composition was analyzed by RBS (Mo, S, C, N, O, Ar) and ERD (H). Detection limits (dl) are approximately 7 and 0.05 at. % for C and Ar, respectively. S/Mo ratios determined by XPS are shown for comparison. The RBS and ERD uncertainties comprise known systematic and statistical uncertainties, while the XPS uncertainty represents statistical uncertainty only. The data for the A B_0.20_ and A B_0.80_ processes has been published in ref (39).

The nitrogen content from incorporated precursor ligands was low for all processes, 0.5–1.4 at. %. Oxygen was observed in low concentrations for the processes using a B_0.20_ step, 0.8–1.4 at. %, while clearly more oxygen was detected for the A B_0.80_ process (6 at. %). As O may result from postdeposition oxidation in air or residual impurities in the ALD reactor, this suggests higher resistance to oxidation for the films deposited using the ABC processes, which may be linked to their lower H concentration. Up to 22 at. % H was incorporated using high H_2_ flow ratios in B step (A B_0.80_). The processes based on a B_0.20_ step incorporated less H. Approximately 6 at. % of H was found in the amorphous sulfur rich films deposited by the A B_0.20_ process, which increased modestly to 9 at. % when a H_2_ plasma C step was added (A B_0.20_ C_H2_). Using an Ar plasma C step instead (A B_0.20_ C_Ar_) decreased the H content to as little as 2.3 at. %. As our previous work identified H as a dopant, decreasing the H content in the films gives promise for better semiconductor properties, namely, lower carrier densities and higher mobilities.

#### 2.3.3 Electrical Properties

Motivated by the decrease of the H content in the films prepared by the newly developed ABC processes, we investigated their electrical properties and compared them to the previously developed A B_0.80_ process, which yields the best electrical performance out of low-temperature (<200 °C) AB processes.^39^ The ABC processes resulted in considerably higher four-point-probe (FPP) resistivities compared to the A B_0.80_ process (∼0.3 Ω cm), ranging from approximately 10 Ω cm for both of the H_2_ plasma processes to ∼200 Ω cm for the A B_0.20_ C_Ar_ process. These resistivity values were reached for films at least 5–7 nm in thickness. Thinner films exhibited higher resistivities, likely due to increased scattering of charge carriers at the interfaces as well as at grain boundaries (Figure S28).

AC Hall effect measurements were performed to understand the origin of the resistivity changes. Carrier densities of both of the H_2_ plasma based processes were found to be 3 orders of magnitude lower compared to the A B_0.80_ process, i.e., in the range of ∼1–3 × 10^18^ cm^–3^ (see Section S3.3 in the Supporting Information for detailed results and further discussion). Using the A B_0.20_ C_Ar_ process, a further decrease in carrier density to ∼6 × 10^16^ cm^–3^ was achieved. The carrier densities decrease with decreasing hydrogen concentration, in line with hydrogen being a dopant in the films.^39^ The relationship is not linear, however, suggesting that other factors including chemical bonding of H, other impurities (O, N), crystallinity, and morphology may also affect the electrical properties (Section S3.3).

Furthermore, the Hall mobility of the three ABC processes was an order of magnitude higher compared to the A B_0.80_ process, that is, approximately 0.3 cm^2^ V^–1^ s^–1^. The improved mobility may be due to decreased charge carrier scattering as a result of both a lower carrier concentration and improved crystallinity compared to the A B_0.80_ process. The decreased carrier densities and increased mobilities over previous work give promise for semiconductor applications, especially plastic-based flexible electronics requiring a very low thermal budget. The smooth morphology of the 10(A B_0.20_) C_H2_ process appears to be particularly suitable for electronics.

### 2.4 Application in Electrocatalysis

**Figure 10:**
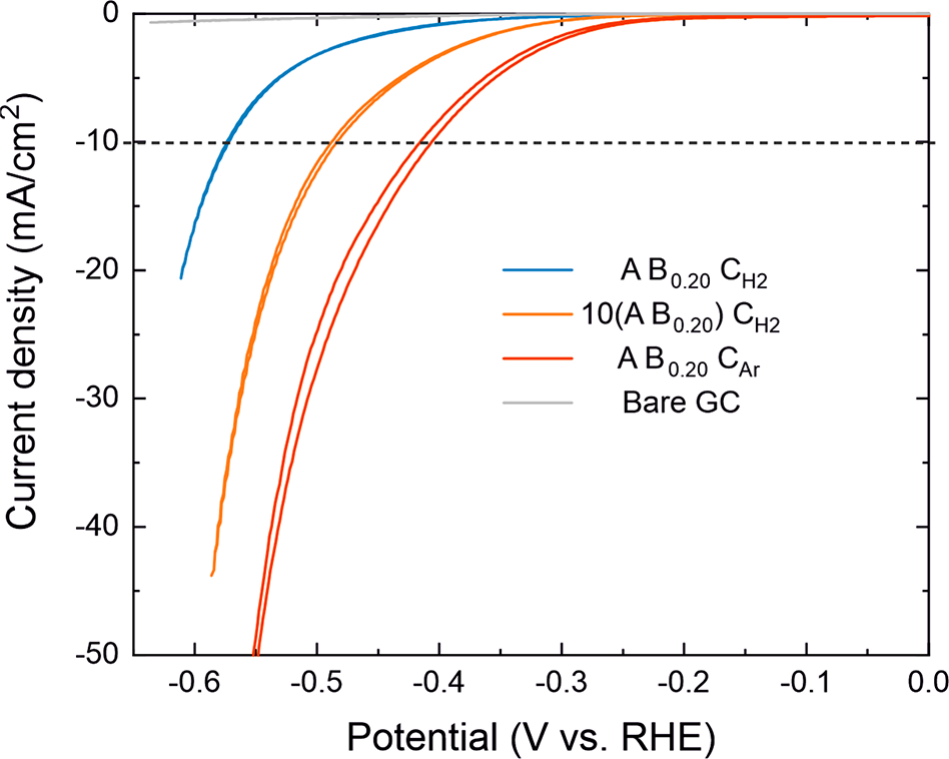
CVs of MoS_2_ films deposited on GC and the bare substrate used as a reference. The fifth CV scan recorded for each sample is shown.

Electrocatalytic HER is a commonly explored TMDC application, which benefits from the presence of defects such as crystallite edges and vacancies that have been identified as catalytically active sites.^12,14,15^ Here, we deposited approximately 7 nm thick MoS_2_ films on glassy carbon (GC) substrates by using our three ABC processes. Figure 10 shows cyclic voltammograms (CVs) of the different catalysts as well as the bare GC substrate recorded in 0.5 M H_2_SO_4_. The most commonly used activity metric is the overpotential η required to reach a current density of 10 mA/cm^2^, which we denote η_10_.^63,64^ As the thermodynamic potential for HER is 0 V versus RHE, this is equal to the absolute value of the (negative) potential applied at this current density. The lowest η_10_ value of 406 mV was observed for the A B_0.20_ C_Ar_ process followed by the 10(A B_0.20_) C_H2_ (484 mV) and A B_0.20_ C_H2_ (573 mV) processes (Figure 10 and Table 2). The HER currents measured for all of the samples were considerably higher compared with the bare substrate, confirming that our MoS_2_ films are indeed catalytically active. Tafel analysis provides a complementary view to HER activity, as a low Tafel slope indicates that high current densities that are required for practical electrolyzers can be achieved at reasonable overpotentials.^16^ The Tafel slopes (Table 2 and Figure S31) of the 10(A B_0.20_) C_H2_ (115 mV/dec) and A B_0.20_ C_Ar_ (123 mV/dec) processes were lower compared to those of the A B_0.20_ C_H2_ process (165 mV/dec), which also required the highest overpotential.

**Table 2:** Summary of HER Catalyst Metrics Extracted from Electrochemical Measurements

Process	η_10_ (mV)	Tafel slope (mV/dec)	*C*_dl_ (μF/cm^2^)
A B_0.20_ C_H2_	573	165	18
10(A B_0.20_) C_H2_	484	115	36
A B_0.20_ C_Ar_	406	123	135

To rationalize the differences in activity between the different MoS_2_ catalysts, double layer capacitance (C_dl_) of the films was measured by scanning potential in the non-Faradaic region at varying scan rates (see Table 2 and Figure S33 for the measured data and fits). The measured *C*_dl_ values increased in the order of increasing HER activity (decreasing η_10_). *C*_dl_ is proportional to the density of double layer adsorption sites, which appears to correlate here with the density of HER active sites. Indeed, normalizing the current densities by *C*_dl_ brought the activities of different samples closer to each other (Figure S32 in the Supporting Information).

The lowest *C*_dl_ produced by the A B_0.20_ C_H2_ process may result from its largest grain size and orientation of most of the MoS_2_ crystallites parallel to the substrate (Figure 7a). The fins, although resulting in a large roughness, only provide a limited amount of active edge sites due to their relatively low surface coverage, in agreement with previous studies on PEALD WS_2_.^37^ Although smoother on the surface, the 10(A B_0.20_) C_H2_ films may provide a higher accessible edge site density due to a smaller grain size and larger spread in the crystallite orientation. In addition, the more disordered structure suggested by Raman and TEM may indicate an increased number of active (defect) sites on the basal planes. Combining the clearly highest *C*_dl_ together with the lowest η_10_ as well as the S-rich composition suggests that the A B_0.20_ C_Ar_ films contain disordered c-MoS_2_ domains and potentially even active-site-rich nanoscale a-MoS_2+*x*_ clusters.^65,66^ This makes the Ar plasma based process the preferred choice for HER.

The overpotentials and Tafel slopes of our three catalysts fall in the range reported for nanocrystalline, relatively smooth MoS_2_ thin films on flat, inert substrates.^31,67,68^ This greatly exceeds the activity of bulk MoS_2_ (η_10_ > 1000 mV),^68^ but falls short of the best MoS_2_ catalysts taking advantage of, for example, vacancy engineering and strain, which can achieve η_10_ approaching 150 mV (refs (13, 15, and 69) and references therein). As shown by the activity differences among our three processes, we envision that ABC type PEALD processes can be optimized for defect engineering by rationally controlling different plasma species. Depositing MoS_2_ on high-surface area substrates, which is one of the unique strengths of ALD, can be used to improve performance toward practical catalysts. Doping is another strategy yielding improvements in the HER performance of MoS_2_,^14,15^ which can be implemented in (PE)ALD to improve catalytic activity.

## 3 Conclusions

We developed a toolbox of advanced PEALD processes depositing wafer-scale polycrystalline MoS_2_ films with rationally controlled properties and thickness at 150 °C, one of the lowest temperatures achieved in the literature. Using two individually tailored plasma exposures in an “ABC cycle” greatly improves the control over film properties compared to conventional “AB” type PEALD processes with a single plasma exposure per cycle. Our approach gives ample flexibility in process design via control of plasma parameters such as plasma gases, pressure, power, exposure time, and order and periodicity of different plasma steps. We have focused on three processes, where H_2_ or Ar plasma exposure is used to remove excess sulfur incorporated into the films from H_2_S plasma. The choice of either Ar or H_2_ has a strong effect on film properties as summarized in Table 3: while H_2_ plasma yields more crystalline films, these films are usually rough and contain approximately 10 at. % H. Intriguingly, applying the H_2_ plasma step only every 10 cycles results in smooth, crystalline films. We explain this to be a result of growth of smooth a-MoS_2+*x*_ followed by periodic removal of excess S. Using Ar plasma yields more disordered films compared to H_2_ plasma but also alleviates H incorporation to 2 at. % and consequently decreases the level of doping. Compared to AB processes, we can substantially decrease the carrier density of our MoS_2_ from 10^21^ to 10^16^–10^18^ cm^–3^ along with an at least order of magnitude increase in Hall mobility up to 0.3 cm^2^ V^–1^ s^–1^. These results combined with the plastic-compatible deposition temperatures make our processes interesting for flexible electronics. The smooth morphology resulting from H_2_ plasma applied every 10 cycles (10(A B_0.20_) C_H2_) appears particularly suitable for (opto)electronics. The control of morphology and exposure of fins and grain boundaries achieved by different plasmas is of interest for flexible gas sensors. The different ABC processes exhibit different levels of activity for electrocatalytic HER, which can be linked to the film morphology and crystallinity. The Ar plasma process displays the highest HER activity stemming from active sites, such as crystallite edges and other defects. The flexibility, scalability, and low thermal budget of our approach gives ample opportunities for further expanding the ABC process toolbox to tailor MoS_2_ and other TMDCs for electronics, catalysis, and other applications.

**Table 3:** Summary of Selected Film Characteristics of the Three ABC Processes

	A B_0.20_ C_H2_	10(A B_0.20_) C_H2_	A B_0.20_ C_Ar_
*Composition:*			
S/Mo ratio^a^	1.9	1.9	2.3
Impurities (at. %)^b^	H: 9, O: 1.4, N: 0.7		H: 2.3, O: 1.2, N: 1.4, Ar: 0.2
*Film growth:*			
GPC (Å)^c^	1.1	0.4	0.8
Nonuniformity (4 in.)^c^	2.4%		2.2%
*Structure:*			
Crystallinity^d^	Highest	Medium	Lowest
Grain size (nm)^e^	17 ± 5	11 ± 5	6 ± 2
Roughness (nm)^f^	2.3	0.5	1.3
*Electrical properties*^g^			
*n* (cm^–3^)	1.3 ± 0.7 × 10^18^	2.6 ± 1.4 × 10^18^	6 ± 3 × 10^16^
μ (cm^2^ V^–1^ s^–1^)	0.25 ± 0.15	0.30 ± 0.13	0.36 ± 0.17
*HER performance*			
*η*_10_ (mV)	573	484	406

a XPS and RBS.

b RBS and ERD.

c SE, averaged over 140 cycles.

d Raman, XRD, and TEM.

e TEM.

f AFM, 7 nm thickness.

g AC Hall effect.

## 4 Experimental Section

### 4.1 Film Deposition

MoS_*x*_ thin films were deposited by PEALD using an Oxford Instruments FlexAL PEALD reactor equipped with a remote 13.56 MHz inductively coupled plasma (ICP) source. The chamber was pumped with a turbomolecular pump capable of reaching a base pressure of 1 × 10^–6^ Torr. Unless otherwise noted, the table and wall temperatures were set to 150 °C, meaning that the reactor acted as a hot wall reactor with a substrate temperature of 150 °C. For experiments at higher temperatures, only the table temperature was increased, while for lower temperatures both the table and wall temperatures were set to the indicated temperature. The films were deposited on silicon(100) substrates with a 450 nm wet thermal SiO_2_ layer on top (Siegert Wafer). In most cases, approximately 1 × 1 to 3 × 3 cm^2^ coupons were used on an 8 in. carrier wafer that was introduced into the chamber through a load lock. After transfer, the substrates were let to thermally equilibrate for 5 min before starting the deposition.

ABC type PEALD processes were mostly used in this work. The A step consisted of 6 s of Mo(N^t^Bu)_2_(NMe_2_)_2_ dosing at 200 mTorr, the B step of 20 s H_2_S/H_2_/Ar plasma exposure at 6 mTorr, and the C step of an additional Ar or H_2_ plasma exposure (conditions indicated in Table 4). In addition, a purging/pumping step was included after every A, B, and C step. After an A step, both a 6 s purging step with 300 sccm Ar flow and 4 s pumping without gas flows were used, while after B and C steps only the 6 s Ar purging step was employed. Additional, 4 and 5 s long pressure and gas flow stabilization steps preceded the Mo precursor and plasma pulses, respectively. Figure S1 in the Supporting Information shows the pulsing schemes used for different processes.

**Table 4:** Plasma Conditions Used for Different Processes in the B and C steps.^a^

Plasma step	Exposure time (s)	H_2_S flow rate (sccm)	H_2_ flow rate (sccm)	Ar flow rate (sccm)	p (mTorr)	ICP P (W)
B_0.20_	20	8	2	40	6	100
B_0.80_	20	2	8	40	6	100
C_H2_	20 (5–60)	0	100	0	20 (20–300)	100
C_Ar_	60 (20–120)	0	0	10 (10–40)	1 (1–6)	100 (100–500)

a The first number represents optimized conditions for each process, while the range of parameters evaluated is indicated in parentheses.

The processes are denoted A B_*x*_ C_*y*_ where *x* represents the H_2_ flow ratio in the B step according to eq 1 and y represents the plasma feed gas(es) for the C step. In most of the processes, a C step was added to each ALD cycle. Adding a C step only every *n* cycles instead of every cycle, is denoted *n*(A B_*x*_) C_*y*_. Unless otherwise noted, both the B and C steps are plasma steps.

The Mo(N^t^Bu)_2_(NMe_2_)_2_ (98%, Strem Chemicals) precursor was heated to 50 °C in an external canister and supplied to the chamber by Ar flow through delivery lines that were heated to 70 °C to prevent condensation. The flow rates of H_2_S (99.5%), H_2_ (99.999%), and Ar (99.999%) gases supplied by Linde gas were controlled by mass flow controllers. The pressure was controlled either by gas flows (1–20 mTorr) or an automatic butterfly valve (36–300 mTorr).

Experiments using RF substrate biasing^44,47,48^ were performed to increase energy of Ar^+^ ions impinging on the surface as discussed in the Supporting Information (Sections S2.5, S2.6).

### 4.2 Film Characterization

Film thicknesses were measured by spectroscopic ellipsometry (SE). During most of the depositions, the reactor was equipped with an *in situ* SE, either M-2000FI (spectral range of 0.7–5.0 eV) or M-2000F (spectral range of 1.2–5.0 eV) by J.A. Woollam. Typically, film thickness was measured every 10 cycles. For evaluation of growth mechanisms, measurements were performed after each B and C step. For depositions without an *in situ* SE, film thicknesses were measured *ex situ* after deposition using a variable angle J.A. Woollam M-2000U instrument (spectral range of 1.2–6.5 eV, angles 65, 75, and 85°). The SE modeling procedure was described in our previous work.^39^ A B-spline model with 0.3 eV resolution was used to describe the MoS_2_ films.

*Ex situ* uniformity mapping was performed using a J.A. Woollam M-2000 instrument (spectral range 1.2–3.3 eV) equipped with an automated mapping stage. A circular pattern excluding areas close to the wafer edges was used.

Film morphology was examined by scanning electron microscopy (SEM, Zeiss Sigma) using an InLens detector and acceleration voltage of 3 kV. Additionally, morphology and surface roughness were analyzed by atomic force microscopy (AFM, Bruker Dimension Icon) in PeakForce Tapping based ScanAsyst mode in air. Probes with a nominal spring constant of 0.4 N/m and a tip radius of 2 nm were used (ScanAsyst-Air, Bruker). Surface roughness was calculated as root-mean-square (*R*_q_) value from 500 × 500 nm images after first order flattening using Bruker Nanoscope 2.0 software.

Film crystallinity was evaluated by Raman spectroscopy (Renishaw inVia confocal Raman microscope) equipped with a 514 nm laser, 50× objective (NA = 0.75), and 1800 lines/mm grating. Six spectra using 10 s accumulation time each were collected for each sample. Incident laser power was estimated to be 0.6 mW on the sample (100 mW laser with a 1% neutral density filter, accounting for optical losses). All of the presented spectra were normalized to the intensity (area) of the Si mode at 520 cm^–1^. For deconvolution of the A_1g_/E^1^_2g_ region, five Pseudo-Voigt peaks were fitted in the 300–450 cm^–1^ range. X-ray diffraction (XRD) measurements complementing the Raman analysis were performed in symmetric θ–2θ geometry using a Bruker D8 Discover diffractometer equipped with a Cu X-ray tube (Kα λ = 1.54 Å). Background was removed by fitting a cubic spline function to the data.

Film composition was analyzed by X-ray photoelectron spectroscopy (XPS) using a Thermo Scientific K-Alpha spectrometer equipped with a monochromatic Al Kα source (*h*ν = 1486.6 eV) focused to a 400 μm spot on the sample. An electron flood gun was used during the measurements to minimize the charging effects. The binding energies were referenced by setting the adventitious carbon derived C–C/C-H component of the deconvoluted C 1s peak to 284.8 eV. A pass energy of 20 eV was used for high-resolution spectra. Peak deconvolution was performed using Avantage software (Thermo Scientific) using Gaussian–Lorentzian (30% Lorentzian) sum functions with a Shirley-type background.

Rutherford backscattering spectrometry (RBS) and elastic recoil detection (ERD) measurements were performed by Detect 99 B.V. (Eindhoven, The Netherlands) using a 2 MeV He^+^ beam. RBS was used to determine the absolute areal atom density, S/Mo stoichiometry, and impurities besides hydrogen. Hydrogen concentration was determined by ERD. The reported uncertainties take into account known systematic and statistical uncertainties.

Transmission electron microscopy (TEM) imaging was performed using a probe-corrected JEOL ARM 200F instrument operated at 200 kV. The cross-sectional TEM samples were prepared by FEI Helios Nanolab 600 or 600i dual-beam focused-ion beam FIB/SEM instruments following a standard lift-out procedure with a SiO_2_ or C/Pt mask layer.

Sheet resistance of the films was determined as the slope of an *I*–*V* curve measured by a four-point probe (FPP, Signatone SP4–40045TRS probe head connected to a Keithley 2400 SourceMeter). Film resistivity was calculated by multiplying the sheet resistance by the film thickness measured by SE. To extract carrier concentration and mobility, AC Hall effect measurements were performed in van der Pauw geometry on approximately 1 × 1 cm^2^ samples using a Lakeshore 8404 HMS instrument.

### 4.3 Electrocatalysis

Electrochemical measurements were performed using a three-electrode setup controlled by an Autolab PGSTAT302N potentiostat (Metrohm) and a custom-made glass cell. Working electrodes were constructed by depositing 7 nm thick MoS_2_ thin films on glassy carbon (GC) plates (2.2 × 2.2 × 0.3 cm^2^, SIGRADUR G, HTW Hochtemperatur-Werkstoffe GmbH). Prior to deposition, the GC plates were polished by using an aqueous dispersion of 50 nm Al_2_O_3_ nanoparticles (BASi) applied to a velvet polishing pad (BASi) followed by rinsing with deionized water. The polishing procedure results in *R*_q_ ≈ 1–3 nm, which is similar to that of MoS_2_ films. No signs of plasma damage or etching of the GC were observed after deposition.

The MoS_2_/GC samples were mounted to a custom-made poly(ether ether ketone) (PEEK) sample holder exposing a 3.14 cm^2^ area of the sample. The holder was connected to a rotating disk electrode (RDE) assembly. A graphite rod counter electrode (diameter 6.3 mm, 99%, metal basis, Thermo Scientific) was positioned in a separate compartment separated by a glass frit. A commercial saturated calomel electrode (SCE, CH instruments) reference electrode was located in another glass-frit separated compartment. A Luggin capillary was used to position the reference electrode close (∼0.5 cm) to the working electrode.

Prior to use in the catalytic measurements, the SCE was externally referenced to ferrocenecarboxylic acid in 0.2 M phosphate buffer titrated with NaOH to pH 7 (284 mV vs SCE).^70^ The reference potential determined by this method was then used to convert the measured potentials to a reversible hydrogen electrode (RHE) scale.

The cell was filled with ∼100 mL of 0.5 M H_2_SO_4_ electrolyte (pH 0.3) prepared from concentrated H_2_SO_4_ (Fischer Chemical Optima) and ultrapure water (resistivity 18.2 MΩcm). The electrolyte was purged by bubbling N_2_ for at least 30 min prior to measurements. During measurements, the gas flow was switched to purge the headspace above the electrolyte.

Measurement procedure began with an electrochemical impedance spectroscopy (EIS) measurement (−0.2 V vs SCE, 10 Hz to 100 kHz, 10 mV root-mean-square AC amplitude). The impedance of the data point with phase closest to 0 (typically at 50–100 kHz) was taken as the uncompensated resistance (typically 0.3–0.4 Ω) and was used for 100% *iR* postmeasurement compensation of the measured CVs. Then, a set of CV scans were performed in an inert potential window (−0.2 to 0.4 V vs SCE) to estimate the initial double layer capacitance (C_dl_). In these measurements, the scan rate was increased from 50 to 250 mV/s in 50 mV/s increments with three scans recorded per scan rate. Next, five cyclic voltammetry (CV) scans in the HER region (−0.2 to −0.9 V and back vs SCE) were recorded at a scan rate of 10 mV/s while the electrode was rotated at 1600 rpm. The data from the fifth scan were used to extract the overpotentials and Tafel slopes (reported after *iR* compensation). Following these scans, the rotation was stopped, and visible bubbles (if any) physically removed with a glass pipet, followed by a second set of CV scans in inert region (identical to above) to estimate *C*_dl_. This latter set of scans was used for determining the presented *C*_dl_ values, as these were taken to better correspond to the sample state during HER compared to the measurements performed before HER. *C*_dl_ was determined as the slope of the line fit to current densities of anodic scans at 0.4 V vs RHE at different scan rates.

## Supporting Information Available

Schematic of the optimized PEALD cycles, demonstration of thickness control and uniformity; optimization and additional characterization (AFM, FPP, Raman, SE, SEM, TEM, XPS, XRD) of the ABC processes discussed in the article (A B_0.20_ C_H2_, *n*(A B_0.20_) C_H2_, and A B_0.20_ C_Ar_) as well as other tested processes (A B_0.20_ C_H2/Ar_, A B_0.80_ C_H2S/Ar_, A B_0.80_ C_H2_, and A B_0.80_ C_Ar_); discussion on substrate biasing and effect of ions on film growth; additional process and literature comparison, electrical characterization, and electrocatalysis data and discussion (PDF)
