# Supplementary material for: Toolbox of Advanced Atomic Layer Deposition Processes for Tailoring Large-Area MoS2 Thin Films at 150 °C
Source: ACS Appl Mater Interfaces. 2023 Jul 17;15(29):35565–79. doi: 10.1021/acsami.3c02466 (PMC10375433; doi:10.1021/acsami.3c02466)
Supplement: Supplementary file 1 — am3c02466_si_001.pdf [file am3c02466_si_001.pdf]

Supporting information for: Toolbox of Advanced Atomic Layer Deposition Processes for Tailoring Large-Area MoS_2 Thin Films at 150 °C

Miika Mattinen,[1†] Jeff J. P. M. Schulpen,[1] Rebecca A. Dawley,[2] Farzan Gity,[3] Marcel A. Verheijen,[1,4] Wilhelmus M. M. Kessels,[1] Ageeth A. Bol*[1,2]

1 Department of Applied Physics and Science Education, Eindhoven University of Technology, PO Box 513, 5600 MB Eindhoven, The Netherlands
2 Department of Chemistry, University of Michigan, 930 N. University Ave, Ann Arbor, MI, 48109-1055, United States of America
3 Tyndall National Institute, University College Cork, Lee Maltings, Dyke Parade, Cork T12 R5CP, Ireland
4 Eurofins Materials Science Netherlands, High Tech Campus 11, 5656 AE Eindhoven, The Netherlands

*corresponding: a.a.bol@tue.nl †Present address: Department of Chemistry, University of Helsinki

Contents

S1: Concept of developed ABC processes S2
S1.1: Schematics of optimized ALD processes S2
S1.2: Accurate thickness control and uniformity S3
S2: Optimization of the three ABC processes discussed in the main text S4
S2.1: A $B_{0.20}$ C_{H2} process optimization S4
S2.2: A $B_{0.20}$ C_{H2} process at different temperatures S7
S2.3: n(A $B_{0.20}$) C_{H2} process: effect of H_2 plasma frequency n on film growth and properties S8
S2.4: A $B_{0.20}$ C_{Ar} process optimization S13
S2.5: Substrate biasing in A $B_{0.20}$ $C_{Ar\ bias}$ process S16
S2.6: Ion energy and flux estimates based on literature S20
S3: Comparison of material properties S22
S3.1: Crystallinity, microstructure, and morphology S22
S3.2: Film composition S27
S3.3: Electrical properties S28
S3.4 Summary S30
S3.5 Literature comparison S31
S4: Application in HER electrocatalysis S33
S5: Experiments on additional ABC processes S36
S5.1: A $B_{0.20}$ $C_{H2/Ar}$ process using mixed H_2/Ar plasma S35
S5.2: A $B_{0.80}$ $C_{H2S/Ar}$ process S37
S5.3: Trials of A $B_{0.80}$ C_{H2} process S40
S5.4: Trials of A $B_{0.80}$ C_{Ar} process S41
S6: References S42

S1: Concept of developed ABC processes

S1.1: Schematics of optimized ALD processes

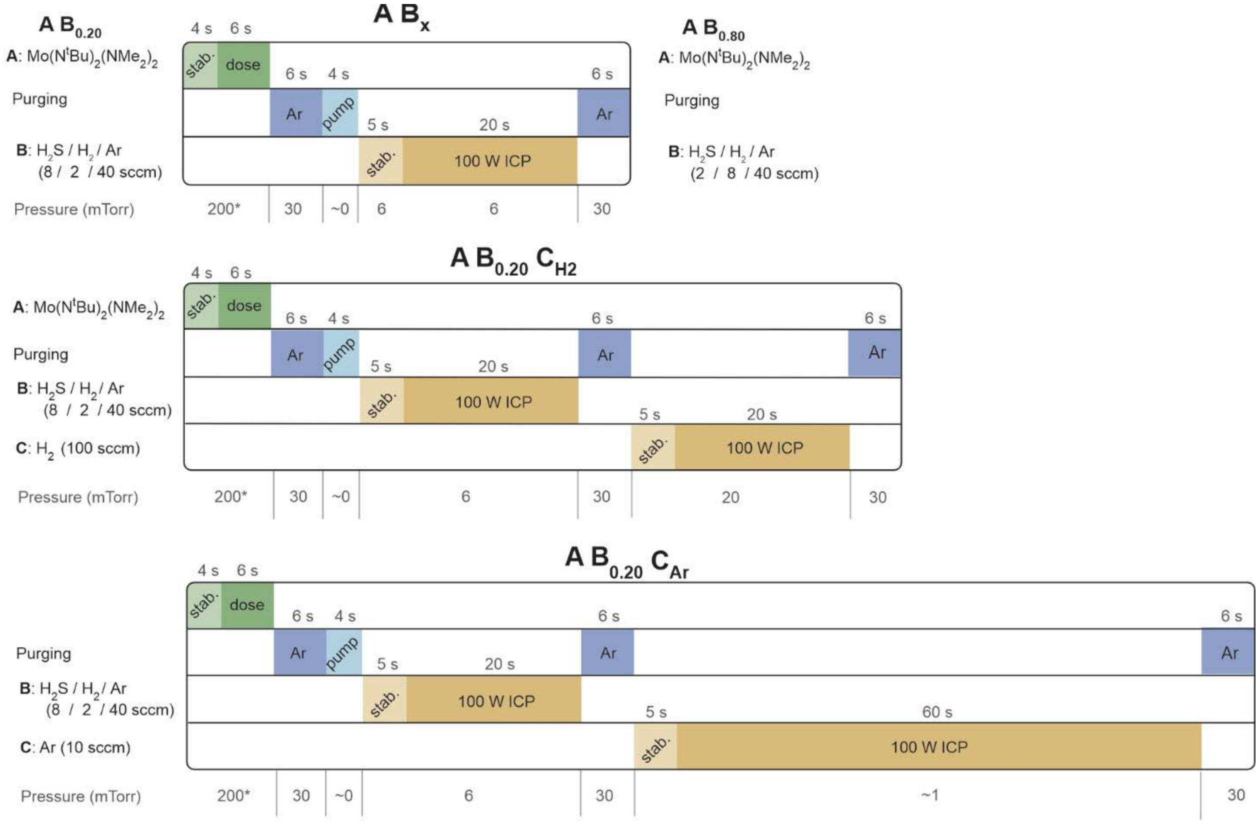

Figure S1. Schematic of the optimized ALD cycles of the ABC MoS_2 processes. The AB processes are also shown for reference. The lighter shading denotes gas flow/pressure stabilization (stab.) steps. For purging, both pumping with Ar flow into the chamber (denoted Ar) and without Ar flow (denoted pump) are used. Note that the n(A $B_{0.20}$) C_{H2} process consists of n-1 cycles of A $B_{0.20}$ process followed by one cycle of the A $B_{0.20}$ C_{H2} process.

S1.2: Accurate thickness control and uniformity

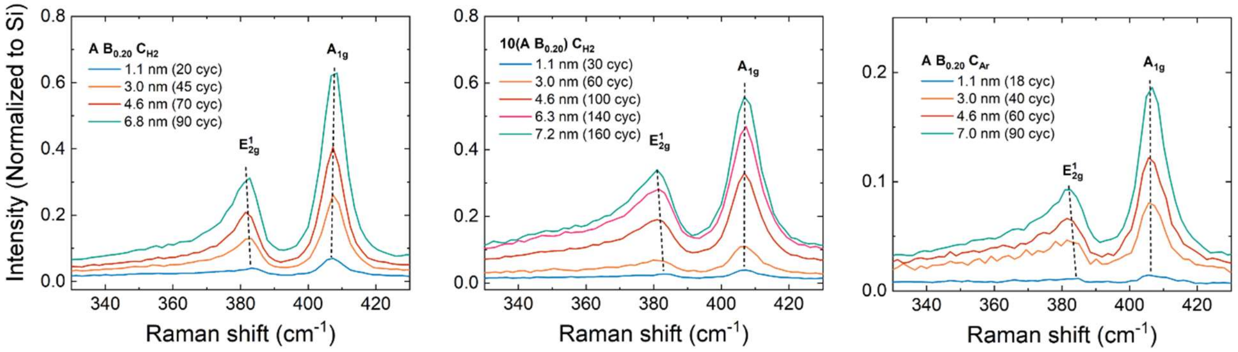

Figure S2. Raman spectra and SE thicknesses of MoS_2 films deposited using a varying number of ALD cycles demonstrating the accurate thickness control and crystallinity of the MoS_2 films.

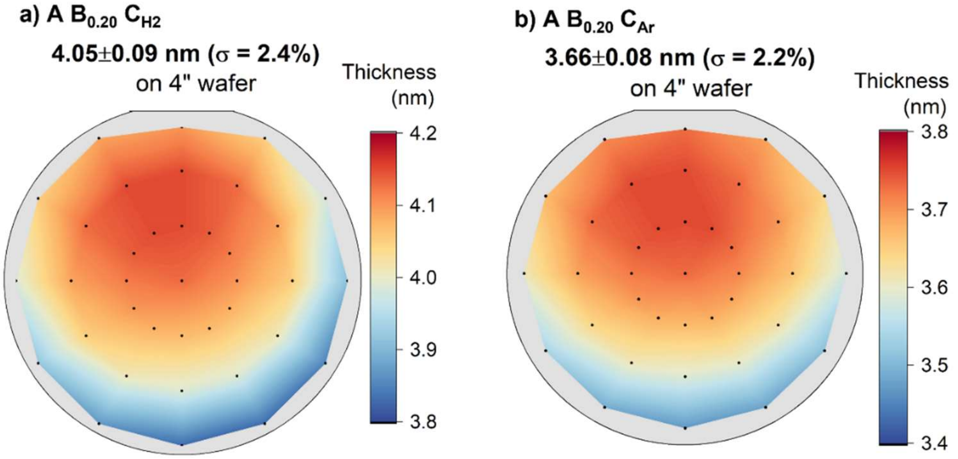

Figure S3. SE thickness maps showing uniform film deposition on 4"SiO_2/Si wafers using a) A $B_{0.20}$ C_{H2} and b) A $B_{0.20}$ C_{Ar} processes at 150 °C (60 ALD cycles). Average thickness and standard deviation (σ) are shown. Dots represent measurement points.

S2: Optimization of the three ABC processes discussed in the main text

S2.1: A $B_{0.20}$ C_{H2} process optimization

Our initial experiments indicated that adding a H_2 plasma C step to the A $B_{0.20}$ process, which deposits a-$MoS_{3.5}$ films at 150 °C, effectively removed extra sulfur from the films and allowed the films to crystallize as MoS_2 (Table S1). In contrast, H_2 gas had no effect on stoichiometry and crystallinity of the a-$MoS_{3.5}$ films, showing that plasma-generated species such as H radicals and/or ions are required. We note that as our aim is to deposit crystalline MoS_2 films, sulfur present at a higher stoichiometry is denoted *extra* or *excess S*. Nevertheless, sulfur-rich a-MoS_{2+x} films are of interest for selected applications, such as hydrogen evolution reaction (HER) electrocatalysis.[1–3]

We varied the exposure time to H_2 plasma as well as pressure during the H_2 plasma exposure. Even the shortest 5 s H_2 plasma exposure resulted in the desired MoS_2 stoichiometry and crystallization of the films, together with a near doubling of GPC and a decrease of resistivity of at least three orders of magnitude compared to the A $B_{0.20}$ process (Figure S4, Table S1). Increasing the H_2 plasma exposure time from 5 to 60 s only had a minor effect on film growth and properties, confirming the essentially self-limiting nature of the process. The slight decrease of GPC with increasing exposure time is likely due to changes in morphology, namely a decreased density of out-of-plane oriented fins that are known to result in increased GPC due to their high reactivity.[4–6] Although abundant fins were observed regardless of the exposure time, SEM and SE suggested fin density to decrease for longer H_2 plasma exposures (data not shown).

At the same time, the S/Mo ratio decreased only very slightly, showing that S is easily removed by H_2 plasma until MoS_2 stoichiometry – and perhaps a crystalline structure – is obtained, after which H_2 plasma becomes more inert as is desired for deposition of MoS_2. Nevertheless, a slight decrease in the S/Mo ratio with increasing exposure time suggests that H_2 plasma conditions can be used to tailor sulfur vacancy concentration in the MoS_2 films, which is known to be important for e.g. electrocatalytic applications.[7–9] The intensity of the E^1_{2g} and A_{1g} Raman peaks of MoS_2 stabilized after a 20 s H_2 plasma exposure, i.e. more slowly than the S/Mo ratio (Figure 2). Thus, a 20 s exposure was deemed saturating and used in further experiments.

Besides exposure time, the pressure during H_2 plasma was varied. Increasing pressure from our standard 20 mTorr to 100 mTorr resulted in a slight increase of S/Mo ratio by 0.1 (Table S1) and a decrease of crystallinity as shown by decreased intensity of the main MoS_2 Raman peaks (E^1_{2g} and A_{1g}), both in absolute terms and in terms of their ratio to defect-related E_{1g} and LA(M) peaks (Figure S5). When pressure was further increased to 300 mTorr, amorphous $MoS_{2.7}$ films were obtained. To first approximation, an increase of pressure results in a higher radical concentration produced in the ICP source. In contrast, both the energy and flux of ions reaching the substrate decrease substantially with increasing reactor pressure[10,11] The observation thus suggests that ions may have a beneficial effect in removal of excess S and/or crystallizing the films. Based on literature on ICP plasmas at comparable conditions, the dominant ion species is expected to be H_3^+, while H_2^+ and H^+ ions may also arrive at the substrate at comparable to 1–2 orders of magnitude lower fluxes.[12,13]

Earlier experiments in an Oxford Instruments FlexAL reactor similar to that used in this work suggest that for H_2 plasma at 20 mTorr, mean and maximum ion energies of 20–25 and 30–35 eV are expected.[11,14,15] The total ion flux is estimated to be 1–2×10^{14} cm^{-2} s^{-1}, which for a 20 s plasma exposure translates to 20–40 ions nm^{-2} $cycle^{-1}$. For comparison, RBS showed that 2.0 Mo atoms nm^{-2} $cycle^{-1}$ are deposited – consequently the number of S atoms deposited in a B step is (assuming $MoS_{3.7}$ stoichiometry by RBS) 7.4 S atoms nm^{-2}. The H_2 plasma C step then removes approximately 3.6 S atoms nm^{-2}. This order of magnitude calculation shows that a few (~1–10) H_n^+ ions are expected to arrive at the surface per deposited (removed) atom. It is worth noting that energy transfer from light H_n^+ ions (mass 1 to 3 u for $1 \leq n \leq 3$) upon collisions with film surface containing Mo (~96 u) and S (~32 u) is inefficient. However, H_n^+ ions are chemically highly reactive, which may improve their efficiency in removing the excess sulfur in a-MoS_x. Besides, radicals arriving at the substrate at much higher fluxes compared to ions are expected to play an important role in removing excess S.

We note that pressure control was achieved by throttling the pumping speed by an automatically controlled butterfly valve, which increases residence time of species in the chamber. This can result in redeposition of the removed sulfur, an effect that has been observed for SiN_x PEALD,[16] which could also explain the inefficient removal of sulfur at (comparably) high pressures. Although we do not know for certain if the effect of pressure is predominantly due to ions, redeposition, or something else, it is clear that use of low pressures during the H_2 plasma exposure is beneficial for deposition of crystalline MoS_2 films. We were unable to reliably strike H_2 plasma at pressures even lower than 20 mTorr. Thus, 20 mTorr was deemed optimal and used in further experiments.

Table S1. Summary of the effect of H_2 plasma conditions on film growth and properties in A $B_{0.20}$ C_{H2} process. The condition deemed optimized is shown in **bold**. GPC was determined by dividing SE thickness by the number of ALD cycles (140), resistivity by combining sheet resistance measured by four-point-probe (FPP) and SE thickness (detection limit (dl) of ~10^9 Ohm corresponds to ~4000 Ωcm for 10 nm thick films), and S/Mo ratio by XPS.

Exposure (s)	Pressure (mTorr)	ICP power (W)	GPC (Å)	ρ (Ωcm)	S/Mo ratio
		Effect of H_2 plasma vs. H_2 gas and no C step			
**-** (no C step)	-	-	0.67	>dl	3.54
20	20	0 (gas)	0.68	>dl	3.55
**20**	**20**	**100**	**1.11**	**9.3**	**1.89**
		Effect of exposure time			
5	20	100	1.21	3.0	1.97
10	20	100	1.18	3.9	1.95
**20**	**20**	**100**	**1.11**	**9.3**	**1.89**
60	20	100	0.96	13	1.85
		Effect of pressure			
**20**	**20**	**100**	**1.11**	**9.3**	**1.89**
20	100	100	1.21	1.8	1.99
20	300	600	0.88	47	2.70

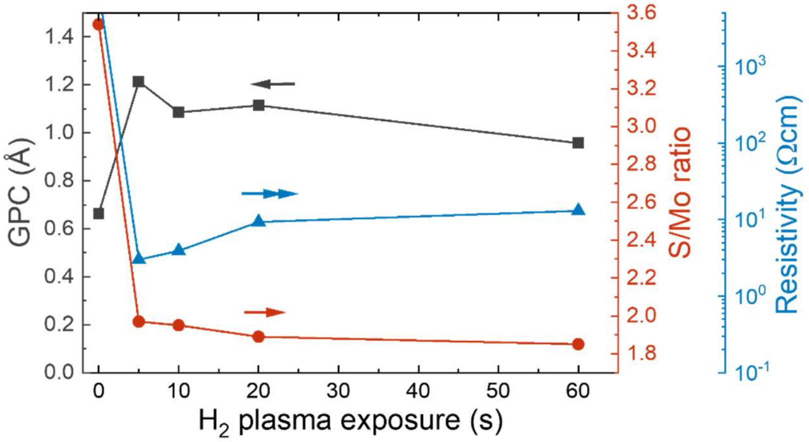

Figure S4. Effect of H_2 plasma exposure time (20 mTorr, 100 W) in A $B_{0.20}$ C_{H2} process on GPC (SE), S/Mo ratio (XPS), and resistivity (FPP). The films were deposited using 140 ALD cycles at 150 °C.

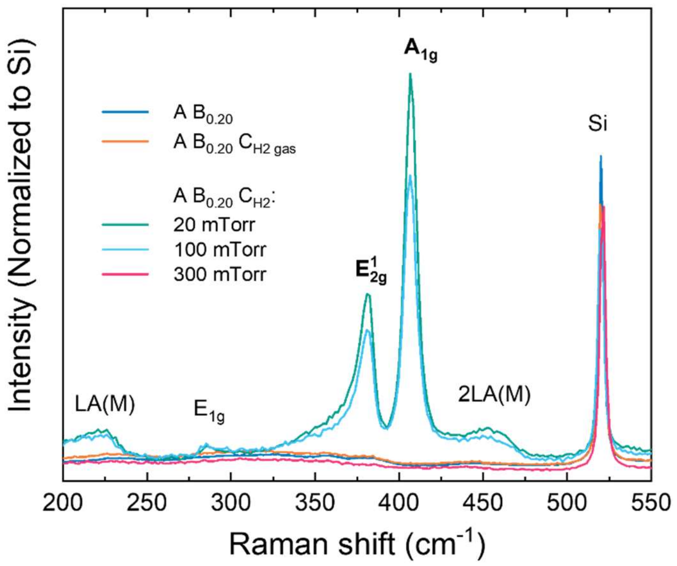

Figure S5. Raman spectra of MoS_x films deposited at different pressures during the H_2 plasma C step (A $B_{0.20}$ C_{H2} process). For comparison, A $B_{0.20}$ and $AB_{0.20}$ $C_{H2\ gas}$ processes are also shown. The films were deposited using 140 ALD cycles at 150 °C.

S2.2: A $B_{0.20}$ C_{H2} process at different temperatures

The experiments reported in the main text were performed at a low, plastic-compatible deposition temperature of 150 °C. Both lower and higher temperatures were briefly examined using the A $B_{0.20}$ C_{H2} process, showing that crystalline MoS_2 films can be deposited between 100 and 250 °C (Figure S6 and Table S2 – note that at 300 °C and above crystalline MoS_2 is deposited even without a C step). A somewhat S-deficient stoichiometry was observed at temperatures other than 150 °C, however, which may be improved by optimizing the H_2 plasma conditions separately at each temperature.

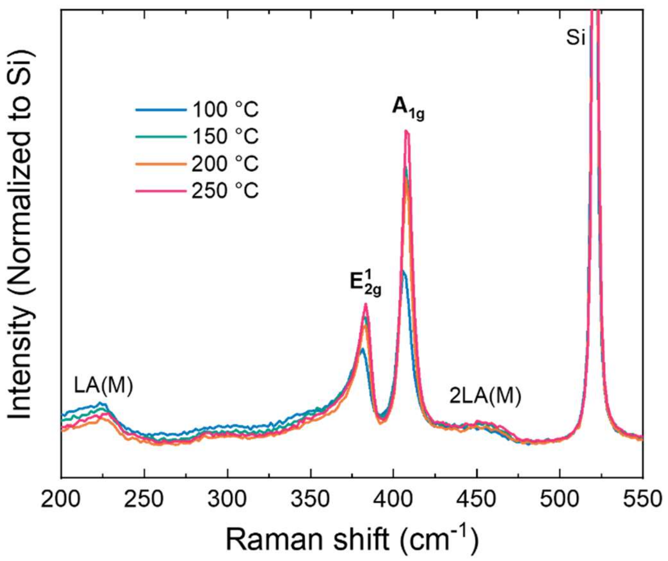

Figure S6. Raman spectra of MoS_x films deposited at different temperatures using the A $B_{0.20}$ C_{H2} process (see table below for deposition conditions, thicknesses, and other film characteristics).

Table S2. Summary of the effect of deposition temperature in A $B_{0.20}$ C_{H2} process. GPC was determined by dividing SE thickness by the number of ALD cycles, resistivity by combining sheet resistance measured by FPP and SE thickness, and S/Mo ratio by XPS.

T (°C)	ALD cycles	Thickness (nm)	GPC (Å)	ρ (Ωcm)	S/Mo ratio
100	50	4.3	0.86	0.86	1.66
150	45	3.0	0.67	29	1.89
200	50	2.9	0.58	60	1.62
250	50	2.7	0.54	81	1.78

S2.3: n(A $B_{0.20}$) C_{H2} process: effect of H_2 plasma frequency n on film growth and properties

This section contains data on the effect of H_2 plasma periodicity *n* on film growth and characteristics in the n(A $B_{0.20}$) C_{H2} process. In other words, the process consists of *n* cycles of the A $B_{0.20}$ process, which nominally deposits a-$MoS_{3.5}$ films at 150 °C followed by a H_2 plasma exposure to remove excess S and crystallize the films. The *n* cycle process can be repeated as many times as desired, but it is important to note that it always ends in a H_2 plasma exposure. The hydrogen plasma conditions correspond to those optimized for the A $B_{0.20}$ C_{H2} process (i.e., *n* = 1), that is a 20 s exposure to 20 mTorr H_2 plasma. We note that further optimizing or modifying the conditions for different values of *n* may provide further opportunities.

Table S3 summarizes the data for different sample sets with varying *n*, including those deposited using a fixed number of ALD cycles (~140) as well as films of similar thickness for facile comparison (6–8 nm and 3 nm). Some of the data has been presented in the main article (Figure 3), while additional figures are presented in this section on Raman (Figure S7) and XRD (Figure S8) of 3 nm films and AFM of 3 and 6–8 nm films (Figure S9). In addition, *in situ* SE data supporting the proposed growth mechanisms is presented in Figures S10 and S11 and optical constants extracted from SE measurements in Figure S12.

Table S3. Summary of the effect of *n* on film growth and properties in n(A $B_{0.20}$) C_{H2} process. GPC was determined by dividing SE thickness by the number of ALD cycles (140), resistivity by combining sheet resistance measured by FPP and SE thickness (detection limit (dl) of ~10^9 Ohm corresponds to ~4000 Ωcm for 10 nm thick films), roughness (R_q) by AFM, and S/Mo ratio by XPS.

n	Cycles	Thickness (nm)	GPC (Å)	ρ (Ωcm)	R_q (nm)	S/Mo ratio
- (no C step)	140	9.3	0.67	>dl	0.3	3.54
Effect of *n* (~140 cycles, XPS used in Figure 4a, SE in Figure S10b)						
1	140	15.6	1.11	9.3	3.6	1.89
3	138	8.2	0.59	4.8	1.3	1.89
5	140	7.7	0.55	6.1	0.8	1.91
10	140	6.3	0.45	8.7	0.4	1.93
20	140	5.8	0.41	8.4	0.3	1.85
Effect of *n* (6–8 nm films, used in Figures 4a,c,d, S9,S12)						
1	90	6.8	0.76	48	2.3	-
3	138	8.2	0.59	4.8	1.3	1.89
5	140	7.7	0.55	6.1	0.8	1.91
10	160	7.2	0.45	6.3	0.5	-
20	140	5.8	0.41	8.4	0.3	1.85
Effect of *n* (3 nm films, used in Figures S7–S9)						
1	50	3.0	0.60	29	1.2	-
3	54	3.3	0.61	51	0.7	-
5	55	3	0.55	64	0.5	-
10	60	3.0	0.50	48	0.5	-
20	60	3.1	0.52	87	0.4	-

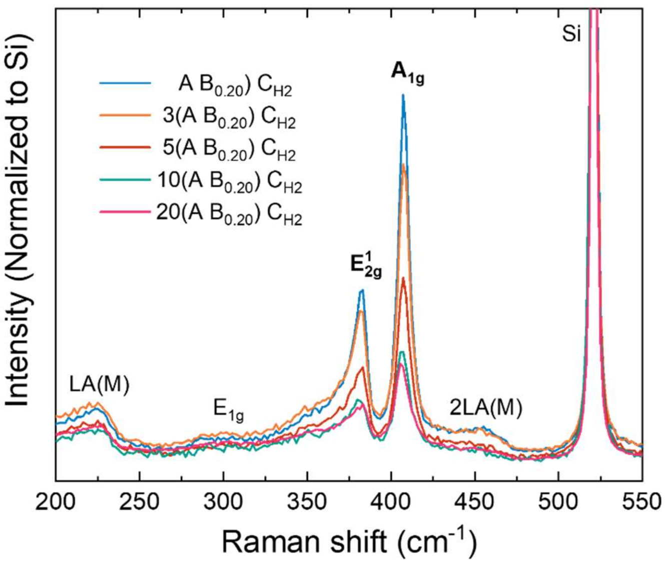

Figure S7. Raman spectra of ~3 nm MoS_2 films deposited using n(A $B_{0.20}$) C_{H2} processes with varying *n* at 150 °C.

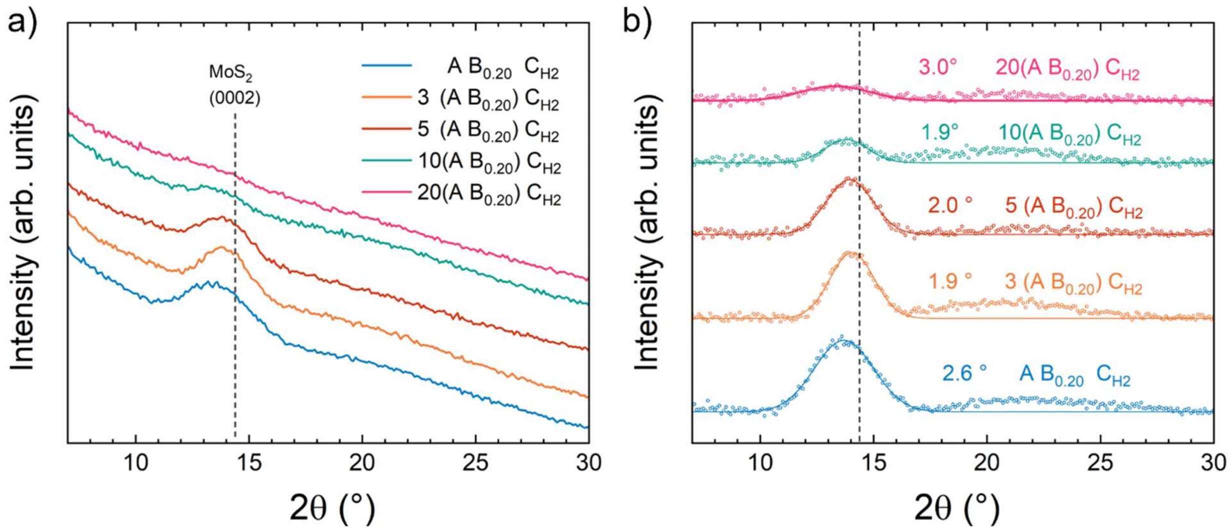

Figure S8. θ-2θ X-ray diffractograms of ~3 nm MoS_2 films deposited using n(A $B_{0.20}$) C_{H2} processes with varying *n* at 150 °C: a) raw data, b) data after background removal and (Gaussian) peak fits along with extracted FWHMs. Reference position of (0002)MoS_2 reflection (14.4 °2θ; JCDPS-ICDD powder diffraction file 00-037-1492) is shown. The broad peak centered at approximately 22 °2θ originates from the SiO_2 layer of the substrate. The data have been offset vertically for clarity.

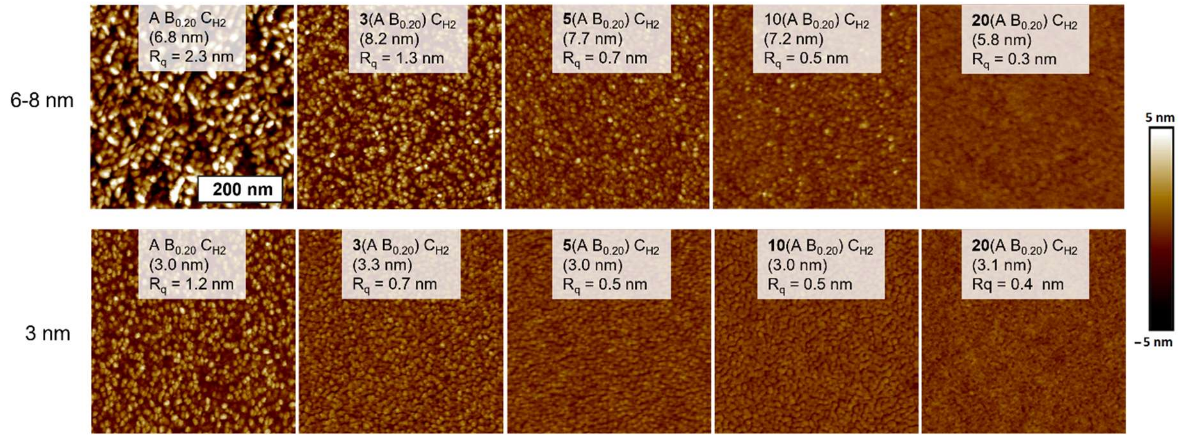

Figure S9. AFM images and roughness (R_q) values of films deposited using n(A $B_{0.20}$) C_{H2} processes with varying *n* at 150 °C to a thickness of approximately 6–8 nm (top row) and 3 nm (bottom row). Film thicknesses determined by SE are shown in parenthesis.

Figure S10a presents in-situ SE data for processes with 1 ≤ *n* ≤ 10 collected after each B and C step, showing that at first approximately 20 ALD cycles are needed to reach a stable GPC. Before this, both the thickness added during an AB cycle and the thickness removed during a C step are low. This can be understood by differences in reaction mechanisms when the film is growing on SiO_2 substrate in the initial cycles, i.e. film nucleation, as opposed to growth on itself in the following cycles. Film growth in the latter, "steady-state", regime will be further discussed below.

Figure S10b shows measurements performed after every 10 cycles (9 for n = 3) illustrating the trends in GPC with increasing film thickness up to several nanometers. Clearly non-linear growth is observed for *n* = 1, which is linked to formation of out-of-plane oriented fins that grow faster than basal planes.[5,6] In contrast, linear growth is observed for *n* ≥ 3, in line with smooth(er) morphology and the proposed growth mechanism based on isotropic a-MoS_{2+x} deposition followed by removal of excess S and crystallization by H_2 plasma.

Further support to the proposed growth mechanism, in particular deposition of a-MoS_{2+x} on c-MoS_2 surface during the process, was obtained from an experiment where four 10(A $B_{0.20}$) C_{H2} "supercycles", i.e., a total of 40 ALD cycles, were first applied resulting in a c-$MoS_{1.9}$ film as described above. Then, 10 A $B_{0.20}$ cycles presumably depositing a-$MoS_{3.5}$ were applied. XPS showed the resulting film to have an average stoichiometry of $MoS_{2.2}$ and a S component attributed to S_2^{2-} species only present in a-MoS_{2+x} and not in c-MoS_2 was clearly observed, proving the surface layer was sulfur-rich as expected. The XPS information depth exceeds the film thickness in this case, as shown by the fact that Si 2p peaks were observed, which means that the stoichiometry is averaged over the amorphous, sulfur-rich layer deposited using 10 cycles as well as the 40 cycle c-MoS_2 layer underneath.

Figure S11 shows the apparent thickness change as well as change in Ψ – which is related to the amplitude of the reflected light measured by SE – after each B and C step. Obviously, the apparent thickness increased – which corresponds to a decrease in Ψ in this case – after each B step and the reverse happened after a H_2 plasma C step. For *n* = 1, the thickness decrease (Ψ increase) after a C step was clearly lower than the corresponding thickness increase (Ψ decrease) after a B step, as is required for film growth. For larger values of *n*, the apparent thickness decrease after the C step increased in magnitude, although it did not scale linearly with *n* or even the apparent thickness deposited in the preceding *n* A $B_{0.20}$ cycles. This is

attributed to the very different optical properties of a-$MoS_{3.5}$ and MoS_2 (Figure S12) and the use of fixed c-MoS_2 constants in the model. Attempts to model the amorphous surface layer separately were unsuccessful.

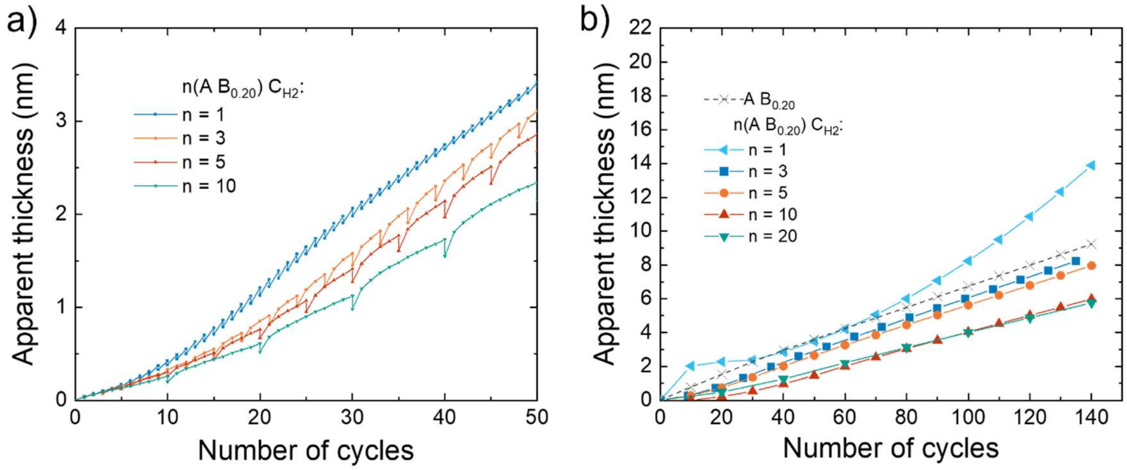

Figure S10. *In situ* SE thicknesses of MoS_2 films deposited at 150 °C using n(A $B_{0.20}$) C_{H2} processes with varying *n*: a) First 50 cycles measured after every B and C step and b) 140 cycles measured every 10 cycles (9 for *n* = 3 – note that for *n* = 20 only every other data point is after H_2 plasma).

Furthermore, the apparent GPC was not constant during *n* A $B_{0.20}$ cycles when *n* > 1. Instead, it was the largest during the first cycle before quickly decreasing to a value approximately corresponding to steady state a-$MoS_{3.5}$ deposition using the A $B_{0.20}$ process. Although the assumed optical constants prevent reliable quantitative extraction of cycle-by-cycle GPC, the decrease of GPC with increasing *n* was confirmed by measurements of film thickness – and thus the average GPC – after deposition.

The steady state GPC of the A $B_{0.20}$ process is 0.7 Å, while the observed GPC of the 10th A $B_{0.20}$ cycle in 10(A $B_{0.20}$) C_{H2} process was 0.4–0.5 Å. The difference is attributed to the use of c-MoS_2 optical constants in the model, as a-$MoS_{3.5}$ has substantially lower optical constants than c-MoS_2. A simple calculation based on the GPC of the A $B_{0.20}$ process (0.7 Å) and composition change from $MoS_{3.5}$ to $MoS_{1.9}$ suggests a GPC of 0.43 Å, which is very close to that observed at *n* = 20 (0.41 Å), and is in line with the growth approaching steady state growth of the A $B_{0.20}$ process at larger values of *n*.

The GPC during the first A $B_{0.20}$ cycle following a C step increased for larger *n*, which may have a few possible origins. For example, converting a thicker a-MoS_{2+x} layer to c-MoS_2 during the C step, as occurs for larger *n*, might result in a higher density of reactive sites on the surface. This may be linked to contraction of the film upon a-MoS_{2+x} to c-MoS_2 conversion, which can lead to an increased surface area. Another possibility is increased sulfurization of film surface, which linked to the above argument of increasing surface area might also scale with *n*. The above-explained assumption of fixed optical constants should also be kept in mind. However, the fact that the changes in Ψ follow the trends in apparent GPC shows that the change in the optical properties is indeed the largest during the first A $B_{0.20}$ cycle.

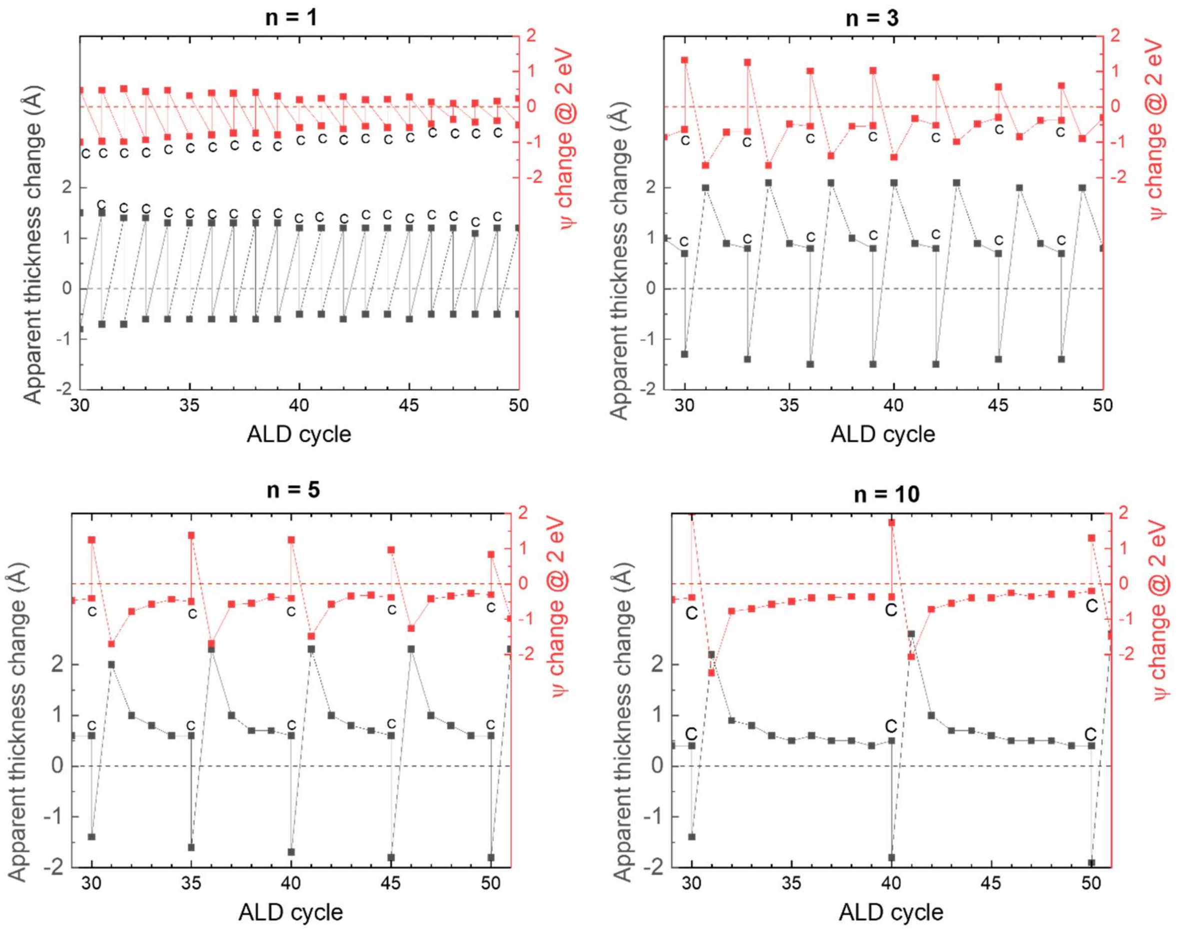

Figure S11. Change in apparent thickness and ellipsometry parameter Ψ (at 2.0 eV) after each B and C step for n(A $B_{0.20}$) C_{H2} processes at 150 °C with varying *n*. Measurements were performed after each B and C step. Data points measured just before a C step are marked with an asterisk (i.e. C step corresponds to the "vertical transitions" in the graph). Range from 30 to 50 ALD cycles is shown to exclude nucleation effects (cf**.** Figure S10a**).**

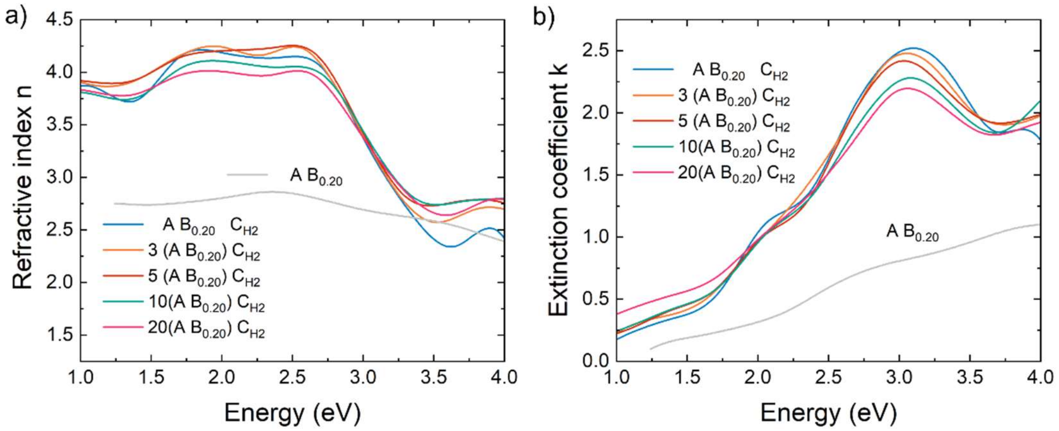

Figure S12. Optical constants: a) n and b) k of approximately 5–8 nm thick MoS_2 films deposited using varying H_2 plasma periodicity *n* as extracted from *in situ* SE data using a B-spline model.

S2.4: A $B_{0.20}$ C_{Ar} process optimization

Our initial trials of the A $B_{0.20}$ C_{Ar} process were done using 6 mTorr pressure similar to our B step and a 20 s plasma exposure similar to most other plasmas used in this work. This resulted in only partial removal of excess S, from $MoS_{3.5}$ to $MoS_{3.0}$ (Table S4). Increasing the ICP power from 100 to 500 W was found to provide only a slight improvement (a-$MoS_{2.8}$). However, as ICP powers higher than 100 W were observed to result in unreliable plasma ignition especially at lower pressures, the ICP power was fixed at 100 W.

Increasing exposure time at 6 mTorr from 20 to 60 s helped with the removal of excess S, resulting in $MoS_{2.4}$ stoichiometry and emergence of Raman peaks attributed to crystallization of MoS_2. In addition, decreasing pressure from 6 to 1 mTorr, which increases ion dose and energy (mean ion energy and flux estimated at 25–30 eV and 2–3 × 10^{14} Ar^+ ions cm^{-2} s^{-1} at 1 mTorr - see Section S2.6), resulted in more efficient removal of excess S. $MoS_{2.7}$ stoichiometry and emergence of weak MoS_2 Raman peaks were observed already after a 20 s exposure at 1 mTorr.

Combining the low pressure and long exposure time produced the best results: a 60 s Ar plasma exposure at 1 mTorr yielded $MoS_{2.3}$ films with a Raman response similar to films deposited using the A $B_{0.80}$ process. A further increase of exposure time to 120 s decreased S/Mo ratio to 2.1, but this had only a minor effect on the Raman spectrum (Figure S13). The increase of crystallinity was supported by changes in optical properties extracted from SE. Optical constants resembling c-MoS_2 the most closely were observed for the longest exposure times at 1 mTorr (Figure S14; e.g., high refractive index at 1–3 eV, rapidly increasing extinction coefficient at energies above the bulk band gap of ~1.3 eV).

A control experiment using Ar gas instead of Ar plasma had no effect on film stoichiometry, showing that the process is indeed driven by Ar^+ ions and not, for example, thermal decomposition of the MoS_x film.

The Ar plasma conditions had a large effect on resistivity (Table S4). The lowest resistivities of 6–12 Ωcm were observed for weakly crystalline, sulfur-rich (S/Mo = 2.7–2.8) films deposited using a short 20 s plasma exposure. Increasing the plasma exposure time, which decreased S/Mo ratio and improved crystallinity as described above, increased resistivity; at 1 mTorr from 6 to 220 to 1500 Ωcm at 20, 60, and 120 s exposure times. These resistivities are much higher compared to our other ABC processes and are hypothesized to result from low H concentration in the films and consequently low carrier densities, as described in the main text (Section 2.3.3).

In situ SE showed linear growth at all conditions used for the A $B_{0.20}$ C_{Ar} process. Compared to the A $B_{0.20}$ process, all of the conditions exhibited a somewhat higher GPC of 0.8–0.9 Å. With increasing Ar^+ ion bombardment, i.e., increasing exposure time and decreasing pressure, GPC decreased slightly. Comparing the rather modest changes in GPC to the decrease of S/Mo ratio and likely increase of physical density due to crystallization, it appears that Ar^+ ion bombardment creates more adsorption sites for $Mo(N^tBu)_2(NMe_2)$.

Additional *in situ* SE experiments performed after each B and C step similar to those done for the n(A $B_{0.20}$) C_{H2} process showed a larger apparent thickness increase during A and B steps followed by a small apparent thickness decrease during the C step. The changes in Ψ were of the opposite sign compared to thickness. The apparent thickness and Ψ changes during the C step were smaller compared to the A $B_{0.20}$ C_{H2} process (Section S2.3).

SEM showed the films deposited using various Ar plasma conditions to be rather smooth (Figure S16). Formation of clear surface features, probably grains, and some roughness with increasing Ar plasma exposure and thus increasing crystallinity was, however, evident.

Summing all of the above results together, the A $B_{0.20}$ C_{Ar} process allows deposition of crystalline, slightly sulfur-rich, rather smooth films with higher resistivity. As no ideal saturation was reached and to keep the length of the ALD cycle reasonable, a 60 s exposure at 1 mTorr pressure was chosen for further experiments.

Table S4. Summary of the effect of Ar plasma conditions on film growth and properties in A $B_{0.20}$ C_{Ar} process. The condition deemed optimized is shown in **bold**. GPC was determined by dividing SE thickness by the number of ALD cycles (140), resistivity by combining sheet resistance measured by FPP and SE thickness (detection limit (dl) of ~10^9 Ohm corresponds to ~4000 Ωcm for 10 nm thick films), crystallinity by Raman spectroscopy, and S/Mo ratio by XPS

Exposure time (s)	Pressure (mTorr)	ICP power (W)	GPC (Å)	ρ (Ωcm)	S/Mo ratio
**-** (no C step)	-	-	0.67	>dl	3.54
**20**	6	0 (gas)	0.73	>dl	3.20
		Effect of pressure, exposure			
20	~1	100	0.91	6.3	2.68
**60**	**~1**	**100**	**0.81**	**220**	**2.32**
120	~1	100	0.76	1500	2.13
20	6	100	0.96	78	2.97
60	6	100	0.84	18	2.37

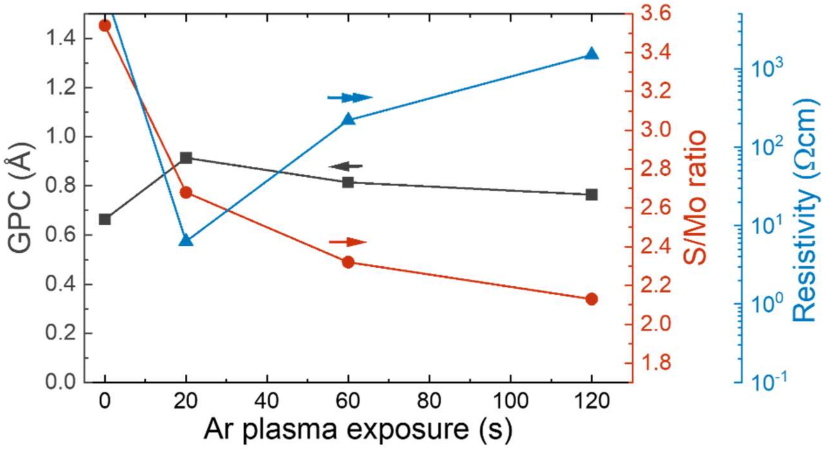

Figure S13. Effect of Ar plasma exposure time (1 mTorr, 100 W) in A $B_{0.20}$ C_{Ar} process on GPC (SE), S/Mo ratio (XPS), and resistivity (FPP). The films were deposited using 140 ALD cycles at 150 °C.

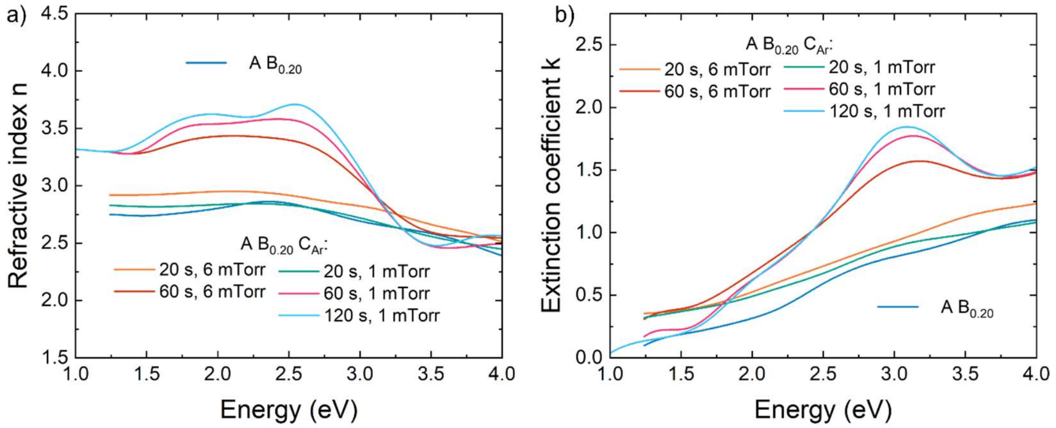

Figure S14. Optical constants: a) n and b) k of MoS_x films deposited using the A $B_{0.20}$ C_{Ar} process with varying Ar plasma conditions as extracted from *in situ* SE data using a B-spline model. The A $B_{0.20}$ process is shown for comparison.

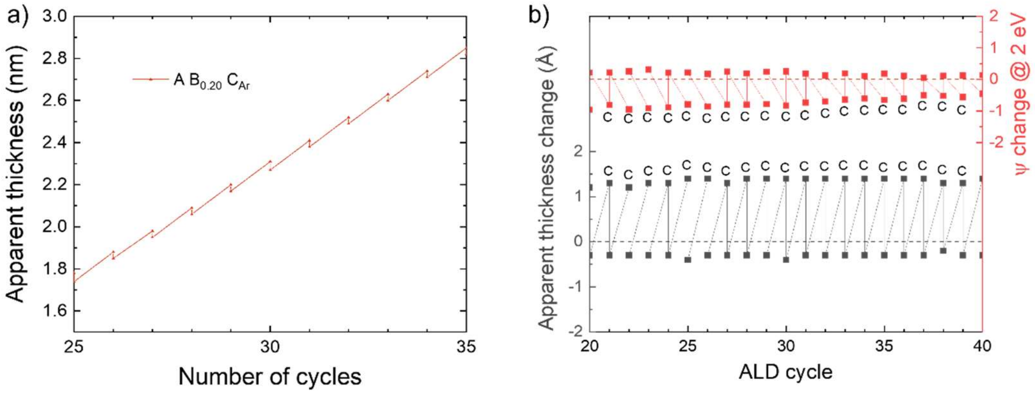

Figure S15. a) *In situ* SE derived apparent thickness and b) change in apparent thickness and ellipsometry parameter Ψ (at 2.0 eV) for A $B_{0.20}$ C_{Ar} processes at 150 °C. Measurements were performed after each B and C step. Data points measured just before a C step are marked with an asterisk (i.e. C step corresponds to the "vertical transitions" in the graph).

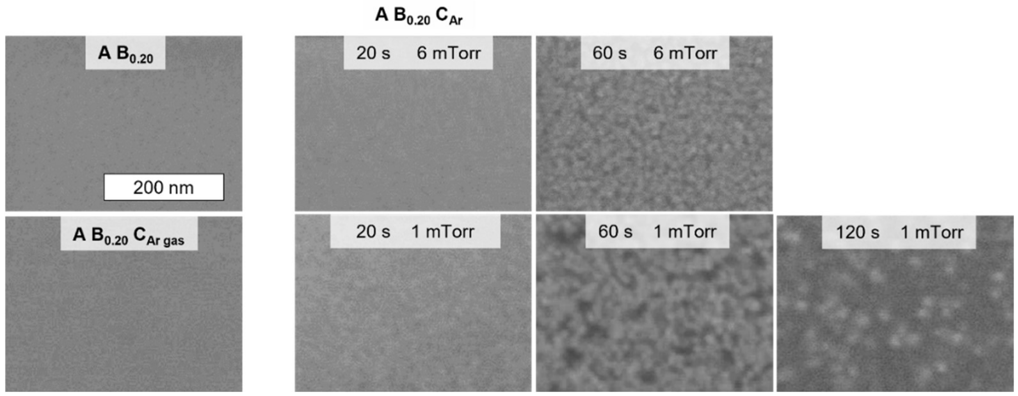

Figure S16. SEM images of MoS_x films deposited using different Ar plasma conditions (exposure time, pressure) in the A $B_{0.20}$ C_{Ar} process. For comparison, A $B_{0.20}$ and $AB_{0.20}$ $C_{Ar\ gas}$ processes are also shown. The films were deposited using 140 ALD cycles at 150 °C.

S2.5: Substrate biasing in A $B_{0.20}$ $C_{Ar\ bias}$ process

We also explored the use of RF substrate biasing to further increase the energy of Ar^+ ions. By applying an RF bias to the substrate table, the voltage over the plasma sheath increases, which results in additional energy given to the ions bombarding the substrate.[11,17] The reactor used in this work allows for reading the DC self-bias resulting on the substrate table, which can be taken as an estimate for additional energy given to the ions and will be used here to describe the biasing conditions. Ion energy estimates are presented in Section S2.6.

Matching of the biasing circuitry was found to be unreliable at 1 mTorr. Thus, the biasing experiments were performed at 6 mTorr pressure. The lowest reliably matched RF biasing power was 4 W, resulting in a self-bias of approximately 30 V for a 6 mTorr Ar plasma with 100 W ICP power. Higher bias voltages of approximately 45 and 60 V were also examined. All of the biasing conditions resulted in S/Mo ratios of 2.3–2.4 after a 20 s exposure, showing more efficient removal of excess S compared to the unbiased Ar plasma, but no clear trend with bias voltage (Table S5, Figure S17). However, the intensity of Raman modes decreased with increasing bias voltage. Furthermore, the intensities were clearly lower compared to the optimized Ar plasma condition that resulted in a similar stoichiometry (Figure S19a).

As the lowest 30 V bias resulted in the highest crystallinity of the investigated biasing conditions, the effect of exposure time at this condition was explored. Increasing the exposure time from 10 to 60 s decreased the S/Mo ratio from 2.5 to 2.2 (Figure S18). However, the crystallinity remained modest (Figure S19b). The refractive index of the films deposited using biasing remained rather low, although the dispersion resembled c-MoS_2 (Figure S20). This may suggest higher ion energies to result in porous films. While not unambiguously confirming the porosity, SEM showed the films became rougher with increasing bias voltage and exposure time (Figure S21).

Table S5. Summary of the effect of RF bias conditions on film growth and properties in A $B_{0.20}$ $C_{Ar\ bias}$ process. GPC was determined by dividing SE thickness by the number of ALD cycles (140), resistivity by combining sheet resistance measured by FPP and SE thickness (detection limit (dl) of ~10^9 Ohm corresponds to ~4000 Ωcm for 10 nm thick films), and S/Mo ratio by XPS

Exposure time (s)	RF bias (V) and power	Pressure (mTorr)	ICP power (W)	GPC (Å)	ρ (Ωcm)	S/Mo ratio
**-** (no C step)	-	-	-	0.67	>dl	3.54
60	0	~1	100	0.81	220	2.32
		Effect of bias voltage (20 s exposure)				
20	0	6	100	0.96	78	2.97
20	30 (4 W)	6	100	0.83	94	2.33
20	45 (6 W)	6	100	1.27	490	2.36
20	60 (8 W)	6	100	1.30	360	2.43
		Effect of exposure time (30 V bias)				
10	30 (4 W)	6	100	0.94	9.1	2.45
20	30 (4 W)	6	100	0.83	94	2.33
60	30 (4 W)	6	100	1.34	360	2.24

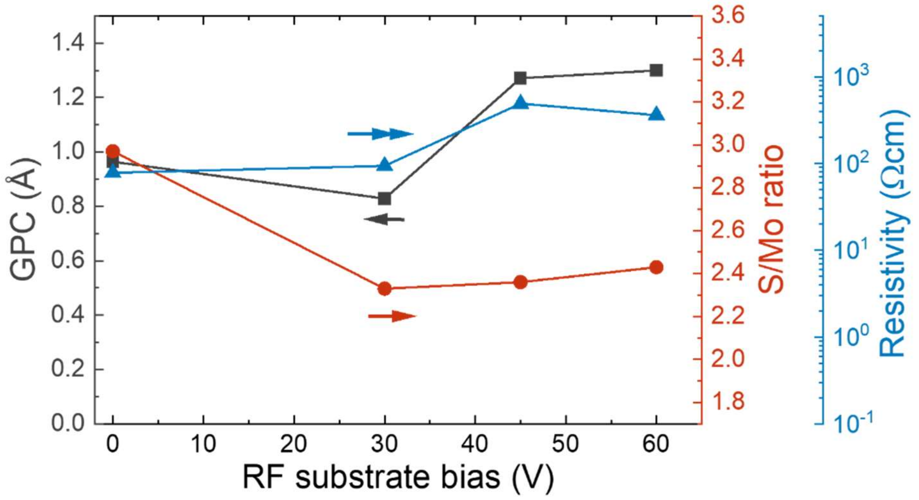

Figure S17. Effect of RF bias voltage in A $B_{0.20}$ $C_{Ar\ bias}$ process on GPC (SE), S/Mo ratio (XPS), and resistivity (FPP). The films were deposited using 140 ALD cycles at 150 °C.

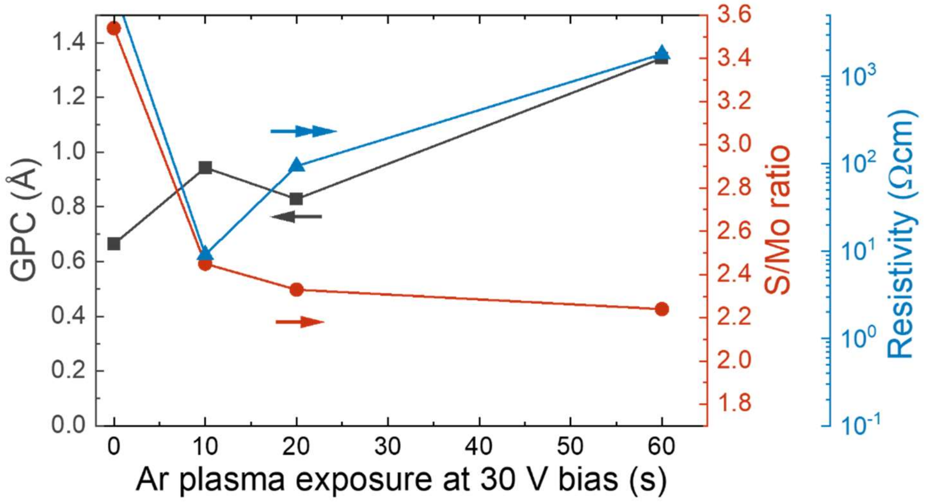

Figure S18. Effect of Ar plasma exposure time at 30 V RF substrate bias in A $B_{0.20}$ $C_{Ar\ bias}$ process on GPC (SE), S/Mo ratio (XPS), and resistivity (FPP). The films were deposited using 140 ALD cycles at 150 °C.

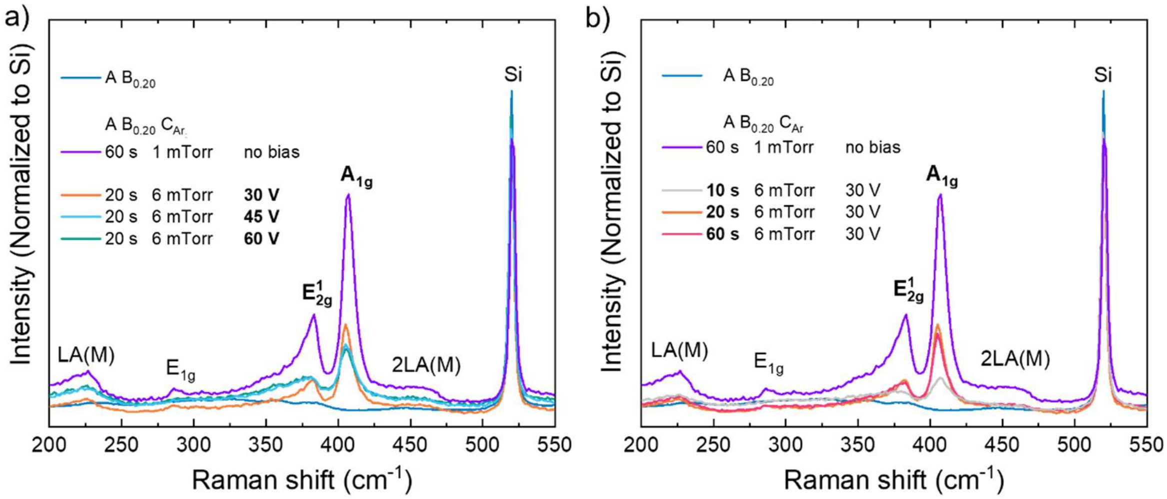

Figure S19. Raman spectra of MoS_x films deposited using different a) RF bias voltages and b) Ar plasma exposure times in the A $B_{0.20}$ $C_{Ar\ bias}$ process. For comparison, also A $B_{0.20}$ and A $B_{0.20}$ C_{Ar} (1 mTorr, 60 s, no bias) processes are shown. The films were deposited using 140 ALD cycles at 150 °C.

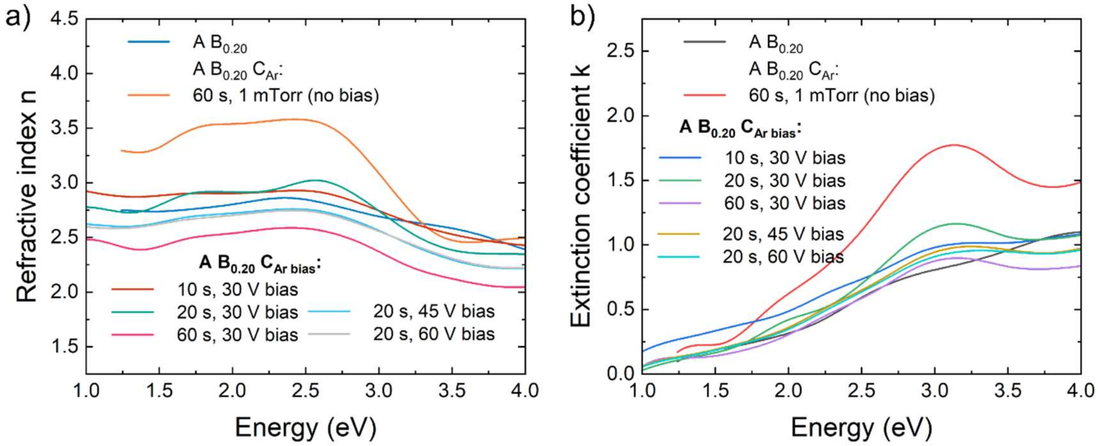

Figure S20. Optical constants: a) n and b) k of MoS_x films deposited using the A $B_{0.20}$ $C_{Ar\ bias}$ process with varying RF bias voltages and Ar plasma exposure times as extracted from *in situ* SE data using a B-spline model. The A $B_{0.20}$ process and optimized A $B_{0.20}$ C_{Ar} process are shown for comparison.

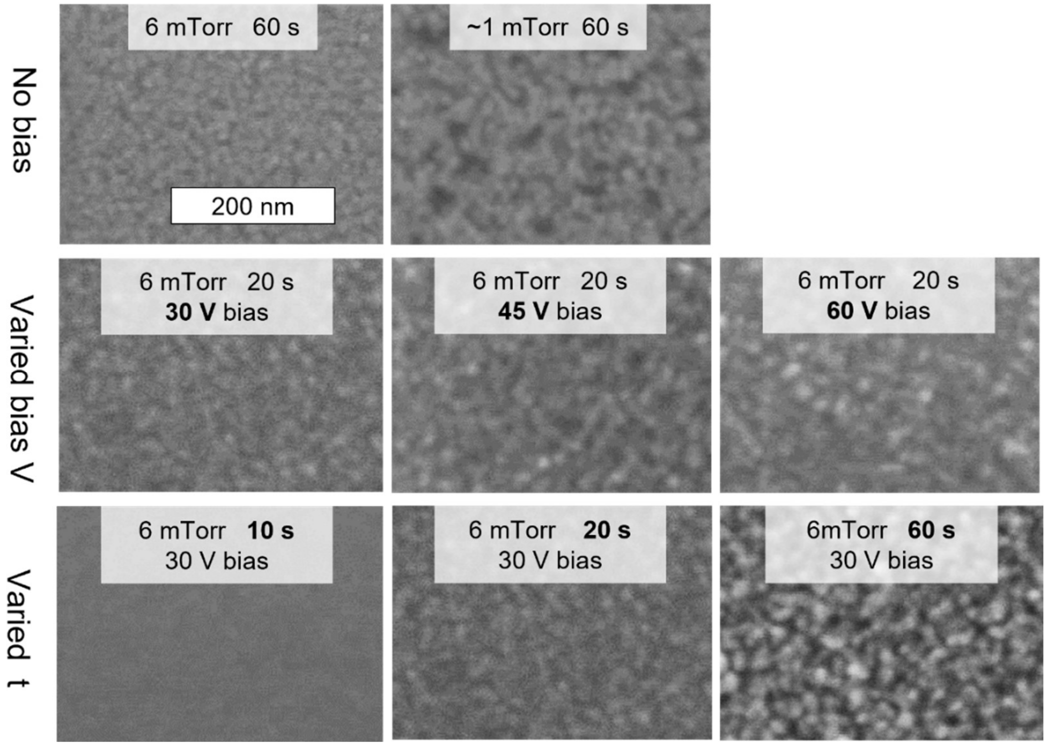

Figure S21. SEM images of MoS_x films deposited using different RF substrate bias voltages and Ar plasma exposure times in the A $B_{0.20}$ $C_{Ar\ bias}$ process. For comparison, also selected A $B_{0.20}$ C_{Ar} processes are shown. The films were deposited using 140 ALD cycles at 150 °C.

S2.6: Ion energy and flux estimates based on literature

To obtain more insight into the role of Ar plasma in the A $B_{0.20}$ C_{Ar} process and the effect of different plasma conditions and biasing, published ion energy and flux data obtained in a similar Oxford Instruments FlexAL reactor were analyzed.[10,11,14,18] This data combined with the trends of different parameters on ion energy distribution discussed in Refs.[10,11,18] were used to estimate ion energies and fluxes of plasma conditions used in this work (Table S6).

For Ar plasma operated at 6 mTorr, a rather narrow ion energy distribution is expected with mean and maximum ion energies estimated at 15–20 and 20–25 eV. The dominant ion species is Ar^+.[13,19] While the ion energies are consistent between different studies and thus can be predicted with relatively certainty, more scatter exists in the reported ion fluxes. Furthermore, ICP power affects ion flux, while it does not (for ideal inductive coupling) affect ion energies. Literature data at 100 W ICP power is not available. The ion flux at 6 mTorr using 100 W Ar plasma is estimated to be in the range of $1\text{–}2\times10^{14}$ $cm^{-2}\ s^{-1}$.

Decreasing pressure from 6 to 1 mTorr increases both ion energy – mean energy is estimated to increase by approximately 10 eV reaching 25–30 eV – and the ion flux, which we estimate to increase by a factor of two. Thus, we estimate that decreasing Ar plasma pressure from 6 to 1 mTorr increases the ion energy flux arriving at the substrate approximately by a factor of 3.

Table S6. Literature values for ion energies and fluxes for an Ar plasma in an Oxford Instruments FlexAL ALD reactor similar to that used in this work and estimates for selected Ar plasma conditions used in this work. Dash indicates that information is not available.

Pressure (mTorr)	ICP power (W)	Mean ion E (eV)	Max ion E (eV)	Ion flux (ion $cm^{-2}\ s^{-1}$)	Ion energy flux (eV $nm^{-2}\ s^{-1}$)	Ref.
			Literature			
9	200	15	20	3×10^{14}	44	11
30	200	9	-	7×10^{13}	6	
30	600	10	-	1×10^{14}	10	
3	200	21	-	1.5×10^{14}	31	18
15	500	12	25	-	-	14
9	200 + 50 V bias	15 (no bias)<br>65 (biased)	20 (no bias)<br>85 (biased)	3.0×10^{14}<br>3.5×10^{14}	224	11
			Estimates for plasmas used in this work			
1	100	25–30	30–35	$2\text{–}4\times10^{14}$	60–140	
6	100	15–20	20–25	$1\text{–}2\times10^{14}$	20–50	
	30 V bias	45–50	55–60	Similar	3× unbiased	
	45 V bias	60–65	75–80	Similar	4× unbiased	
	60 V bias	75–80	~100	Similar	5× unbiased	

RF substrate biasing can be used to increase ion energy with only a minor effect on the ion flux.[11] Ar plasma operated at 6 mTorr was used for biasing experiments. The mean additional energy given to ions corresponds approximately to a DC self-bias that is measured on the substrate table. RF biasing also causes the ion energy distribution to broaden, meaning that ions both substantially below and, importantly,

above the mean energy are observed.[11] For the lowest stable, 30 V bias, we estimate mean and maximum ion energies to be 45–50 eV and 55–60 eV. For the highest investigated, 60 V bias condition, the mean and the maximum ion energies are expected to be approximately 75–80 eV and 100 eV. Assuming insignificant changes in ion flux, the ion energy flux is estimated to increase by a factor of 3 (30 V bias) to 5 (60 V bias) compared to the unbiased case.

The estimated ion (energy) fluxes can be used to place the optimized process conditions into perspective regarding ion bombardment. For example, for an optimized Ar plasma exposure (1 mTorr, 60 s), approximately 3 Ar^+ ions with mean ion energy of 27.5 eV arrive on surface per nm^2 per s, resulting in an ion energy dose of approximately 5000 eV nm^{-2}. These numbers are based on average values within the estimated ranges and are to be seen as order of magnitude estimates – the ion energy dose can vary from 3000 to 7200 eV within the range of estimates for this condition.

The number of removed S atoms can be estimated from the RBS derived GPC for the A $B_{0.20}$ C_{Ar} process (using 60 s Ar plasma exposure at 1 mTorr), which is 1.3 Mo at. nm^{-2} $cycle^{-1}$. The stoichiometry after B step is $MoS_{3.7}$ (A $B_{0.20}$ process), while the final stoichiometry after C step is $MoS_{2.3}$. This means that 4.9 S at. nm^{-2} are deposited in the B step while the final film incorporates 3.0 S at. nm^{-2}, meaning that 1.9 S at. nm^{-2} are removed during the Ar plasma C step. Therefore, using the ion (energy) flux values calculated above, approximately 100 Ar^+ ions corresponding to a total energy of approximately 3000 eV are needed to remove one S atom using this Ar plasma condition.

Using 30 V bias, $MoS_{2.3}$ stoichiometry is achieved after a 20 s exposure to Ar plasma. Assuming the density of this film is the same as that deposited using 60 s Ar plasma exposure at 1 mTorr (RBS data for the bias condition is unavailable), this corresponds to approximately 20 Ar^+ ions with a total energy of ~1000 eV per removed S atom. Therefore, higher energy Ar^+ ions appear more effective in removing S from a-MoS_{2+x} films, both in terms of the number of ions and total energy provided by them. However, as discussed in the preceding sections, ion energies substantially above 30 eV have a detrimental effect on crystallinity of MoS_2 films. The minimum energy required to remove excess S atoms and crystallize the films is not known.

S3: Comparison of material properties

S3.1: Crystallinity, microstructure, and morphology

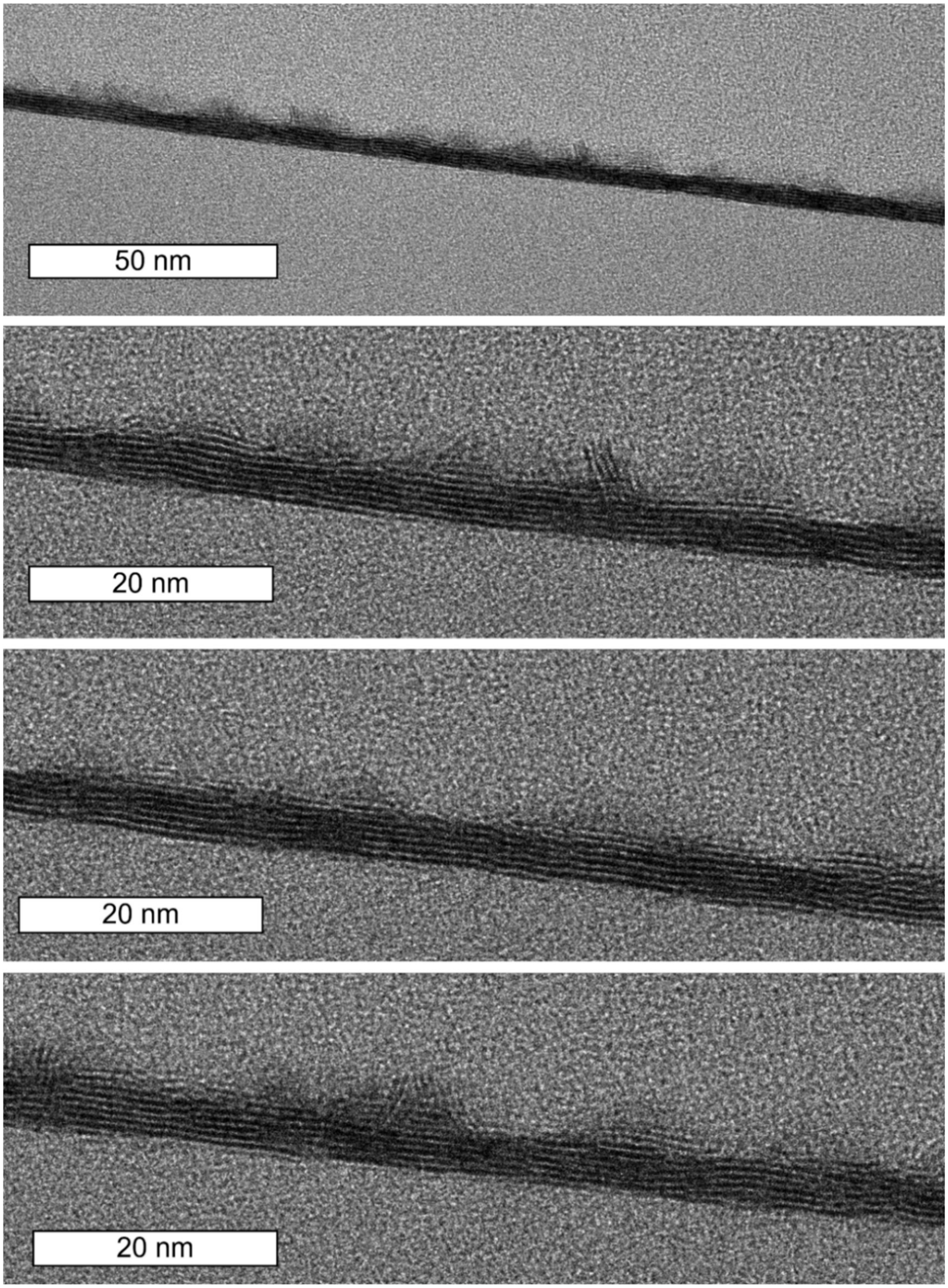

Figure S22. Additional cross-sectional TEM images of the A $B_{0.20}$ C_{H2} sample shown in Figure 7a showing different magnifications and locations.

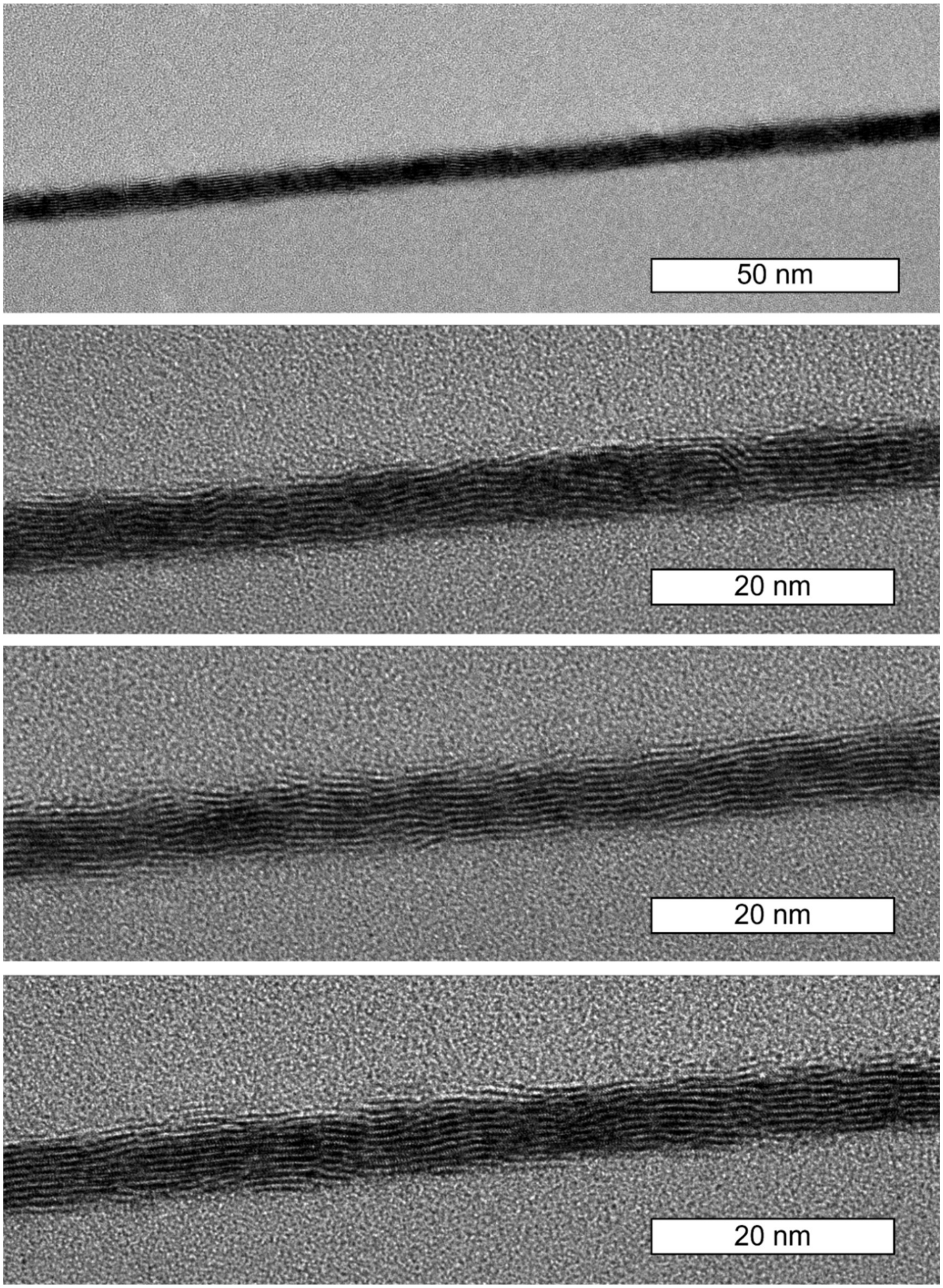

Figure S23. Additional cross-sectional TEM images of the 10(A $B_{0.20}$) C_{H2} sample shown in Figure 7b showing different magnifications and locations.

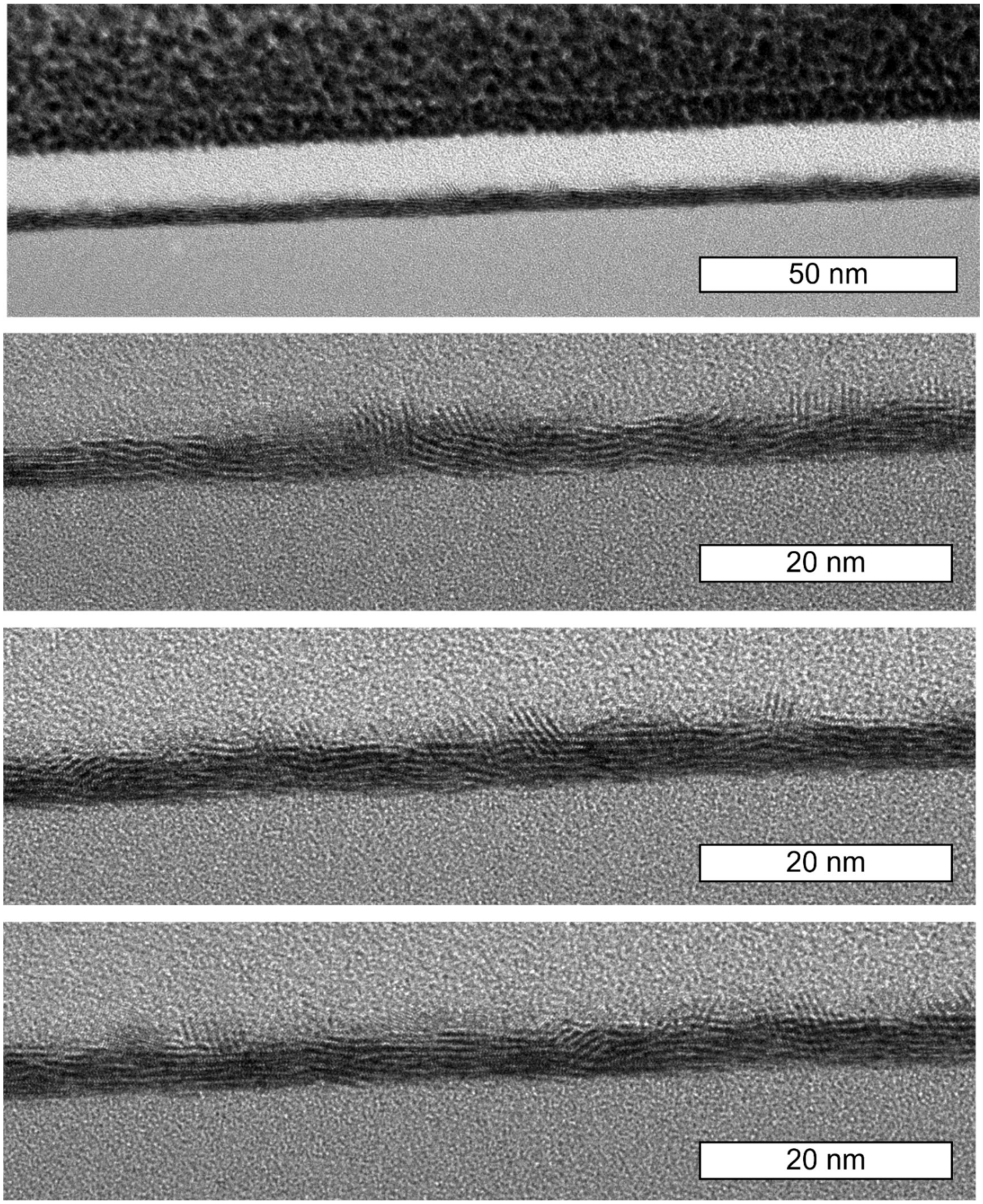

Figure S24. Additional cross-sectional TEM images of the A $B_{0.20}$ C_{Ar} sample shown in Figure 7c showing different magnifications and locations.

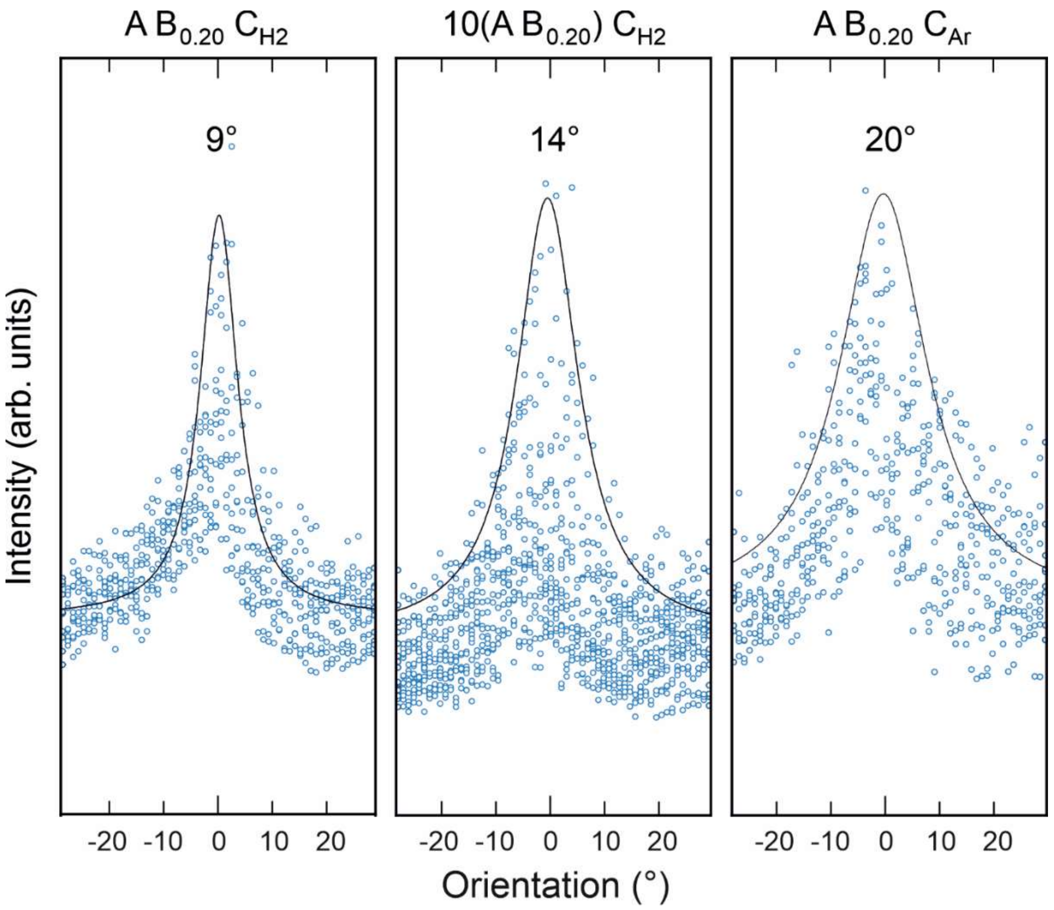

Figure S25. Aggregate analysis of MoS_2 basal plane orientation from cross-sectional TEM images (11 images for A $B_{0.20}$ C_{H2}, 20 for 10(A $B_{0.20}$) C_{H2}, and 9 for A $B_{0.20}$ C_{Ar}). FWHM of a Lorentzian peak set to contain the majority of the crystallites and centered at 0° (parallel to substrate) is shown.

The orientation analysis displayed in Figure S25 is described in the following. Between 9 and 20 TEM cross-sectional images per sample (magnification 250 000x) were analyzed. After cropping by 10% to remove the scale bar, a 2D Fourier transform of the image was taken using a fast Fourier transform (FFT) algorithm. A ring mask corresponding to the interlayer spacing of MoS_2 (0.6 nm, i.e., 100 px at the given magnification and image resolution) was applied to the FFT image and the intensity along the ring was analyzed using a set of six one degree wide wedge masks (at 60° repetition corresponding to the crystal symmetry of MoS_2) intersecting with the ring mask that were then rotated in one degree steps. The intensity at the intersection reaching above the background corresponds to crystallite(s) oriented at this angle. The tilt of the sample in the images was corrected for by setting the center of the orientation distribution to 0 degrees, i.e. parallel to the substrate surface. The FWHM of the distributions was estimated by placing a Lorentzian peak encompassing the majority of the crystallites in the sample. As this is not a result of a rigorous fitting procedure, as the orientation distribution might not be Lorentzian, and as it is difficult to separate grains at a large angle with respect to the substrate plane from the background (noise), the distribution widths are not to be taken to strictly correspond to grain orientation distribution.

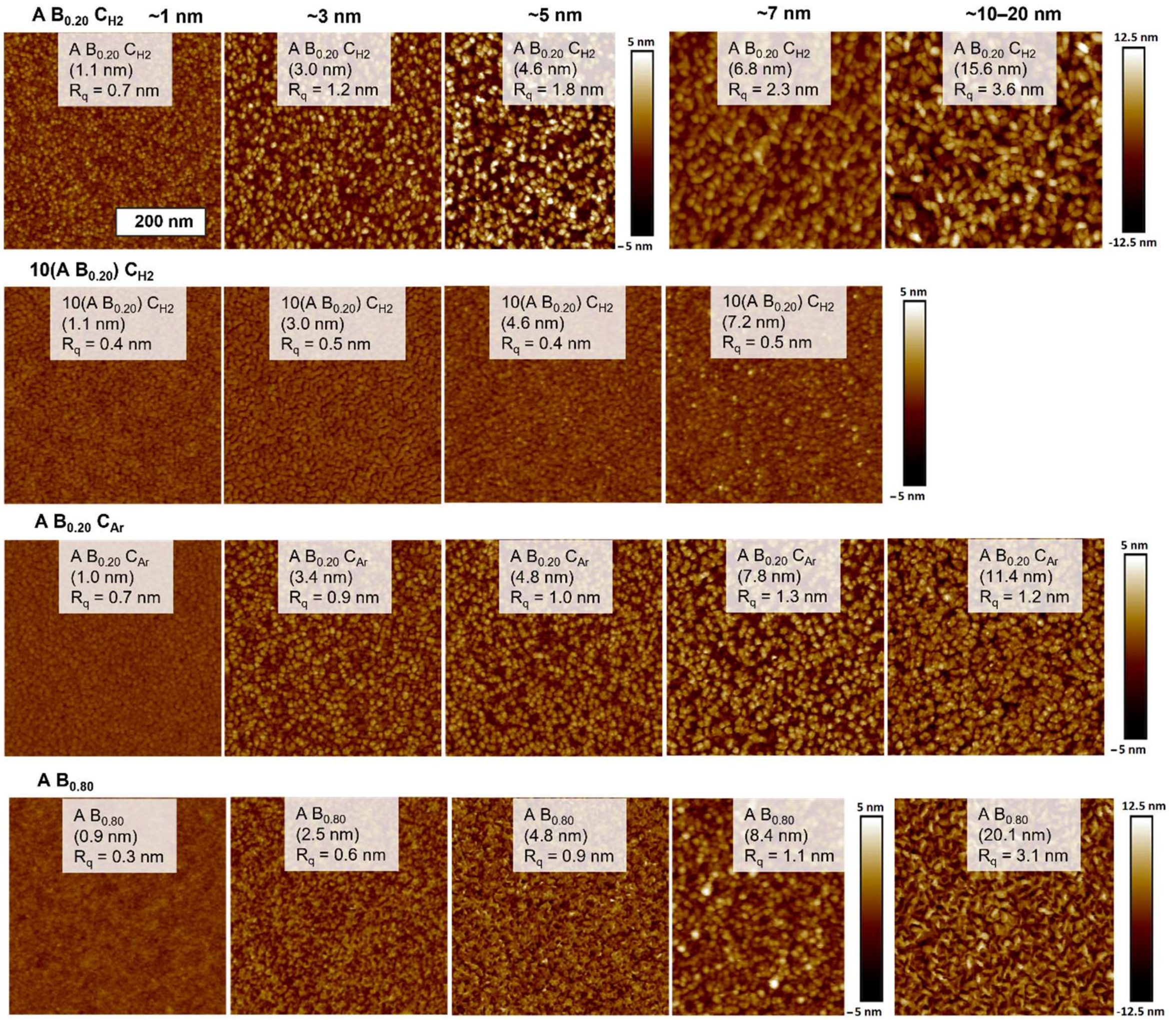

Figure S26. AFM images and roughness (R_q) values of films of different thicknesses deposited using different processes at 150 °C. Film thicknesses determined by SE are shown in parenthesis.

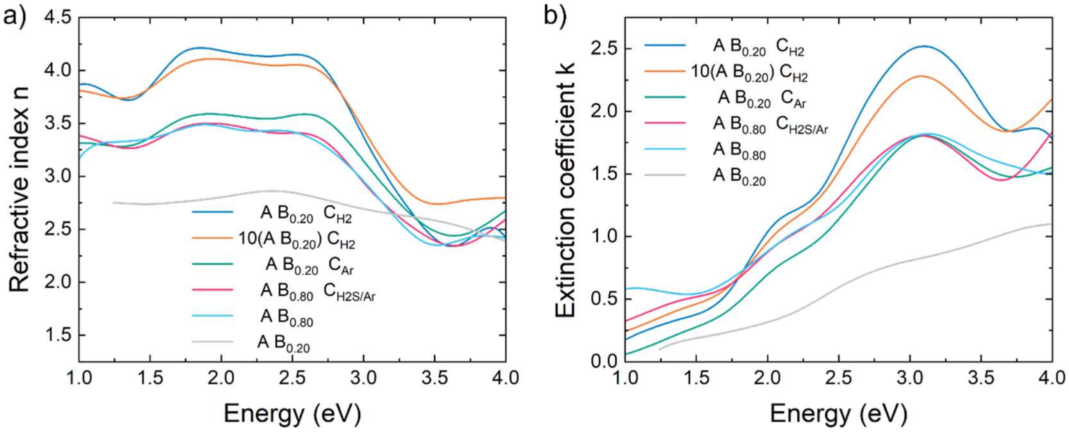

Figure S27. Optical constants: a) n and b) k of approximately 5 nm thick MoS_x films deposited using different processes as extracted from *in situ* SE data using a B-spline model.

S3.2: Film composition

Table S7. Results of XPS peak deconvolution for samples deposited using different AB and ABC processes. The binding energies (BEs), relative intensities (areas), and FWHMs of different components are listed for Mo 3d and S 2p regions. The amount the BEs were shifted to place the C-C/C-H C 1s component of adventitious carbon at 284.8 eV is also indicated (thus, the raw BE values can be calculated by deducting this shift from the BEs listed in the table). The films were deposited using 140 ALD cycles at 150 °C.

Process	S/Mo	Mo $3d_{5/2}$			S $2p_{3/2}$		
		Mo^{4+} (MoS_x)	Mo^{5+} (MoO_yS_x)	Mo^{6+} (MoO_3)	S^{2-} (MoS_x)	S_2^{2-} (MoS_{2+x})	BE shift to C1s ref.
A $B_{0.20}$	3.52	229.5 eV<br>82%<br>1.2 eV	230.8 eV<br>12%<br>2.0 eV	232.6 eV<br>6%<br>2.0 eV	162.2 eV<br>42%<br>1.6 eV	163.5 eV<br>58%<br>1.3 eV	+0.4 eV
A $B_{0.20}$ C_{H2}	1.89	229.6 eV<br>89%<br>0.9 eV	231.5 eV<br>4%<br>1.3 eV	232.9 eV<br>8%<br>2.0 eV	162.4 eV<br>100%<br>0.8 eV	-	+0.5 eV
10(A $B_{0.20}$) C_{H2}	1.93	229.6 eV<br>86%<br>0.9 eV	231.4 eV<br>6%<br>1.5 eV	232.9 eV<br>9%<br>2.0 eV	162.5 eV<br>100%<br>0.8 eV	-	+0.5 eV
A $B_{0.20}$ C_{Ar}	2.32	229.5 eV<br>90%<br>0.9 eV	231.4 eV<br>5%<br>1.4 eV	232.8 eV<br>5%<br>1.8 eV	162.4 eV<br>86%<br>0.8 eV	163.4 eV<br>14%<br>1.3 eV	+0.6 eV
A $B_{0.80}$	1.92	229.6 eV<br>86%<br>1.0 eV	231.5 eV<br>4%<br>1.3 eV	232.9 eV<br>10%<br>1.7 eV	162.4 eV<br>100%<br>0.9 eV	-	+0.5 eV

S3.3: Electrical properties

In this section, electrical properties of MoS_2 films deposited using different processes will be discussed in more detail including four-point-probe (FPP), Hall effect, and field-effect transistor (FET) measurements.

The resistivities measured by FPP as a function of film thickness are shown in Figure S28. An increase in resistivity with decreased thickness is especially pronounced below a thickness of approximately 5 nm, which is attributed to increased scattering at interfaces with substrate and air, as well as potentially decreasing grain size. The order between different processes A $B_{0.20}$ C_{Ar} > A $B_{0.20}$ C_{H2} ≈ 10(A $B_{0.20}$ C_{H2}) > A $B_{0.80}$ holds true for each thickness.

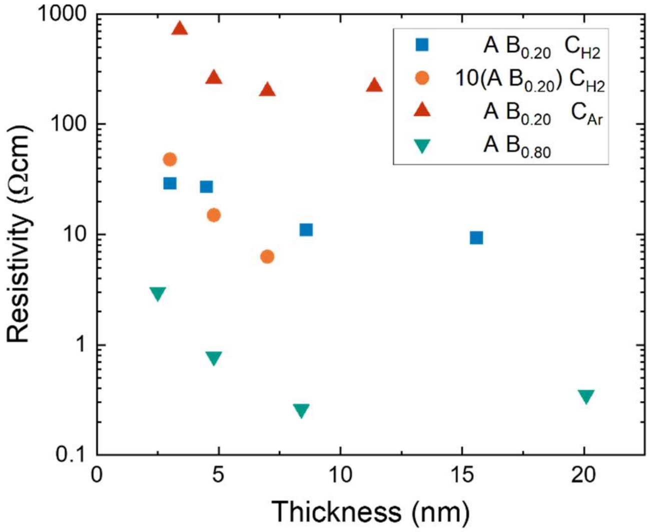

Figure S28. Four-point-probe resistivity of films deposited using different processes as a function of thickness. The resistivity of approximately 1 nm films exceeded the detection limit of the instrument (~10^9 Ohm, which translates to ~400 Ωcm for 1 nm thickness).

Hall effect measurements were performed in AC measurement scheme as this allows for greater sensitivity of low-mobility samples compared to conventional DC measurements.[20] We have discussed some of the advantages and limitations of this technique to PEALD MoS_2 samples previously.[5] The relatively low mobilities result in low Hall voltages, which gives rise to large statistical uncertainty. The measurements were repeated multiple times and different excitation currents were tested to ensure the extracted Hall voltages and thus the Hall mobilities and carrier concentrations are valid.

The A $B_{0.80}$ process results in very high carrier densities and low mobilities of approximately 10^{21} cm^{-3} and 0.01 $cm^2V^{-1}s^{-1}$ (Table S8). We believe this to be due to the inadvertent doping from approximately 20 at.% H incorporated into the films as discussed previously.[5] The ABC processes introduced here result in lower H concentrations. In line with the hypothesis of hydrogen acting as a dopant, the H_2 and Ar plasma based ABC processes exhibited much lower carrier densities of ~1–3 × 10^{18} cm^{-3} and ~6 × 10^{16} cm^{-3}. The carrier density is, however, not linearly dependent on H concentration. Superlinear dependence of carrier concentration on dopant concentration was also observed for Al-doped MoS_2 studied by Vandalon et al.[21]

In addition to H concentration, the chemical environment of H is expected to play an important role on the electrical properties of the films. It is, however, extremely challenging to probe experimentally (see Ref. 5 for more detailed discussion). Density functional theory calculations have indicated that H may bind

to S vacancies, S atoms forming thiol (-SH) moieties, or interstitial sites in the Mo plane.[5] Stoichiometry, crystallinity and morphology and thus varying availability of basal plane and edge sites likely affect where H is located. Hydrogen located in different sites can changes its effect on electrical properties, such as doping efficiency and even n or p-type nature of the H dopant.[5]

Besides hydrogen, S vacancies and excess S, the latter observed especially for the A $B_{0.20}$ C_{Ar} process, are likely to affect electrical properties. Other impurities including O and N may also play a role. However, the concentration of O and N is similar for both H_2 and Ar plasma based ABC processes (Table 1), while their carrier densities differ by more than an order of magnitude. This suggests that O and N are unlike to be the major reasons behind changes in electrical properties.

The Hall mobility of the ABC films was also improved by an order of magnitude compared to the A $B_{0.80}$ process, that is approximately 0.3 $cm^2V^{-1}s^{-1}$. The increased carrier mobility is attributed to decreased charge carrier scattering. The similar carrier mobilities of the three ABC processes may be understood by considering trends in three scattering mechanisms. The largest grain size of the A $B_{0.20}$ C_{H2} process (lowest grain boundary scattering) is balanced by presence of fins (increased surface/interface scattering) and moderate H impurity content (impurity scattering). For the A $B_{0.20}$ C_{Ar} process, on the other hand, the smallest grain size results in a higher degree of impurity scattering, which is balanced by the lowest degree of impurity scattering and modest surface scattering. In contrast, the $AB_{0.80}$ process has strong contributions from all three scattering sources – impurities, grain boundaries, and surface – resulting in inferior carrier mobility compared to the ABC processes developed in this work.

Table S8. Results of AC Hall effect measurements in tabulated form. Film thickness (SE) and H concentration (ERD) are included for reference. Note that the indicated resistivity was measured during the Hall measurements. The uncertainties represent standard deviation of 7–15 measurements repeated without remounting the sample. Asterisk (*) in the carrier type indicates that the noted carrier type was found in the majority of the repetitions, but the opposite type also occurred in some repetitions due to low signal-to-noise ratio.

Process	Thickness (nm)	H conc. (at.%)	ρ (Ωcm)	Hall mobility ($cm^2V^{-1}s^{-1}$)	Carrier concentration (cm^{-3})	Majority carrier type
A $B_{0.20}$ C_{H2}	16	9 ± 1	25	0.25 ± 0.15	$1.3 \pm 0.7 \times 10^{18}$	n*
10(A $B_{0.20}$) C_{H2}	7.0		10	0.30 ± 0.13	$2.6 \pm 1.4 \times 10^{18}$	n*
A $B_{0.20}$ C_{Ar}	55	2.3 ± 0.2	250	0.36 ± 0.17	$6 \pm 3 \times 10^{16}$	n*
A $B_{0.80}$	20	22 ± 2	0.28	0.015 ± 0.003	$1.6 \pm 0.5 \times 10^{21}$	p

S3.4 Summary

Table S9. Summary of selected film properties produced by different processes. Properties of A $B_{0.20}$, A $B_{0.65}$, and A $B_{0.80}$ processes are shown for comparison (partially from Ref.[5]). Unless otherwise noted, approximately 10–20 nm thick films deposited using 140 ALD cycles were analyzed. n/a = not applicable, - = not measured, * = ~15 nm. t_{ML} refers to the time required to grow thickness equivalent to one MoS_2 monolayer (0.65 nm).

	A $B_{0.20}$	A $B_{0.20}$ C_{H2}	10(A $B_{0.20}$) C_{H2}	A $B_{0.20}$ C_{Ar}	A $B_{0.65}$	A $B_{0.80}$
			Composition (S/Mo by XPS, RBS; H by ERD)			
S/Mo: XPS	3.5	1.9	1.9	2.3	2.1	1.9
RBS	3.7	2.0	-	2.3	2.1	1.8
H conc. (at.%)	6	9	-	2	12	22
			Growth (SE)			
GPC (Å)	0.7	1.1	0.4	0.8	1.0	1.3
t_{ML} (min)	n/a	7:50	14:40	15:30	5:10	4:20
			Crystallinity (Raman and AFM; ~5 nm films)			
Raman E^1_{2g} / A_{1g} FWHM(°)	n/a	7.0 / 7.8	9.6 / 9.7	8.0 / 8.7	9.4 / 8.9 *	8.1 / 8.5
XRD (0002) FWHM (°)	n/a	2.1	1.4	~7	-	3.1
			Morphology (AFM; ~3 and 7 nm films)			
AFM R_q ~3 nm	-	1.2	0.5	0.9	-	0.6
~7 nm (nm)	-	2.3	0.5	1.3	0.9 *	1.1
			Electrical properties (ρ by FPP; n, μ by Hall)			
ρ (Ωcm)	>4000	9.3	8.7	220	0.45	0.35
n (cm^{-3})	n/a	~1 × 10^{18}	~3 × 10^{18}	~6 × 10^{16}	~2 × 10^{21}	~1 × 10^{21}
μ ($cm^2V^{-1}s^{-1}$)		~0.3	~0.3	~0.3	~0.004	~0.02
			HER characteristics (7 nm films)			
$η_{10}$ (mV)	-	573	484	406	-	-
Tafel slope (mV/dec)	-	165	115	123	-	-
C_{dl} (μF/cm^2)	-	18	36	135	-	-

S3.5 Literature comparison

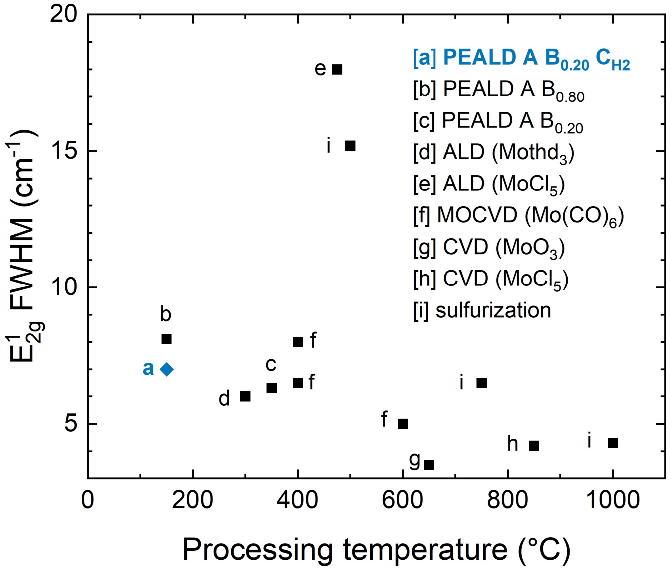

Figure S29. Comparison of Raman E^1_{2g} peak FWHM of the A $B_{0.20}$ C_{H2} process (**a**) to literature as a benchmark of crystallinity. For references and other material and process characteristics, see Table S10. Smaller FWHM indicates better crystallinity. For comparison, (exfoliated) bulk crystals have a FWHM of 3–4 cm^{-1}.

Table S10. Comparison of different aspects of our ABC processes (**A $B_{0.20}$ C_{H2}** as an example) and film characteristics to other methods including temperature, film area and uniformity, thickness and its controllability, crystallinity (grain size and Raman peak width) and electronic properties (carrier mobility). σ = non-uniformity. ^{A} = post-deposition annealing, FL = few-layer, ML = monolayer, μ_H = Hall mobility, μ_{FET} = field-effect mobility, * = depends on the method used to make the precursor film.

Method	T (°C)	Area, non-uniformity	Thickness	Grain size (nm)	Raman E^1_{2g} FWHM (cm^{-1})	Carrier mobility (cm^{2}V^{-1}s^{-1})	Ref.
**PEALD ($AB_{0.20}$ C_{H2})**	**150**	**4", σ = 2%**	**FL, sub-ML control**	**~5–20**	**7.0-9.6**	**μ_H ≈ 0.3**	**This work**
PEALD ($AB_{0.80}$)	100<br>150	4", σ = 3%	FL, sub-ML control	~5–10	 8.1	μ_H ≈ 0.01<br>μ_H ≈ 0.02	5<br>This
PEALD ($AB_{0.20}$)	350	4"	FL, sub-ML control		6.3		This, 22
ALD ($MoCl_5$ + $S(SiMe_3)_2$)	350 (900)^A	4", σ ≈ 5 %	FL, sub-ML control	~10		μ_{FET} ≈ 0.6–6	23
ALD ($MoCl_5$ + H_2S)	475	6" , σ = ?	1-2 ML		18		24
ALD ($Mo(CO)_6$+SEt_2)	250 (450)^A	6", σ = 3 %	FL, sub-ML control	Up to 75		μ_{FET} ≈ 10.6	25
ALD ($Mothd_3$+H_2S)	300	5 x 5 cm	FL	~10–20	~6		26

MOCVD ($Mo(CO)_6$ + H_2S)	200	~cm	ML–FL	~10		-	[27]
	300	10 cm	ML			-	[28]
	400	8”, σ = ?		~10–100	~6.5	$\mu_{FET} \approx 0.47$	[29]
MOCVD ($Mo(CO)_6$+SEt_2)	550	4”, σ = ?	ML	~200–400		$\mu_{FET} \approx 29$	[30]
	400			~10	~8		[31]
	600			~30	~5		
CVD (MoO_3 + S)	750	<1 mm	-	~2000		$\mu_{FET} \approx 1.8$	[32]
	650	2 mm	-		3.5	$\mu_{FET} \approx 0.02$	[33]
	1050	1 cm^2, 99% bilayer	2 ML	~10–100		$\mu_{FET} \approx 122$	[34]
CVD ($MoCl_5$ + S)	850	5 cm	ML to FL		4.2	$\mu_{FET} \approx 0.03$	[35]
MoO_3 sulfurization	750	~cm^2*	2 or 4 ML		6.5		[36]
	750	1 cm^2*	ML–FL*	10–30		$\mu_{FET} \approx 0.04$	[37]
Plasma sulfurization	100-200	4”, σ = ?	FL	~5			[38]
	300	4”, σ = ?	FL	~10	~20		[39]
$(NH_4)_2MoS_4$ spin coat + anneal	450	4”, σ = ?	FL	~10		$\mu_{FET} \approx 14$	[40]
Exfoliation + printing	200-300	4”, non-uniform	~10 nm thick	~1000		$\mu_{FET} \approx 10$	[41]
Exfoliation + printing	70	<1 cm^2	100s of nm thick	~350		$\mu_{FET} \approx 10$	[42]
Exfoliation + printing	~25	2 cm	100s of nm thick	~10		$\mu_{FET} \approx 0.02$	[43]

S4: Application in HER electrocatalysis

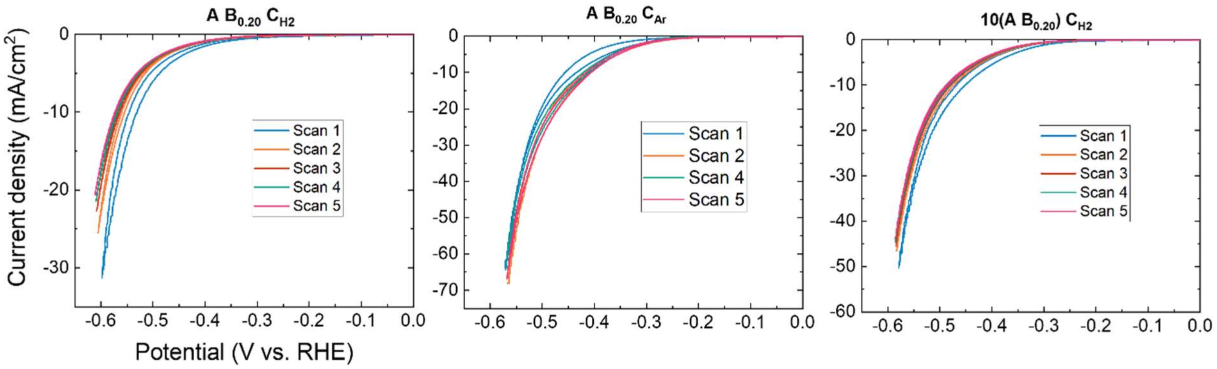

Figure S30. Five first CV scans of MoS_2 HER catalysts deposited using three different ABC processes.

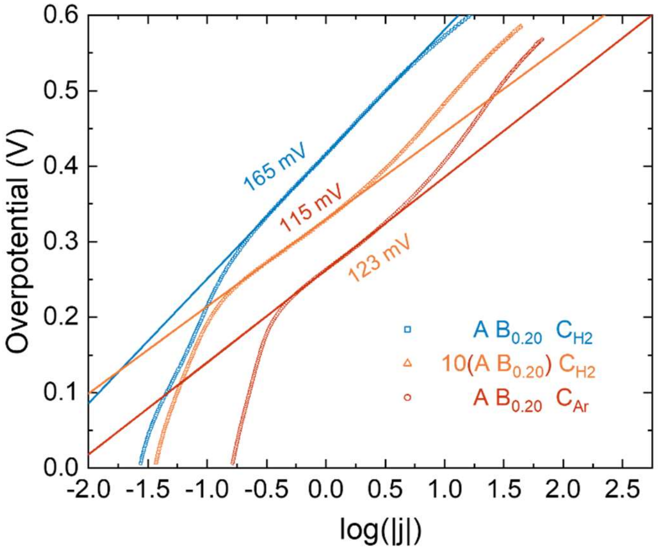

Figure S31. Tafel plots of MoS_2 HER catalysts deposited using three different ABC processes.

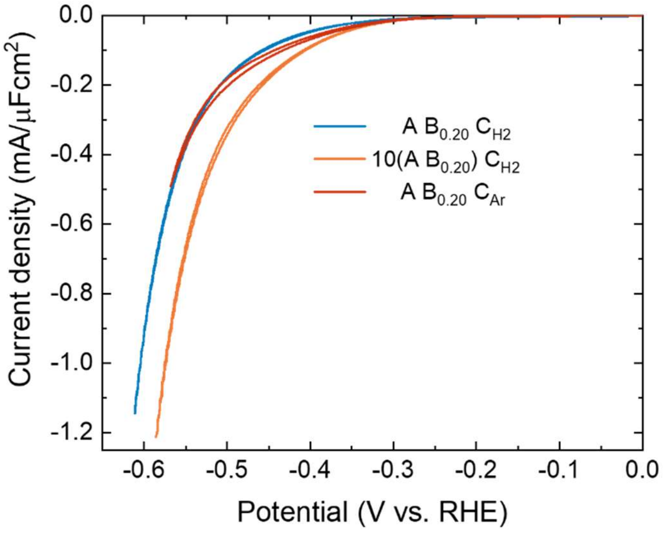

Figure S32. CV scans with current density divided by C_{dl} for MoS_2 HER catalysts deposited using three different ABC processes.

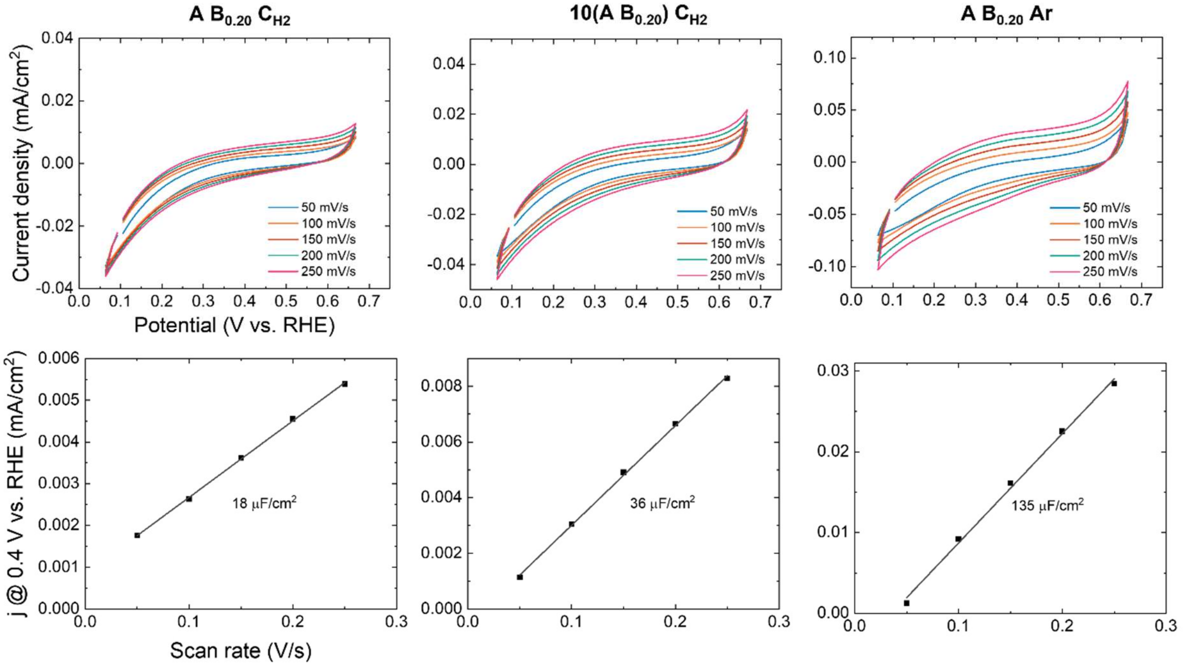

Figure S33. CV scans in non-Faradaic region at different scan rates (top row) and plots of current density at 0.4 V vs. RHE (anodic scan) versus scan rate and C_{dl} extracted from slope of a linear fit to the data (bottom row) for MoS_2 HER catalysts deposited using three different ABC processes. The potential and scan direction were chosen to minimize the non-Faradaic contributions apparent near the extrema of the scanning range. The good linear fits to the data confirm appropriateness of the chosen potential.

S5: Experiments on additional ABC processes

S5.1: A $B_{0.20}$ $C_{H2/Ar}$ process using mixed H_2/Ar plasma

In addition to pure H_2 and Ar plasmas, using a mixed H_2/Ar plasma added as a C step to A $B_{0.20}$ process was explored. The motivation stems from potential synergistic effects of chemical effects of H_2 plasma (H radicals) and physical effects of Ar plasma (Ar^+ ions). Besides exposure to both species, ions containing both Ar and H, such as ArH^+, are formed in such mixed plasmas.[12] Such ions carry chemically reactive species absent in Ar plasma and allow for effective energy transfer in collisions with film, which is an issue with the light ions such as H_3^+ formed in a pure H_2 plasma. The amount of H_2 is quantified as H_2 fraction as defined below (not to be confused with the H_2 flow ratio for the B step):

$$\mathrm{H_2\ fraction} = \frac{\mathrm{H_2\ flow\ (sccm)}}{\mathrm{H_2\ flow\ (sccm)+Ar\ flow\ (sscm)}}$$

A pure Ar plasma at a pressure of 6 mTorr and exposure time of 20 s had a limited capability to remove S, transforming $MoS_{3.5}$ to $MoS_{3.0}$ (Ar plasma is more efficient at this at lower pressures as discussed in Section S2.4). Adding a H_2 fraction as low as 0.04 notably improved the efficiency of S removal, resulting in $MoS_{2.2}$ stoichiometry (Table S11, Figure S34). Between H_2 fractions of 0.2 and 1, close to stoichiometric MoS_2 films were obtained. Raman spectroscopy showed that the films were crystalline, with no clear changes in the Raman spectra within this H_2 fraction range (Figure S35a). A trend of decreasing GPC with increasing H_2 fraction was observed (Figure S35b). SEM indicated that the 10–15 nm thick MoS_2 films grown using H_2 fractions of 0.04–1 were all very rough with a high density of fins (not shown). We also observed resistivity to increase with increasing H_2 fraction, the origin of which is not currently clear.

In summary, we did not observe clear benefits in using a mixed H_2/Ar plasma (A $B_{0.20}$ $C_{H2/Ar}$) instead of pure H_2 plasma (A $B_{0.20}$ C_{H2}), and therefore chose to use pure H_2 as well as Ar plasmas throughout the study.

Table S11. Summary of the effect of H_2/Ar flow ratio on film growth and properties in A $B_{0.20}$ $C_{H2/Ar}$ process. GPC was determined by dividing SE thickness by the number of ALD cycles (140), resistivity by combining sheet resistance measured by FPP and SE thickness (detection limit (dl) of ~10^9 Ohm corresponds to ~4000 Ωcm for 10 nm thick films), crystallinity by Raman spectroscopy, and S/Mo ratio by XPS

Exposure time (s)	H_2 fraction	Pressure (mTorr)	ICP power (W)	GPC (Å)	ρ (Ωcm)	S/Mo ratio
**-** (no C step)	-	-	-	0.67	>dl	3.54
			Effect of H_2/Ar ratio			
20	1	20	100	1.11	9.3	1.89
20	0.8	6	100	1.04	5.7	1.97
20	0.5	6	100	1.16	3.3	1.96
20	0.2	6	100	1.24	2.9	2.02
20	0.04	6	100	0.96	1.0	2.17
20	0	6	100	0.96	78	2.97

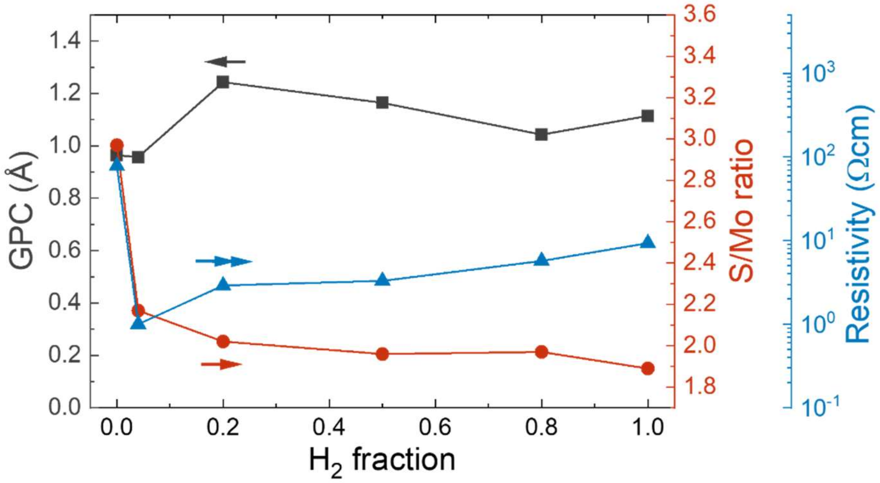

Figure S34. Effect of H_2 fraction in A $B_{0.20}$ $C_{H2/Ar}$ process on GPC (SE), S/Mo ratio (XPS), and resistivity (FPP). The films were deposited using 140 ALD cycles at 150 °C.

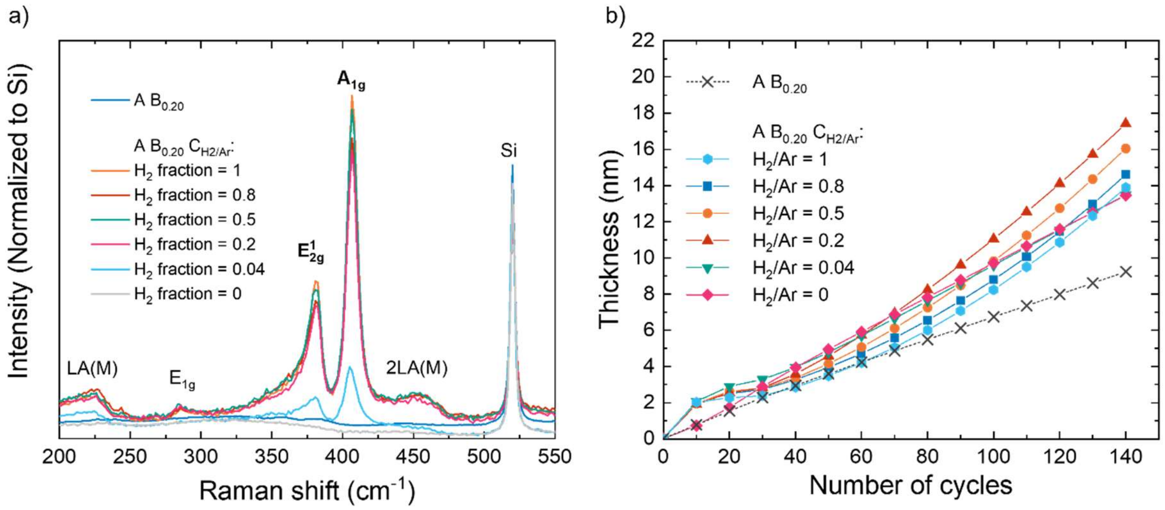

Figure S35. a) Raman spectra and b) *in situ* SE thicknesses of MoS_x films deposited using different H_2 fractions in A $B_{0.20}$ $C_{H2/Ar}$ process. The films were deposited using 140 ALD cycles at 150 °C.

S5.2: A $B_{0.80}$ $C_{H2S/Ar}$ process

All of the ABC processes described in the main text and so far in the Supporting Information used the C step to remove excess sulfur deposited by the A $B_{0.20}$ process. ABC processes can also be devised using different H_2 flow ratios in the B step. In the case of A $B_{0.80}$ $C_{H2S/Ar}$ process, we added a H_2S/Ar plasma C step to an A $B_{0.80}$ process that deposits slightly sulfur-deficient $MoS_{1.9}$ films with a very rough morphology. Based on our previous study,[5] we hypothesized that the A $B_{0.80}$ process results in sulfur-deficient (and/or hydrogen terminated) MoS_2 fins that grow rapidly and thus result in a rough morphology – a process that could perhaps be prevented by passivating the fins using a H_2S-based plasma C step.

Indeed, our initial experiments showed that adding a H_2S/Ar plasma step to the A $B_{0.80}$ process had a dramatic effect on morphology, resulting in much smoother films and an associated strong decrease in GPC (1.4→1.0 Å), whereas only a rather modest increase of S/Mo ratio from 1.9 to 2.3 was observed. Adding a H_2S gas step instead, even at the highest tested operating pressure of 200 mTorr, had no clear effect on film growth and properties (Table S12), showing that plasma activation of H_2S was needed. In all cases, 10 sccm of H_2S mixed with 40 sccm of Ar was used with 100 W ICP power.

We first varied the pressure during the H_2S/Ar plasma step from 6 to 200 mTorr, keeping the exposure time constant at 20 s. Increasing pressure slightly decreased GPC and increased S/Mo ratio and resistivity (Table S12, Figure S36a). The intensity of MoS_2 Raman modes was similar at 6 and 35 mTorr but clearly lower at 200 mTorr, suggesting decreased crystallinity at the highest pressure (Figure S37a). The lowest pressure of 6 mTorr was chosen for further experiments.

Varying the exposure time at 6 mTorr from 5 to 60 s resulted in only minor changes in film properties and growth, proving that the process was surface limited and saturating (Figures S36b and S37b). Linear growth was observed by *in situ* SE regardless of the H_2S/Ar plasma conditions by SE, which is in line with the smooth morphology suggested by SEM (not shown). A 20 s exposure at 6 mTorr was chosen as the standard condition as that also corresponded to the B step plasma conditions.

Films deposited under the optimized conditions underwent further characterization for their composition, morphology, and electrical properties. RBS measurements confirmed the XPS stoichiometries, yielding a S/Mo ratio of 2.20±0.08 (2.26 by XPS). The H concentration analyzed by ERD was 11.2±0.8 at.%, i.e., lower compared to the A $B_{0.80}$ process (22±2 at.%) but higher than the A $B_{0.20}$ C_{H2} (9.0±0.7 at.%) and A $B_{0.20}$ C_{Ar} (2.3±0.2 at.%) processes. Similarly, the O content of 2.5±0.7 at.% lied between the A $B_{0.20}$ C_y and A $B_{0.80}$ processes, while the N content of 1.0±0.3 at.% was the highest among the processes together with the A $B_{0.80}$ process (see the main article for more detailed results). Fitting of the core-level X-ray photoelectron spectra was rather similar to the other ABC processes (see Section S3.2), the most obvious difference being a negative 0.3–0.4 eV shift of Mo 3d and S 2p BEs as well as a slightly larger FWHM of the Mo^{4+} and S^{2-} components (1.1–1.2 vs 0.8–0.9 eV) for the A $B_{0.80}$ $C_{H2S/Ar}$ process. The larger FWHMs may suggest a more heterogeneous and/or disordered Mo and S chemical environment for the A $B_{0.80}$ $C_{H2S/Ar}$ proces.

Decreased roughness of the films deposited using the A $B_{0.80}$ $C_{H2S/Ar}$ process compared to the A $B_{0.80}$ process is shown in Figure S38. However, the roughness of the A $B_{0.80}$ $C_{H2S/Ar}$ processes was still higher compared to the 10(A $B_{0.20}$) C_{H2} process (see the main article). The resistivity data suggest that addition of the H_2S/Ar C step has only a minor effect on the electrical properties. AC Hall effect measurements confirmed that the carrier concentration (~10^{21} cm^{-3}) and mobility (~0.01 $cm^2V^{-1}s^{-1}$) were indeed comparable to the A $B_{0.80}$ process. Therefore, we chose to focus on the A $B_{0.20}$ C_y processes.

Table S12. Summary of the effect of H_2S/Ar plasma conditions (pressure and exposure time) on film growth and properties in A $B_{0.20}$ $C_{H2S/Ar}$ process. The condition deemed optimized is shown in **bold**. GPC was determined by dividing SE thickness by the number of ALD cycles (140), resistivity by combining sheet resistance measured by FPP and SE thickness, and S/Mo ratio by XPS

Exposure time (s)	Pressure (mTorr)	ICP power (W)	GPC (Å)	ρ (Ωcm)	S/Mo ratio
- (no C step)	-	-	1.44	0.35	1.89
20	200	0 (gas)	1.59	0.21	1.88
		Effect of pressure			
**20**	**6**	**100**	**0.96**	**0.34**	**2.26**
20	35	100	0.93	0.38	2.31
20	200	100	0.86	0.44	2.41
		Effect of exposure time			
5	6	100	1.04	0.34	2.21
**20**	**6**	**100**	**0.96**	**0.34**	**2.26**
60	6	100	1.00	0.57	2.30

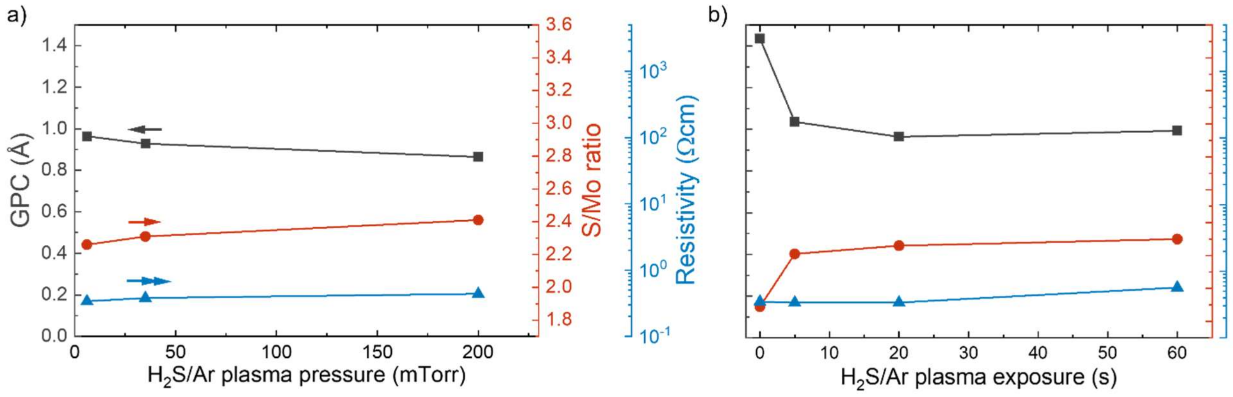

Figure S36. Effect of a) pressure (20 s exposure) and b) exposure time (6 mTorr pressure) during the H_2S/Ar C step in A $B_{0.20}$ $C_{H2S/Ar}$ process on GPC (SE), S/Mo ratio (XPS), and resistivity (FPP). The films were deposited using 140 ALD cycles at 150 °C. The same scale applies to both panels

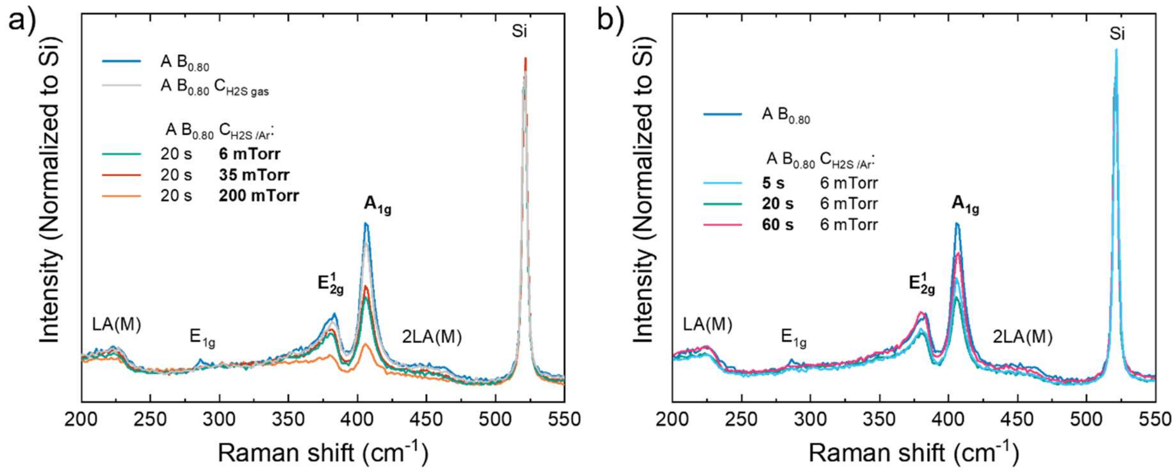

Figure S37. Raman spectra of MoS_x films deposited using different a) pressures and b) exposure times in the H_2S/Ar plasma C step (A $B_{0.80}$ $C_{H2S/Ar}$ process). For comparison, also A $B_{0.20}$ and A$B_{0.20}$ $C_{H2S\ gas}$ processes are shown. The films were deposited using 140 ALD cycles at 150 °C.

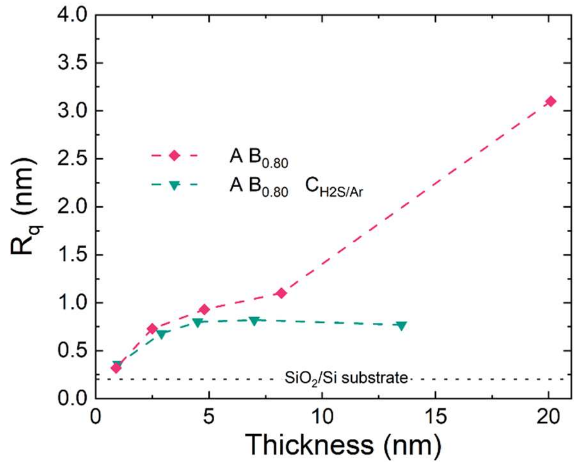

Figure S38. AFM-derived roughness evolution of the A $B_{0.80}$ $C_{H2S/Ar}$ and A $B_{0.80}$ processes.

S5.3: Trials of A $B_{0.80}$ C_{H2} process

We also explored adding a H_2 plasma C step to A $B_{0.80}$ process motivated by preceding work on WS_2, where a H_2 plasma C step was found to decrease fin density, albeit at a higher temperature of 300 °C and different plasma conditions.[14] We used H_2 plasma conditions optimized for the A $B_{0.20}$ C_{H2} process.

Surprisingly, A $B_{0.20}$ C_{H2} process resulted in highly sulfur-deficient $MoS_{1.3}$ films with a low resistivity below 10 mΩcm (Table S13). Analysis of Mo 3d core level X-ray photoelectron spectra (not shown) revealed a considerable part of Mo to be present at binding energies lower than expected for MoS_2. Raman spectroscopy showed presence of MoS_2 peaks, albeit at notably reduced intensity compared to the A $B_{0.80}$ process (Figure S39a). The GPC after 140 ALD cycled decreased to half (Figure S39b), together with a large decrease in fin density compared to the A $B_{0.80}$ process. These observations – in particular XPS and resistivity – suggest the films to consist of a mixture of Mo and MoS_2 phases, similar to what we observed previously using very high H_2 flow ratios in A $B_{0.94}$ process.[5]

The observed etching of S from $MoS_{1.9}$ starting stoichiometry is quite surprising considering that in our A $B_{0.20}$ C_{H2} process the stoichiometry changed very little even after an extended 60 s plasma exposure. Thus, the films deposited using the A $B_{0.20}$ C_{H2} process can be concluded to be more stable against etching of S compared to the A $B_{0.80}$ process, which may be linked to improved crystallinity and decreased H concentration in the films produced by the A $B_{0.20}$ C_{H2} process. For comparison, in the aforementioned study of PEALD WS_2, adding a 50 s H_2 plasma C step at 500 W ICP power and 15 mTorr pressure (that is, more aggressive than the condition used here) resulted in a stoichiometry change from $WS_{2.0}$ to $WS_{1.6}$.[14]

Table S13. Summary of film properties obtained in A $B_{0.80}$ C_{H2} process. GPC was determined by dividing SE thickness by the number of ALD cycles (140), resistivity by combining sheet resistance measured by FPP and SE thickness, crystallinity by Raman spectroscopy, and S/Mo ratio by XPS

Exposure time (s)	Pressure (mTorr)	ICP power (W)	GPC (Å)	ρ (Ωcm)	S/Mo ratio
- (no C step)	-	-	1.44	0.35	1.89
20	20	100	0.70	0.007	1.30

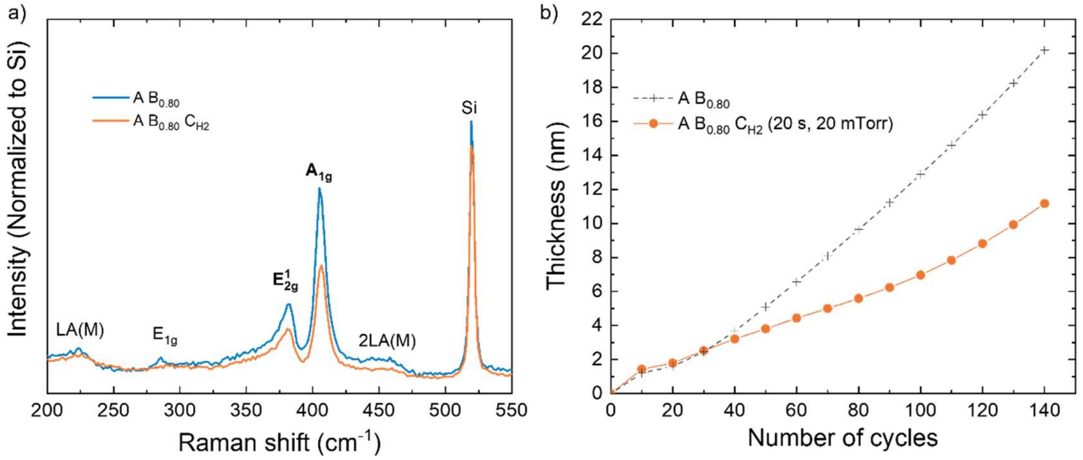

Figure S39. a) Raman spectra and b) *in situ* SE thicknesses of MoS_x films deposited using A $B_{0.80}$ and A $B_{0.80}$ C_{H2} processes at 150 °C (140 ALD cycles).

S5.4: Trials of A $B_{0.80}$ C_{Ar} process

We also evaluated adding an Ar plasma C step to the A $B_{0.80}$ process. A similar approach was studied for PEALD WS_2 at 300 °C, where an optimized Ar plasma C step decreased formation of out-of-plane oriented fins by 80%.[14] The low hydrogen concentration observed in the films deposited using the A $B_{0.20}$ C_{Ar} process also motivated these experiments, as that suggested that Ar plasma can remove hydrogen – which is present at ~20 at.% in the crystalline films deposited using the A $B_{0.80}$ process.

We tested a few different Ar plasma conditions based on optimization for the A $B_{0.20}$ C_{Ar} process (see Section S2.4). A 20 s exposure at 6 mTorr pressure approximately doubled the resistivity with little effect on S/Mo ratio, which may indicate removal of some of the H (Table S14). Increasing exposure time to 60 s and decreasing pressure to 1 mTorr, which should considerably increase the dose of Ar ions, further increased resistivity and decreased GPC by 25% without a clear effect on stoichiometry or crystallinity (Figure S40a). AFM showed that there was also no clear effect on fin density, which remained very high (Figure S40b). The effect on H concentration was not analyzed. Finally, use of an RF bias to increase ion energy by approximately 40 V was examined. The resulting film had a very low S/Mo ratio of 0.5 and was highly conductive, suggesting that such Ar^+ ions were too energetic and removed S from the $MoS_{1.9}$ films. Thus, although adding an Ar plasma C step to A $B_{0.80}$ process using suitable plasma conditions may allow for a decrease in hydrogen concentration and/or roughness, such conditions were not identified here.

Table S14. Summary of film properties obtained in A $B_{0.80}$ C_{Ar} process. GPC was determined by dividing SE thickness by the number of ALD cycles (140), resistivity by combining sheet resistance measured by FPP and SE thickness, crystallinity by Raman spectroscopy, and S/Mo ratio by XPS

Exposure time (s)	Pressure (mTorr)	ICP power (W)	RF bias (V) and power	GPC (Å)	ρ (Ωcm)	S/Mo ratio
**-** (no C step)	-	-	-	1.44	0.35	1.89
**20**	6	100	-	1.42	0.74	1.91
60	~1	100	-	1.09	3.9	1.90
20	6	100	40 (5 W)	0.57	0.0006	0.5

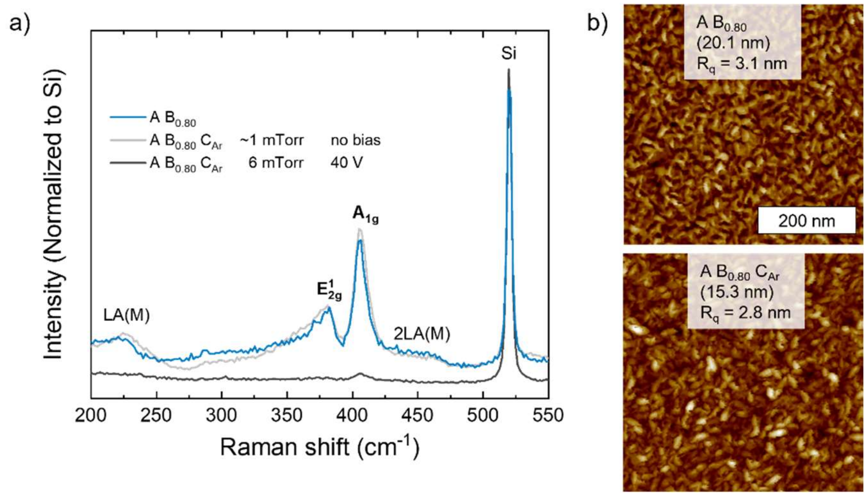

Figure S40. a) Raman spectra and b) AFM images and roughnesses (R_q) of MoS_x films deposited using A $B_{0.80}$, A $B_{0.80}$ C_{Ar} and A $B_{0.80}$ $C_{Ar\ bias}$ processes at 150 °C (140 ALD cycles).

S6: References

(1) Morales-Guio, C. G.; Hu, X. Amorphous Molybdenum Sulfides as Hydrogen Evolution Catalysts. *Acc. Chem. Res.* **2014**, *47*, 2671–2681.

(2) Mabayoje, O.; Liu, Y.; Wang, M.; Shoola, A.; Ebrahim, A. M.; Frenkel, A. I.; Mullins, C. B. Electrodeposition of MoS_x Hydrogen Evolution Catalysts from Sulfur-Rich Precursors. *ACS Appl. Mater. Interfaces* **2019**, *11*, 32879–32886.

(3) Tran, P. D.; Tran, T. V.; Orio, M.; Torelli, S.; Truong, Q. D.; Nayuki, K.; Sasaki, Y.; Chiam, S. Y.; Yi, R.; Honma, I.; Barber, J.; Artero, V. Coordination Polymer Structure and Revisited Hydrogen Evolution Catalytic Mechanism for Amorphous Molybdenum Sulfide. *Nat. Mater.* **2016**, *15*, 640–646.

(4) Balasubramanyam, S.; Merkx, M. J. M.; Verheijen, M. A.; Kessels, W. M. M.; Mackus, A. J. M.; Bol, A. A. Area-Selective Atomic Layer Deposition of Two-Dimensional WS_2 Nanolayers. *ACS Mater. Lett.* **2020**, *2*, 511–518.

(5) Mattinen, M.; Gity, F.; Coleman, E.; Vonk, J. F. A.; Verheijen, M. A.; Duffy, R.; Kessels, W. M. M.; Bol, A. A. Atomic Layer Deposition of Large-Area Polycrystalline Transition Metal Dichalcogenides from 100 °C through Control of Plasma Chemistry. *Chem. Mater.* **2022**, *34*, 7280–7292.

(6) Mattinen, M.; Leskelä, M.; Ritala, M. Atomic Layer Deposition of 2D Metal Dichalcogenides for Electronics, Catalysis, Energy Storage, and Beyond. *Adv. Mater. Interfaces* **2021**, *8*, 2001677.

(7) Wei, C.; Wu, W.; Li, H.; Lin, X.; Wu, T.; Zhang, Y.; Xu, Q.; Zhang, L.; Zhu, Y.; Yang, X.; Liu, Z.; Xu, Q. Atomic Plane-Vacancy Engineering of Transition-Metal Dichalcogenides with Enhanced Hydrogen Evolution Capability. *ACS Appl. Mater. Interfaces* **2019**, *11*, 25264–25270.

(8) Cheng, Y.; Song, H.; Wu, H.; Zhang, P.; Tang, Z.; Lu, S. Defects Enhance the Electrocatalytic Hydrogen Evolution Properties of MoS_2-Based Materials. *Chem. - An Asian J.* **2020**, *15*, 3123–3134.

(9) Das, S.; Swain, G.; Parida, K. A Concise Discussion on MoS_2 Basal Plane Activation toward the Ennoblement of Electrocatalytic HER Output. *Sustain. Energy Fuels* **2022**, *6*, 937–953.

(10) Profijt, H. B.; Kessels, W. M. M. Ion Bombardment during Plasma-Assisted Atomic Layer Deposition. *ECS Trans.* **2012**, *50 (13)*, 23–34.

(11) Faraz, T.; Arts, K.; Karwal, S.; Knoops, H. C. M.; Kessels, W. M. M. Energetic Ions during Plasma-Enhanced Atomic Layer Deposition and Their Role in Tailoring Material Properties. *Plasma Sources Sci. Technol.* **2019**, *28*, 024002.

(12) Sode, M.; Schwarz-Selinger, T.; Jacob, W. Ion Chemistry in H_2-Ar Low Temperature Plasmas. *J. Appl. Phys.* **2013**, *114*, 063302.

(13) Gudmundsson, J. T. Ion Energy Distribution in H_2/Ar Plasma in a Planar Inductive Discharge. *Plasma Sources Sci. Technol.* **1999**, *8*, 58–64.

(14) Balasubramanyam, S.; Bloodgood, M. A.; Ommeren, M. Van; Faraz, T.; Vandalon, V.; Kessels, W. M. M.; Verheijen, M. A.; Bol, A. A. Probing the Origin and Suppression of Vertically Oriented Nanostructures of 2D WS_2 Layers. *ACS Appl. Mater. Interfaces* **2020**, *12*, 3873–3885.

(15) Karwal, S.; Verheijen, M. A.; Arts, K.; Faraz, T.; Kessels, W. M. M.; Creatore, M. Plasma-Assisted ALD of Highly Conductive HfN_x: On the Effect of Energetic Ions on Film Microstructure. *Plasma*

Chem. Plasma Process. **2020**, *40*, 697–712.

(16) Knoops, H. C. M.; de Peuter, K.; Kessels, W. M. M. Redeposition in Plasma-Assisted Atomic Layer Deposition: Silicon Nitride Film Quality Ruled by the Gas Residence Time. *Appl. Phys. Lett.* **2015**, *107*, 014102.

(17) Profijt, H. B.; van de Sanden, M. C. M.; Kessels, W. M. M. Substrate-Biasing during Plasma-Assisted Atomic Layer Deposition to Tailor Metal-Oxide Thin Film Growth. *J. Vac. Sci. Technol. A.* **2013**, *31*, 01A106.

(18) Faraz, T.; Verstappen, Y. G. P.; Verheijen, M. A.; Chittock, N. J.; Lopez, J. E.; Heijdra, E.; Van Gennip, W. J. H.; Kessels, W. M. M.; Mackus, A. J. M. Precise Ion Energy Control with Tailored Waveform Biasing for Atomic Scale Processing. *J. Appl. Phys.* **2020**, *128*, 213301.

(19) Sode, M.; Jacob, W.; Schwarz-Selinger, T.; Kersten, H. Measurement and Modeling of Neutral, Radical, and Ion Densities in H_2-N_2-Ar Plasmas. *J. Appl. Phys.* **2015**, *117* 083303.

(20) Lindemuth, J. *Hall Effect Measurement Handbook*; Lake Shore Cryotronics, Westerville, OH.

Lindemuth, J.; Mizuta, S.-I. Hall Measurements on Low-Mobility Materials and High Resistivity Materials. In *Thin Film Solar Technology III*; 2011; Vol. 8110, p 81100I.

(21) Vandalon, V.; Verheijen, M. A.; Kessels, W. M. M.; Bol, A. A. Atomic Layer Deposition of Al-Doped MoS_2: Synthesizing a p-type 2D Semiconductor with Tunable Carrier Density. *ACS Appl. Nano Mater*. **2020**, *3*, 10200−10208.

(22) Sharma, A.; Verheijen, M. A.; Wu, L.; Karwal, S.; Vandalon, V.; Knoops, H. C. M.; Sundaram, R. S.; Hofmann, J. P.; Kessels, W. M. M.; Bol, A. A. Low-Temperature Plasma-Enhanced Atomic Layer Deposition of 2-D MoS_2: Large Area, Thickness Control and Tuneable Morphology. *Nanoscale* **2018**, *10*, 8615–8627.

(23) Liu, H.; Chen, L.; Zhu, H.; Sun, Q.; Ding, S.; Zhou, P.; Zhang, D. W. Atomic Layer Deposited 2D MoS_2 Atomic Crystals: From Material to Circuit. *Nano Res.* **2020**, *13*, 1644–1650.

(24) Valdivia, A.; Tweet, D. J.; Conley Jr., J. F. Atomic Layer Deposition of Two Dimensional MoS_2 on 150 Mm Substrates. *J. Vac. Sci. Technol. A* **2016**, *34*, 021515.

(25) Jeon, W.; Cho, Y.; Jo, S.; Ahn, J.-H.; Jeong, S.-J. Wafer-Scale Synthesis of Reliable High-Mobility Molybdenum Disulfide Thin Films via Inhibitor-Utilizing Atomic Layer Deposition. *Adv. Mater.* **2017**, *29*, 1703031.

(26) Mattinen, M.; Hatanpää, T.; Sarnet, T.; Mizohata, K.; Meinander, K.; King, P. J. ; Khriachtchev, L.; Räisänen, J.; Ritala, M.; Leskelä, M. Atomic Layer Deposition of Crystalline MoS_2 Thin Films: New Molybdenum Precursor for Low-Temperature Film Growth. *Adv. Mater. Interfaces* **2017**, *4*, 1700213.

(27) Zhao, Y.; Song, J. G.; Ryu, G. H.; Ko, K. Y.; Woo, W. J.; Kim, Y.; Kim, D.; Lim, J. H.; Lee, S.; Lee, Z.; Park, J.; Kim, H. Low-Temperature Synthesis of 2D MoS_2 on a Plastic Substrate for a Flexible Gas Sensor. *Nanoscale* **2018**, *10*, 9338–9345.

(28) Kim, Y.; Kang, S. K.; Oh, N. C.; Lee, H. D.; Lee, S. M.; Park, J.; Kim, H. Improved Sensitivity in Schottky Contacted Two-Dimensional MoS_2 Gas Sensor. *ACS Appl. Mater. Interfaces* **2019**, *11*, 38902–38909.

(29) Kim, T.; Mun, J.; Park, H.; Joung, D.; Diware, M.; Won, C.; Park, J.; Jeong, S.-H.; Kang, S.-W. Wafer-Scale Production of Highly Uniform Two-Dimensional MoS_2 by Metal-Organic Chemical Vapor Deposition. *Nanotechnology* **2017**, *28*, 18LT01.

(30) Kang, K.; Xie, S.; Huang, L.; Han, Y.; Huang, P. Y.; Mak, K. F.; Kim, C.-J.; Muller, D.; Park, J. High-Mobility Three-Atom-Thick Semiconducting Films with Wafer-Scale Homogeneity. *Nature* **2015**, *520*, 656–660.

(31) Simonson, N. A.; Nasr, J. R.; Subramanian, S.; Jariwala, B.; Zhao, R.; Das, S.; Robinson, J. A. Low-Temperature Metalorganic Chemical Vapor Deposition of Molybdenum Disulfide on Multicomponent Glass Substrates. *FlatChem* **2018**, *11*, 32–37.

(32) Kim, T. Y.; Amani, M.; Ahn, G. H.; Song, Y.; Javey, A.; Chung, S.; Lee, T. Electrical Properties of Synthesized Large-Area MoS_2 Field-Effect Transistors Fabricated with Inkjet-Printed Contacts. *ACS Nano* **2016**, *10*, 2819–2826.

(33) Lee, Y.-H.; Zhang, X.-Q.; Zhang, W.; Chang, M.-T.; Lin, C.-T.; Chang, K.-D.; Yu, Y.-C.; Wang, J. T.-W.; Chang, C.-S.; Li, L.-J.; Lin, T.-W. Synthesis of Large-Area MoS_2 Atomic Layers with Chemical Vapor Deposition. *Adv. Mater.* **2012**, *24*, 2320–2325.

(34) Liu, L.; Li, T.; Ma, L.; Li, W.; Gao, S.; Sun, W.; Dong, R.; Zou, X.; Fan, D.; Shao, L.; Gu, C.; Dai, N.; Yu, Z.; Chen, X.; Tu, X.; Nie, Y.; Wang, P.; Wang, J.; Shi, Y.; Wang, X. Uniform Nucleation and Epitaxy of Bilayer Molybdenum Disulfide on Sapphire. *Nature* **2022**, *605*, 69–75.

(35) Yu, Y.; Li, C.; Liu, Y.; Su, L.; Zhang, Y.; Cao, L. Controlled Scalable Synthesis of Uniform, High-Quality Monolayer and Few-Layer MoS_2 Films. *Sci. Rep.* **2013**, *3*, 1866.

(36) Vangelista, S.; Cinquanta, E.; Martella, C.; Alia, M.; Longo, M.; Lamperti, A.; Mantovan, R.; Basset, F. B.; Pezzoli, F.; Molle, A. Towards a Uniform and Large-Scale Deposition of MoS_2 Nanosheets via Sulfurization of Ultra-Thin Mo-Based Solid Films. *Nanotechnology* **2016**, *27*, 175703.

(37) Zhan, Y.; Liu, Z.; Najmaei, S.; Ajayan, P. M.; Lou, J. Large-Area Vapor-Phase Growth and Characterization of MoS_2 Atomic Layers on a SiO_2 Substrate. *Small* **2012**, *8*, 966–971.

(38) Kim, Y.; Kwon, S.; Seo, E.-J.; Nam, J. H.; Jang, H. Y.; Kwon, S.-H.; Kwon, J.-D.; Kim, D.-W.; Cho, B. Facile Fabrication of a Two-Dimensional TMD/Si Heterojunction Photodiode by Atmospheric-Pressure Plasma-Enhanced Chemical Vapor Deposition. *ACS Appl. Mater. Interfaces* **2018**, *10,* 36136–36143.

(39) Seok, H.; Megra, Y. T.; Kanade, C. K.; Cho, J.; Kanade, V. K.; Kim, M.; Lee, I.; Yoo, P. J.; Kim, H.-U.; Suk, J. W.; Kim, T. Low-Temperature Synthesis of Wafer-Scale MoS_2–WS_2 Vertical Heterostructures by Single-Step Penetrative Plasma Sulfurization. *ACS Nano* **2021**, *2*.

(40) Lim, Y. R.; Song, W.; Han, J. K.; Lee, Y. B.; Kim, S. J.; Myung, S.; Lee, S. S.; An, K. S.; Choi, C. J.; Lim, J. Wafer-Scale, Homogeneous MoS_2 Layers on Plastic Substrates for Flexible Visible-Light Photodetectors. *Adv. Mater.* **2016**, *28*, 5025–5030.

(41) Lin, Z.; Liu, Y.; Halim, U.; Ding, M.; Liu, Y.; Wang, Y.; Jia, C.; Chen, P.; Duan, X.; Wang, C.; Song, F.; Li, M.; Wan, C.; Huang, Y.; Duan, X. Solution-Processable 2D Semiconductors for High-Performance Large-Area Electronics. *Nature* **2018**, *562*, 254–258.

(42) Kelly, A. G.; Hallam, T.; Backes, C.; Harvey, A.; Esmaeily, A. S.; Godwin, I.; Coelho, J.; Nicolosi, V.; Lauth, J.; Kulkarni, A.; Kinge, S.; Siebbeles, L. D. A.; Duesberg, G. S.; Coleman, J. N. All-Printed Thin-

Film Transistors from Networks of Liquid-Exfoliated Nanosheets. *Science* **2017**, *356*, 69–73.

(43) Yu, X.; Prévot, M. S.; Sivula, K. Multiflake Thin Film Electronic Devices of Solution Processed 2D MoS_2 Enabled by Sonopolymer Assisted Exfoliation and Surface Modification. *Chem. Mater.* **2014**, *26*, 5892–5899.
